# ATF4 suppresses hepatocarcinogenesis by inducing SLC7A11 (xCT) to block stress-related ferroptosis

**DOI:** 10.1016/j.jhep.2023.03.016

**Published:** 2023-03-28

**Authors:** Feng He, Peng Zhang, Junlai Liu, Ruolei Wang, Randal J. Kaufman, Benjamin C. Yaden, Michael Karin

**Affiliations:** 1 Academy of Integrative Medicine, Shanghai University of Traditional Chinese Medicine, Shanghai, China;; 2 Laboratory of Gene Regulation and Signal Transduction, Department of Pharmacology, School of Medicine, University of California San Diego, San Diego, CA, USA;; 3 Degenerative Diseases Program, Center for Genetic Disorders and Aging Research, SBP Medical Discovery Institute, La Jolla, CA, USA;; 4 Diabetes Novel Therapies and External Innovation, Eli Lilly and Company, Indianapolis, IN, USA;; 5 Department of Pathology, School of Medicine, University of California San Diego, San Diego, CA, USA

**Keywords:** HCC, Steatohepatitis, ER stress, ATF4, SLC7A11, NRF2, Ferroptosis

## Abstract

**Background & Aims:**

Hepatocellular carcinoma (HCC), a leading cause of cancer-related death, is associated with viral hepatitis, non-alcoholic steatohepatitis (NASH), and alcohol-related steatohepatitis, all of which trigger endoplasmic reticulum (ER) stress, hepatocyte death, inflammation, and compensatory proliferation. Using ER stress-prone *MUP-uPA* mice, we established that ER stress and hypernutrition cooperate to cause NASH and HCC, but the contribution of individual stress effectors, such as activating transcription factor 4 (ATF4), to HCC and their underlying mechanisms of action remained unknown.

**Methods:**

Hepatocyte-specific ATF4-deficient *MUP-uPA* mice (*MUP-uPA/Atf4*^Δhep^) and control *MUP-uPA/Atf4*^F/F^ mice were fed a high-fat diet to induce NASH-related HCC, and *Atf4*^F/F^ and *Atf4*^Δhep^ mice were injected with diethylnitrosamine to model carcinogen-induced HCC. Histological, biochemical, and RNA-sequencing analyses were performed to identify and define the role of ATF4-induced solute carrier family 7a member 11 (SLC7A11) expression in hepatocarcinogenesis. Reconstitution of SLC7A11 in ATF4-deficient primary hepatocytes and mouse livers was used to study its effects on ferroptosis and HCC development.

**Results:**

Hepatocyte ATF4 ablation inhibited hepatic steatosis, but increased susceptibility to ferroptosis, resulting in accelerated HCC development. Although ATF4 activates numerous genes, ferroptosis susceptibility and hepatocarcinogenesis were reversed by ectopic expression of a single ATF4 target, *Slc7a11*, coding for a subunit of the cystine/glutamate antiporter xCT, which is needed for glutathione synthesis. A ferroptosis inhibitor also reduced liver damage and inflammation. *ATF4* and *SLC7A11* amounts were positively correlated in human HCC and livers of patients with NASH.

**Conclusions:**

Despite ATF4 being upregulated in established HCC, it serves an important protective function in normal hepatocytes. By maintaining glutathione production, ATF4 inhibits ferroptosis-dependent inflammatory cell death, which is known to promote compensatory proliferation and hepatocarcinogenesis. Ferroptosis inhibitors or ATF4 activators may also blunt HCC onset.

## Graphical Abstract

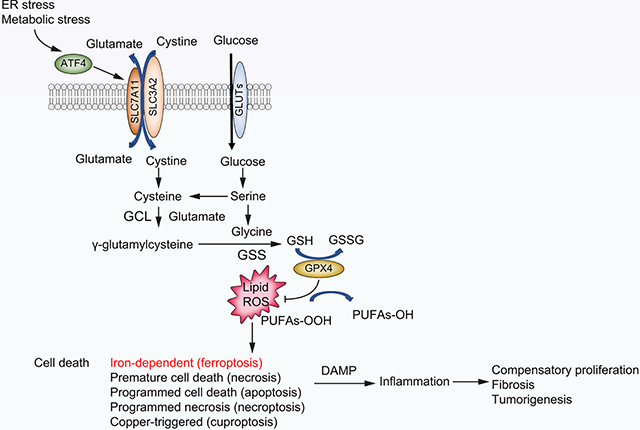

## Introduction

Liver cancer is the third leading cause of cancer death and the sixth most common cancer type worldwide,^1^ whose prevalence constantly increases, paralleling the global rise in obesity, type 2 diabetes, metabolic syndrome, and excessive alcohol intake.^2,3^ Hepatocellular carcinoma (HCC), the main primary liver cancer, is associated with multiple aetiologies, including HBV and HCV infections, non-alcoholic (NAFLD), alcoholic (AFLD), and toxicant-associated (TAFLD) fatty liver diseases, which cause endoplasmic reticulum (ER) stress, liver damage, hepatocyte death, and inflammation. The spectrum of fatty liver disease ranges from simple steatosis without liver damage to steatohepatitis (non-alcoholic steatohepatitis [NASH], alcoholic steatohepatitis [ASH], or toxicant-associated steatohepatitis [TASH] according to their main cause), fibrosis, cirrhosis, and eventually HCC. Despite tight associations with well-defined risk factors, HCC is often diagnosed at an advanced stage, when the main therapeutic options are surgical and radiological ablations and localised chemotherapy. Currently, there are no curative treatments for late-stage HCC, whose median overall survival remains at about 1 year.^4^ Despite advances in NAFLD/NASH, AFLD/ASH, and TAFLD/TASH diagnosis and aetio-logical understanding, the mechanisms that govern progression from steatohepatitis to HCC remain elusive. Oxidative stress, ER stress, cell death, mitochondrial dysfunction, insulin resistance, inflammation, and gut dysbiosis were shown to be involved in steatohepatitis and its progression to HCC.^2,3,5–7^ We established the importance of excessive hepatocyte death, necroinflammation, and compensatory proliferation in HCC development.^5,7,8^ Nonetheless, the type of cell death promoting inflammation and HCC is contentious, with different studies suggesting it could be necrosis, apoptosis, or necroptosis.^9–11^ Ferroptosis is an iron-dependent cell death type that is often accompanied by lipid peroxidation caused by depletion of reduced glutathione (GSH), decreased glutathione peroxidase 4 (GPX4) activity, or increased lipid peroxidation.^12^ Ferroptosis is negatively regulated by solute carrier family 7a member 11 (SLC7A11), GPX4, and GSH. In previous studies conducted with hepatocyte-specific IKKβ knockout mice (*Ikkb*^Δhep^), which are highly susceptible to diethylnitrosamine (DEN)-induced hepatocarcinogenesis, we observed increased lipid peroxidation and hepatocyte death, which were reversed by feeding the mice with N-acetyl cysteine (NAC), a precursor for GSH synthesis.^8^ A key regulator of ferroptosis is transcription factor nuclear factor erythroid 2-related factor 2 (NRF2), whose activation in HCC and elsewhere is promoted by SQSTM1/p62,^5^ which is encoded by an IKKβ/NF-κB-inducible gene.^13^ NRF2 induces *SLC7A11* transcription, which is often elevated in human cancers and accompanied by decreased ferroptosis, increased tumour growth, and drug resistance.^14,15^ Ferroptosis may also be involved in liver injury and inflammation,^16^ but its role in hepatocarcinogenesis is unknown. Here we explored the role of the ER stress mediator activating transcription factor 4 (ATF4) in DEN- and high-fat diet (HFD)-induced HCC in *major urinary protein (MUP)*–*urokinase-type plasminogen activator* (*uPA*) mice, which express the secreted protein uPA from the *MUP* promoter. *MUP-uPA* mice undergo transient hepatic ER stress early in life owing to elevated uPA expression, which is sustained by HFD feeding.^7^ Using chemical chaperones, we demonstrated that NASH development in these mice depends on ER stress.

Protein misfolding after overexpression of a secreted protein triggers the unfolded protein response (UPR), which promotes stress adaptation.^17^ There are three primary UPR effectors: inositol-requiring enzyme 1 (IRE1), which controls expression of X-box-binding protein 1 (XBP1); activating transcription factor 6 (ATF6); and pancreatic ER-regulating kinase (PERK), which phosphorylates eukaryotic translation initiation factor 2 subunit α (eIF2α) to induce ATF4 translation. ATF4 activates numerous target genes, whose products mitigate stress or induce cell death if the stress cannot be resolved.^17^ UPR activation is often observed in cancer cells and thought to provide them with a survival advantage.^17,18^ Hepatocyte-specific IRE1a ablation decreases DEN-induced HCC and blunts acceleration of HCC progression by HFD,^19^ although it increases acute liver injury and hepatic steatosis.^20,21^ In contrast, XBP1 loss promotes tumorigenesis in mouse models of intestinal cancer,^22^ and hepatocyte-specific XBP1 ablation enhanced liver injury and fibrosis but attenuated steatosis in a high-fat/sugar diet-induced NASH model.^23^ ATF6 activates transformation-associated genes during hepatocarcinogenesis,^24^ whereas PERK promotes MYC-driven cell transformation and autophagy.^25^ ATF4 activates genes involved in redox homoeostasis, amino acid (AA) metabolism, protein synthesis, apoptosis, and autophagy,^17,26^ and was extensively studied for its roles in glucose, lipid, and cholesterol metabolism.^27–29^ Systemic ATF4 ablation attenuates fructose-induced hyper-triglyceridaemia and hepatic steatosis,^30,31^ in part through regulation of fibroblast growth factor 21 (FGF21) expression.^32^ In other studies, ATF4 ablation inhibited hepatic steatosis through different mechanisms, including expression of N-nicotinamide methyltransferase (NNMT)^33^, tribbles homologue 3 (TRB3),^34^ or nuclear respiratory factor 1 (NRF1)–mitochondrial transcription factor A (TFAM) signalling.^35^ ATF4 expression is low in healthy cells but is often elevated in cancer, where it promotes cell survival and tumour growth by inducing genes involved in AA metabolism and oxidant defenses.^26,36,37^ To test the role of ATF4 in hepatocarcinogenesis, we generated *Atf4*^Δhep^ mice lacking hepatocyte ATF4. Despite its documented ability to support hepatic steatosis, ATF4 ablation enhanced DEN-induced tumorigenesis and NASH to HCC progression in HFD-fed *MUP-uPA* mice. Notably, the HCC suppressive activity of ATF4 was mainly caused by expression of a single target, SLC7A11, the small subunit of the cystine/glutamate antiporter (xCT), which is needed for GSH synthesis. Ectopic SLC7A11 expression suppressed HCC development in *Atf4*^Δhep^ mice and provided protection from ferroptosis, suggesting that ferroptosis contributes to the HCC-promoting necroinflammatory response and that ferroptosis inhibitors may prevent NASH to HCC progression.

## Materials and methods

### Mouse models

*Atf4*^F/F^ mice, a generous gift from Dr Christopher Adams (University of Iowa, Iowa City, IA, USA),^38^ were intercrossed with Alb-Cre mice (C57BL/6, Jackson Laboratory, Ellsworth, ME, USA) to generate *Atf4*^F/F^-*Alb-Cre* (*Atf4*^Δhep^) mice. *Atf4*^Δhep^ mice were crossbred with *MUP-uPA* mice^7^ to obtain *MUP-uPA/Atf4*^F/F^ and *MUP-uPA/Atf4*^Δhep^ mice. B6.129X1-*Nfe2l2tm1Ywk/J* (*Nrf2*^−/−^) mice were described.^39^ All mouse lines were either on a pure C57BL/6 genetic background or crossed into it for at least 10 generations. The NASH and HCC model: 6-week-old (wo) *MUP-uPA/Atf4*^F/F^ and *MUP-uPA/Atf4*^Δhep^ mice were fed with low-fat diet (LFD) (composed of 12% fat, 23% protein, and 65% carbohydrates based on caloric content) or HFD (consisting of 59% fat, 15% protein, and 26% carbohydrates based on caloric content; Bio-Serv, Flemington, NJ, USA) and sacrificed when 4, 6, and 10 months old (mo) for the pathological analysis. The LFD-fed DEN-induced HCC model: DEN (25 mg/kg, Millipore Sigma N0258, Saint Louis, MO, USA) was injected i.p. into 14-day-old mice, and samples were collected from the LFD-fed mice when 5 and 10 mo. The HFD-fed DEN-induced HCC model: mice were placed on HFD when 3 mo, followed by DEN (80 mg/kg) administration when 4 mo, and water with 0.05% phenobarbital (PB; Sigma, P5178) when 5 mo. The mice were sacrificed and analysed when 10 mo. Animal experiments were performed in accordance with NIH guidelines for the use and care of live animals and approved by the University of California San Diego Institutional Animal Care and Use Committee, S00218. Experiments done at Shanghai University of Traditional Chinese Medicine were performed in accordance with the guidelines of the Ministry of Science and Research of China and approved by the Animal Experimental Ethics Committee of Shanghai University of Traditional Chinese Medicine.

### RNA-seq data processing and analysis

Total liver RNAs were extracted from overnight fasted 6-wo *Atf4*^F/F^, *Atf4*^Δhep^, *MUP-uPA/Atf4*^F/F^, and *MUP-uPA/Atf4*^Δhep^ mice using TRIzol^®^ (Life Technologies, 15596–018, Carlsbad, CA, USA) according to the manufacturer’s instructions and was sequenced on the Illumina NovaSeq 6000 sequencer (2×150 bp read length, San Diego, CA, USA). Raw paired-end reads were trimmed and quality controlled by SeqPrep (https://github.com/jstjohn/SeqPrep) and Sickle (https://github.com/najoshi/sickle) with default parameters. Then the clean reads were separately aligned to a reference genome with orientation mode using HISAT2 (http://ccb.jhu.edu/software/hisat2/index.shtml) software. The mapped reads for each sample were assembled by StringTie (https://ccb.jhu.edu/software/stringtie/index.shtml?t=example) in a reference-based approach. RSEM (http://deweylab.biostat.wisc.edu/rsem/) was used to quantify gene abundance, and differential gene expression analysis was performed using DESeq2/DEGseq/EdgeR. Gene ontology (GO) functional enrichment and Kyoto Encyclopedia of Genes and Genomes (KEGG) pathway analysis were carried out using DAVID Bioinformatics Resources 6.8 (https://david.ncifcrf.gov/). For gene set enrichment analysis (GSEA), the gene expression matrix was pooled from gene expression estimates from StringTie output and processed with mouse-ENSEMBL-gene IDs with 1,000 permutations using a *t* test metric for gene ranking (https://www.gsea-msigdb.org/gsea/index.jsp). Enrichment was tested using default v5.2 MSigDb gene sets. Mouse RNA sequencing (RNA-seq) data have been deposited into the NCBI Gene Expression Omnibus (accession GSE191115).

### Quantification and statistical analysis

Data are shown as mean ± SD as indicated. Statistical significance was determined using the two-tailed Student’s *t* test, and *p* values lower than 0.05 were considered statistically significant (*p <0.05, **p <0.01, ***p <0.001). GraphPad Prism (Boston, MA, USA) was used for statistical analysis and graphing.

Other details can be found in the Supplementary CTAT table and Supplementary information.

## Results

### ATF4 ablation increases uPA-induced liver injury without an effect on UPR activation

**Fig. 1.**
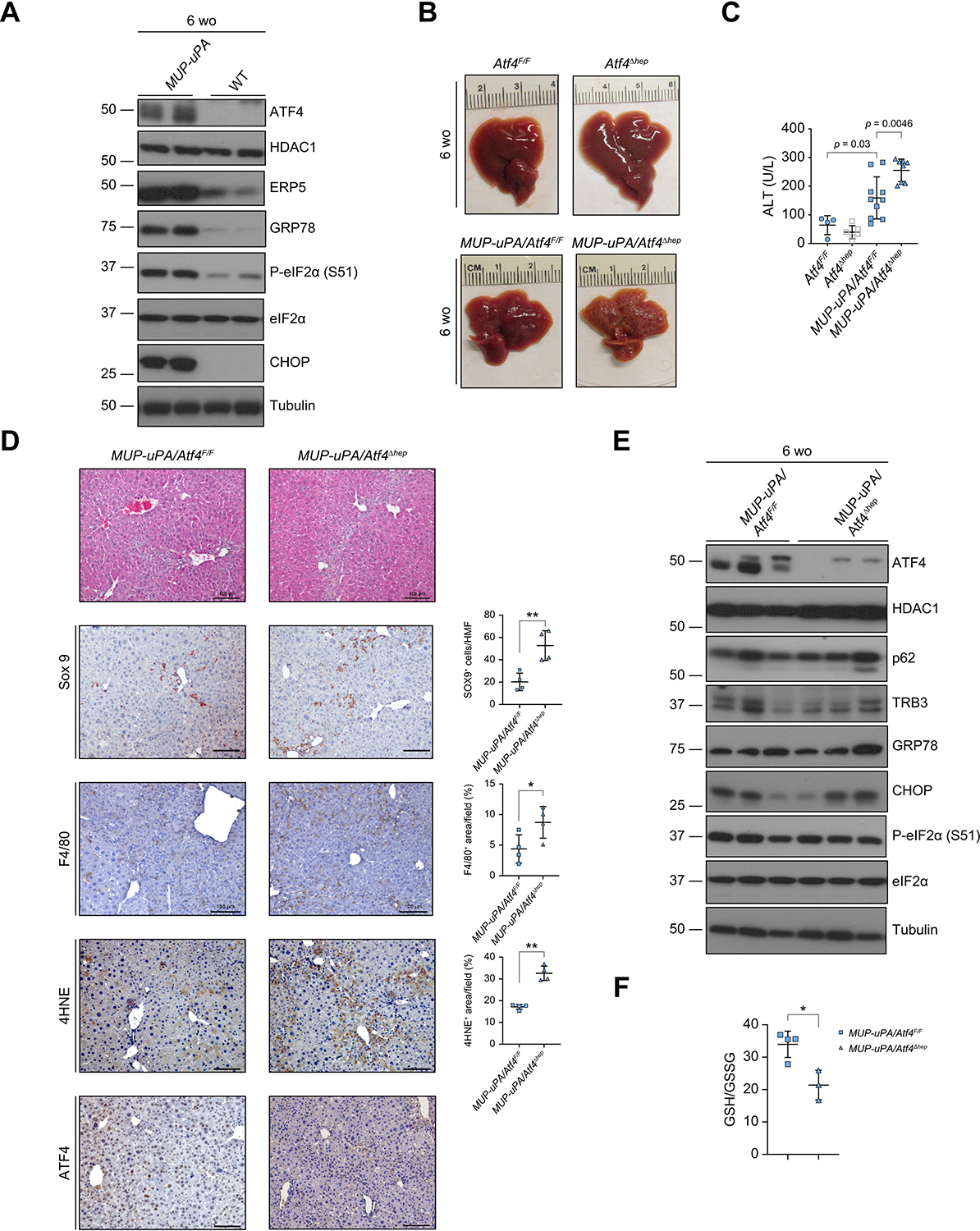
Hepatocyte-specific ATF4 ablation disrupts redox homoeostasis and exacerbates liver injury in MUP-uPA mice. (A) Representative IB analysis of 6-wo BL6 (WT) and *MUP-uPA* mice. (B) Gross liver morphology of 6-wo *Atf4*^F/F^, *Atf4*^Δhep^, *MUP-uPA/Atf4*^F/F^, and *MUP-uPA*/*Atf4*^Δhep^ mice. (C) Serum ALT in above mice. Mean ± SD (n = 4–10/group). Value of *p* indicates significance level, Student’s *t* test. (D) H&E staining and IHC analysis of 6-wo WT, *MUP-uPA/Atf4*^F/F^, and *MUP-uPA/Atf4*^Δhep^ mice. Staining was quantitated as the number of positive cells per HMF or positively stained area per field, as indicated. Mean ± SD (n = 4/group). *p <0.05; **p <0.01 (Student’s *t* test). Scale bars, 100 lm. (E) IB analysis of 6-wo *MUP-uPA/Atf4*^F/F^ and *MUP-uPA/Atf4*^Δhep^ mice. (F) GSH:GSSG ratio in livers of indicated 6-wo mice. Mean ± SD (n = 3–4/group). *p = 0.012 (Student’s *t* test). 4HNE, 4-hydroxynonenal; ATF4, activating transcription factor 4; CHOP, C/EBP-homologous protein; eIF2α, eukaryotic translation initiation factor 2 subunit α; ERP5, endoplasmic reticulum protein 5; GRP78, 78-kDa glucose-regulated protein; GSH, glutathione; GSSG, oxidised glutathione; HDAC1, histone deacetylase 1; HMF, high magnification field; IB, immunoblotting; IHC, immunohistochemistry; MUP-uPA, major urinary protein promoter–urokinase plasminogen activator; P-eIF2α, phosphorylated eIF2α; TRB3, tribbles homologue 3; wo, week old; WT, wild-type.

As previously found,^7^ 5–6-wo *MUP-uPA* mice, whose liver uPA expression had peaked, exhibited elevated expression of ER stress markers, including endoplasmic reticulum protein 5 (ERP5), 78-kDa glucose-regulated protein (GRP78/BIP), C/EBP-homologous protein (CHOP), ATF4, and phosphorylated eIF2α (P-eIF2α) (Fig. 1A and Fig. S1A). To study the role of ATF4 in NASH and HCC development, we generated *Atf4*^Δhep^ mice and crossbred them with *MUP-uPA* mice to obtain *MUP-uPA/Atf4*^Δhep^ mice. Compared with livers of *MUP-uPA/Atf4*^F/F^ mice, *MUP-uPA/Atf4*^Δhep^ livers had a rough appearance with surface nodules (Fig. 1B) and elevated serum alanine aminotransferase (ALT) (Fig. 1C), suggesting enhanced liver injury. By contrast, livers of *Atf4*^Δhep^ mice had normal appearance, and serum ALT was normal, too (Fig. 1B and C). *In situ* hybridisation analysis of *Atf4* mRNA showed elevated expression in *MUP-uPA/Atf4*^F/F^ hepatocytes, and efficient loss of the *Atf4* mRNA signal was observed in both *Atf4*^Δhep^ and *MUP-uPA/Atf4*^Δhep^ mice (Fig. S1B and C), consistent with absence of the floxed allele in isolated hepatocytes (Fig. S1D). Histology revealed hepatocyte damage in 6-wo *MUP-uPA/Atf4*^F/F^ mice accompanied by immune infiltration that was exacerbated in *MUP-uPA/Atf4*^Δhep^ mice (Fig. 1D). Sox9 and F4/80 antibody staining were higher in *MUP-uPA/Atf4*^Δhep^ livers, indicating injury-associated ductular reaction and enhanced macrophage infiltration (Fig. 1D). Notably, immunoblotting (IB) of liver lysates indicated that ATF4 ablation had little or no effect on the ER stress markers, TRB3, GRP78, CHOP, and P-eIF2α (Fig. 1E). The ATF6 and IRE1-Xbp1 branches of the UPR were not affected either based on expression of ATF6 target genes, ER degradation-enhancing alpha-mannosidase-like protein 2 (EDEM2) and GRP78 (Fig. S1E), and similar amounts of spliced *Xbp1* (*Xbp1s*) mRNA in 6-wo *MUP-uPA/Atf4*^F/F^ and *MUP-uPA/Atf4*^Δhep^ mice (Fig. S1F), suggesting that the UPR in *MUP-uPA* mice is activated independently of ATF4. Interestingly, 4-hydroxynonenal (4HNE) staining, a marker of lipid peroxidation and ferroptosis, was elevated in the *MUP-uPA/Atf4*^Δhep^ liver (Fig. 1D) and the reduced GSH to oxidised GSH (GSSG) ratio was decreased (Fig. 1F), suggesting oxidative stress and liver injury. These findings resemble observations made in *Ikkb*^Δhep^ mice, in which HCC development was suppressed by NAC, a GSH precursor.^8^ Quantitative reverse-transcription PCR (qRT-PCR) analysis of *MUP-uPA/Atf4*^Δhep^ livers showed increased expression of F4/80, IL-1α, and C-X-C motif chemokine ligand 2 (CXCL2) mRNAs (Fig. S1G), indicating that ATF4-deficient hepatocytes are more susceptible to ER stress-related necroinflammatory injury, normally suppressed by ATF4, which plays an important role in maintaining redox homoeostasis.

### ATF4 ablation attenuates target gene expression

**Fig. 2.**
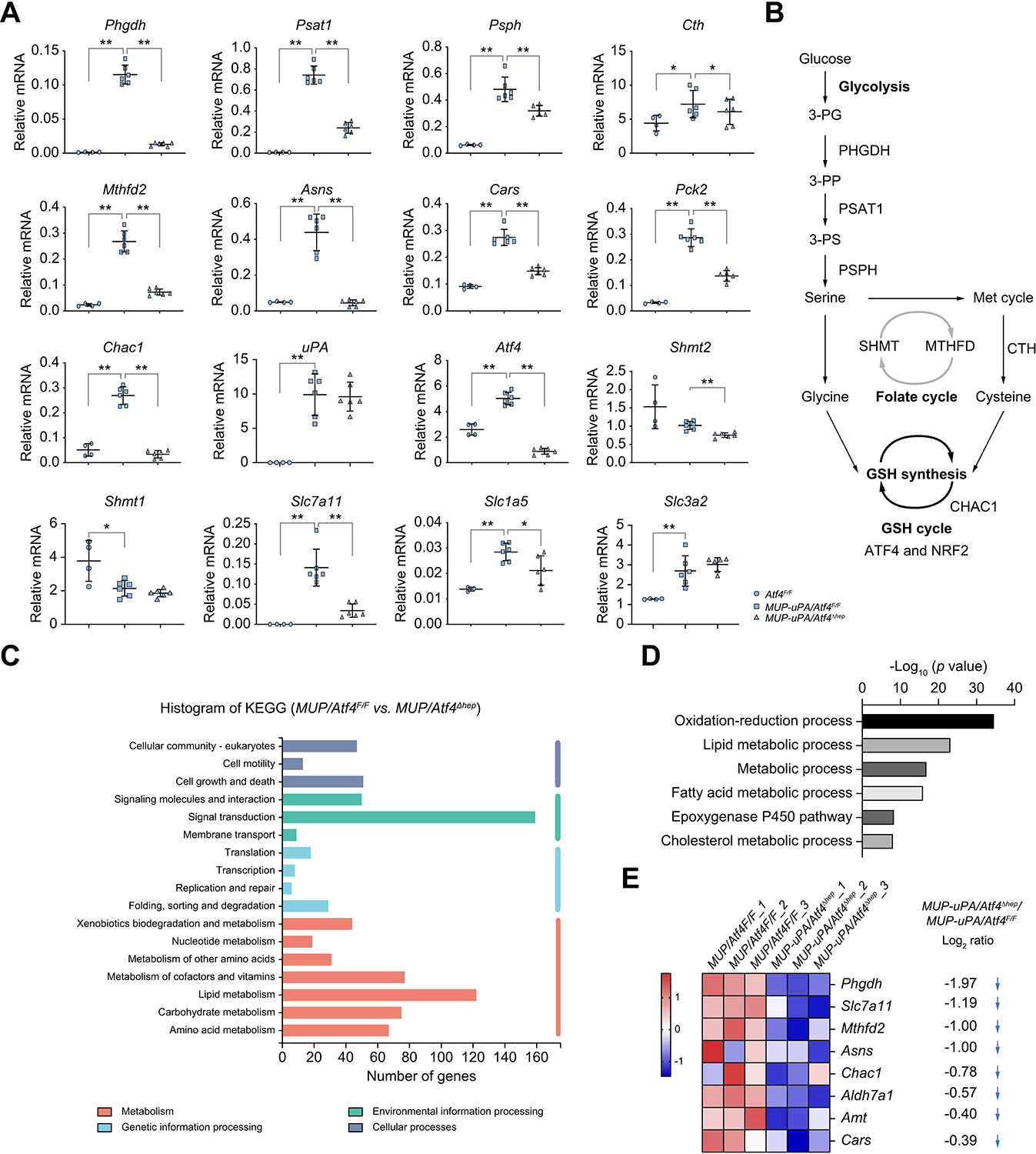
ATF4 ablation disrupts expression of genes involved in amino acid and one-carbon metabolism. (A) qRT-PCR analysis of liver mRNAs from 6-wo *Atf4*^F/F^, *MUP-uPA/Atf4*^F/F^, and *MUP-uPA/Atf4*^Δhep^ mice. Mean ± SD (n = 3–6/group). *p <0.05; **p <0.01 (Student’s *t* test). (B) Diagram showing metabolic pathways with key enzymes involved in serine, glycine, cysteine, and GSH metabolism as well as the folate and methionine cycles. (C) KEGG pathway analysis of genes differentially represented in RNA-seq data from livers of overnight fasted 6-wo *MUP-uPA/Atf4*^Δhep^ and *MUP-uPA/Atf4*^F/F^ mice (n = 3). (D) DAVID-based GO analysis of differentially expressed genes in above RNA-seq data (n = 3). (E) Heat map representation of significantly downregulated genes in *MUP-uPA/Atf4*^Δhep^ relative to *MUP-uPA/Atf4*^F/F^ livers (n = 3) associated with ‘amino acid metabolism’ including Log_2_FC value. ATF4, activating transcription factor 4; CHAC1, glutathione specific gamma-glutamylcyclotransferase 1; CTH, cystathionine gamma-lyase; GO, gene ontology; GSH, glutathione; KEGG, Kyoto Encyclopedia of Genes and Genomes; Log_2_FC, log_2_ fold-change; MTHFD2, methenyltetrahydrofolate dehydrogenase 2; MUP-uPA, major urinary protein promoter–urokinase plasminogen activator; NRF2, nuclear factor erythroid 2-related factor 2; PHGDH, phosphoglycerate dehydrogenase; PSAT1, phosphoserine aminotransferase 1; PSPH, phosphoserine phosphatase; qRT-PCR, quantitative reverse-transcription PCR; RNA-seq, RNA sequencing; SHMT, serine hydroxymethyltransferase; 3-PG, 3-phosphoglycerate; 3-PP, 3-phosphohydroxypyruvate; 3-PS, 3-phosphoserine; wo, week old.

Young *MUP-uPA* hepatocytes are ER-stressed, undergoing cell death and self-renewal.^7,40^ We used qRT-PCR to examine how ATF4 ablation affects target gene expression. *MUP-uPA/Atf4*^F/F^ livers exhibited elevated expression of phosphoglycerate dehydrogenase (PHGDH), phosphoserine aminotransferase 1 (PSAT1), phosphoserine phosphatase (PSPH), cystathionine gamma-lyase (CTH), and methenyltetrahydrofolate dehydrogenase 2 (MTHFD2) mRNAs relative to wild-type (WT) livers (Fig. 2A). The products of these mRNAs catalyse serine and cysteine synthesis as well as one carbon metabolism/folate cycle (Fig. 2B). ATF4 ablation reduced these mRNAs (Fig. 2A). *MUP-uPA/Atf4*^F/F^ livers also showed elevated expression of mRNAs for asparagine synthetase (ASNS), cysteinyl-tRNA synthetase (CARS), mitochondrial phosphoenolpyruvate carboxykinase (PCK2), and glutathione-specific gamma-glutamylcyclotransferase 1 (CHAC1), enzymes involved in AA metabolism, gluconoeogenesis, and GSH utilisation, all of which were reduced in *MUP-uPA/Atf4*^Δhep^ livers (Fig. 2A). Decreased gene expression was not caused by reduced uPA expression or less ER stress in *MUP-uPA/Atf4*^Δhep^ livers (Fig. 2A). *Atf4* mRNA was higher in *MUP-uPA/Atf4*^F/F^ livers compared with WT livers.

GSH, whose amounts were reduced in *MUP-uPA/Atf4*^Δhep^ livers, is a key antioxidant and redox regulator.^41,42^ *De novo* GSH synthesis is mediated via a two-step biosynthetic process catalysed by glutamate–cysteine ligase (GCL) and glutathione synthase (GSS). mRNAs for enzymes involved in glycine, cysteine, and glutamate synthesis were reduced on ATF4 ablation (Fig. 2A), which did not significantly affect mRNAs for glutaminolysis enzymes, glutaminase liver isoform (GSL2), and glutamate dehydrogenase 1 (GLUD1) (Fig. S2A). ATF4 ablation reduced mRNA for serine hydroxymethyltransferase 2 (SHMT2), which catalyses serine and glycine interconversion, but not for SHMT1 (Fig. 2A). Cysteine is the limiting GSH precursor, and its uptake is mediated by xCT.^12,42^ Liver-specific ATF4 ablation reduced expression of mRNAs for SLC7A11 and SLC1A5, which encodes the neutral AA transporter B (ASCT2) for uptake of alanine, serine, cysteine, and threonine, but had little effect on SLC1A4/ASCT1 mRNA amounts (Fig. 2A and Fig. S2A). ATF4 deletion also reduced the mRNAs for the large neutral AA transporter small subunit 1 (SLC7A5/4F2LC), but not for the heavy subunit of the large neutral AA transporter (SLC3A2/4F2HC), and seryl-tRNA synthetase (SARS) (Fig. 2A and Fig. S2A).

KEGG pathway analysis of RNA-Seq data collected from overnight-fasted 6-wo *MUP-uPA/Atf4*^F/F^ and *MUP-uPA/Atf4*^Δhep^ livers revealed that differentially expressed genes involved in lipid metabolism, metabolism of cofactors and vitamins, carbohydrate metabolism, and AA metabolism were the top four differentially expressed metabolic groups (Fig. 2C). DAVID-based GO analysis showed that top four significant changes in biological processes upon ATF4 ablation were oxidation–reduction process, lipid metabolic process, metabolic process, and fatty acid metabolic process (Fig. 2D). GSEA indicated that ATF4 ablation reduced expression of genes involved in AA metabolism (Fig. S2B). Heat map depiction showed significantly downregulated ‘AA metabolism’-related genes, including those encoding PHGDH, SLC7A11, MTHFD2, ASNS, glutathione-specific gamma-glutamylcyclotransferase 1 (CHAC1), alpha-aminoadipic semialdehyde dehydrogenase (ALDH7A1), aminomethyltransferase (AMT), and CARS in *MUP-uPA/Atf4*^Δhep^ relative to *MUP-uPA/Atf4*^F/F^ livers (Fig. 2E). By contrast, the *Atf4*^F/F^ and *Atf4*^Δhep^ liver transcriptomes were barely different (Fig. S2C and D), correlating with low ATF4 expression in non-stressed livers. These data explain how ATF4 ablation decreases the GSH:GSSG ratio.

The major transcriptional regulator of redox homoeostasis is NRF2.^41,43^ ER stress and oxidative stress are closely linked,^43^ and *MUP-uPA/Atf4*^F/F^ mice showed elevated expression of numerous NRF2 target genes encoding SLC7A11, SLC1A4, SLC7A5, haem oxygenase 1 (HO1), NAD(P)H quinone dehydrogenase 1 (NQO1), and GSS (Fig. 2A, and Fig. S2A and E). ATF4 ablation had no effect on HO1, NQO1, GSS, GCLC, and GCLM mRNAs, while reducing SLC7A11, SLC7A5, and superoxide dismutase 2 (SOD2) mRNA amounts (Fig. 2A, and Fig. S2A and E), suggesting that some NRF2 targets also respond to ATF4. By contrast, ATF4 ablation increased expression of the pro-apoptotic mRNAs for Bcl-2-associated X protein (BAX) and death receptor 5 (DR5) but decreased expression of p53 upregulated modulator of apoptosis (PUMA) and had no effect on expression of BH3-interacting domain death agonist (BID), tumour necrosis factor receptor 1 (TNFR1), tumour necrosis factor receptor superfamily member 6 (TNFRSF6/FAS), and CHOP mRNAs (Fig. S2F). These results suggest that ATF4 ablation may contribute to apoptosis under stress.

### Reduced SLC7A11 expression increases susceptibility to ferroptosis

**Fig. 3.**
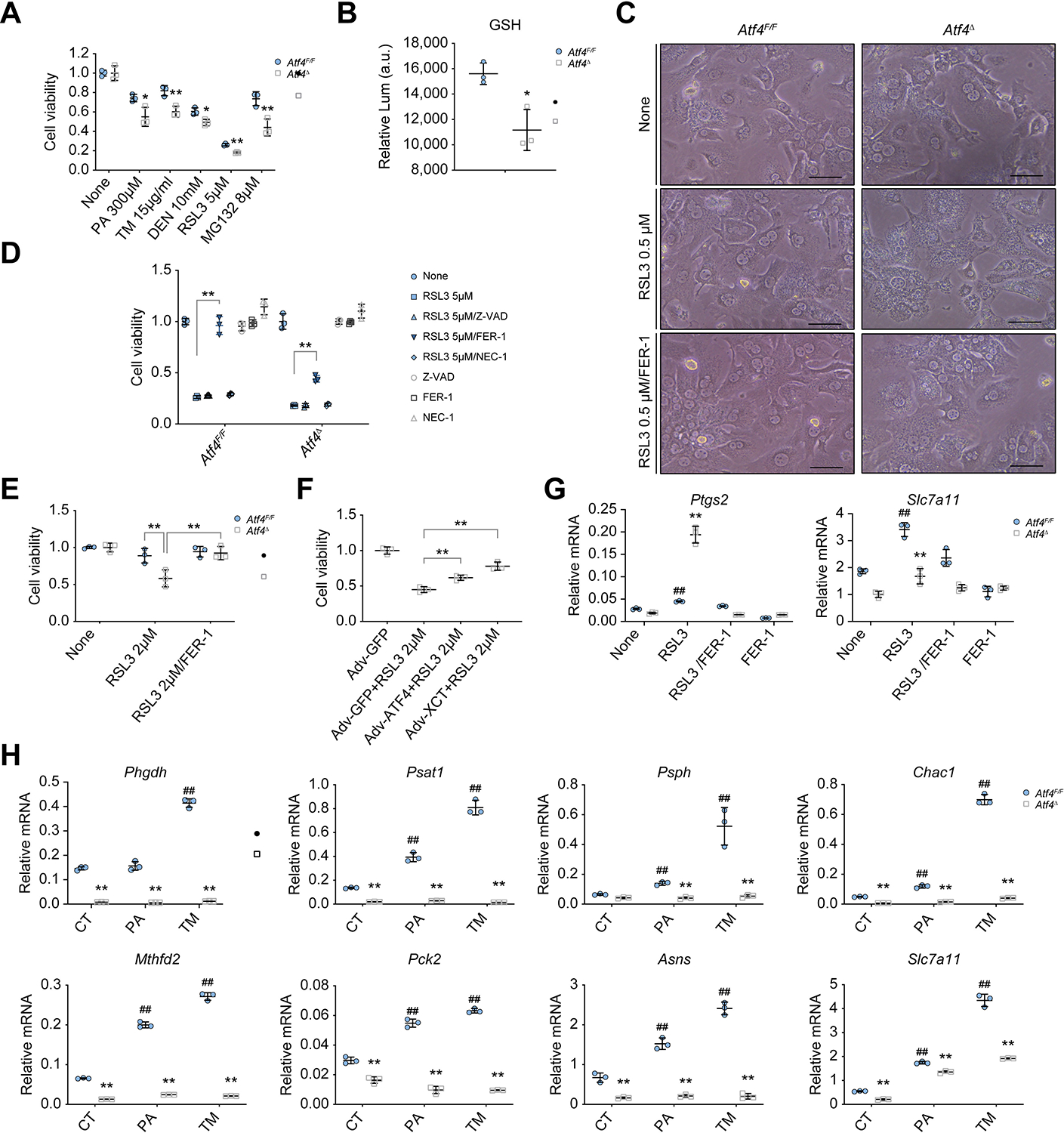
ATF4-deficient hepatocytes are ferroptosis susceptible. (A) Viability of *Atf4*^F/F^ and *Atf4*^Δ^ hepatocytes exposed to different cell death inducers. *p <0.05; **p <0.01 (*vs. Atf4*^F/F^, Student’s *t* test). (B) Relative GSH levels in above hepatocytes. (C) Representative images showing morphology of *Atf4*^F/F^ and *Atf4*^Δ^ hepatocytes treated with vehicle control (NONE) or 0.5 μM RSL3 in the absence or presence of 10 μM FER-1 for 10 h. Scale bars, 50 μm. (D) Viability of above hepatocytes treated without or with 5 μM RSL3 in the absence or presence of 50 μM Z-VAD, 10 μM FER-1, or 10 μM NEC-1 for 10 h. (E) Viability of *Atf4*^F/F^ and *Atf4*^Δ^ hepatocytes treated without or with 2 μM RSL3 or 2 μM RSL3 plus 10 μM FER-1 for 10 h. (F) Viability of *Atf4*^Δ^ hepatocytes transduced with Adv-GFP, Adv-ATF4, or Adv-xCT 24 h before culturing in the presence of 2 μM RSL3, as indicated. **p <0.01 (Student’s *t* test). (G) qRT-PCR analysis of *Ptgs2* and *Slc7a1*1 mRNAs in *Atf4*^F/F^ and *Atf4*^Δ^ hepatocytes treated with NONE, 0.5 μM RSL3, 10 μM FER-1, or a 0.5 μM RSL3 plus 10 μM FER-1 combo for 8 h. **p <0.01 (*vs. Atf4*^F/F^, Student’s *t* test). ^##^*p* <0.01 (*vs*. NONE of *Atf4*^F/F^, Student’s *t* test). (H) qRT-PCR analysis of mRNA expression in *Atf4*^F/F^ and *Atf4*^Δ^ hepatocytes treated with NONE, 300 μM PA, or 4 μg/ml TM for 10 h. Mean ± SD (n = 3/group). **p <0.01 (*vs. Atf4*^F/F^, Student’s *t* test). ^##^*p* <0.01 (*vs*. CT of *Atf4*^F/F^, Student’s *t* test). Adv, adenovirus; AFT4, activating transcription factor 4; CT, Control; DEN, diethylnitrosamine; FER-1, ferrostatin-1; GSH, reduced glutathione; Lum, luminescence; NEC-1, necrostatin-1; PA, palmitic acid; RSL3, RAS-selective lethal 3; TM, tunicamycin.

The *MUP-uPA/Atf4*^Δhep^ liver exhibited features of ferroptosis that could be attributed to reduced expression of xCT, a key regulator of ferroptosis.^12^ To examine whether ATF4 ablation increased susceptibility to ferroptosis and other forms of cell death, we cultured *Atf4*^F/F^ and ATF4-deficient (*Atf4*^Δ^) hepatocytes and treated them with different cell death inducers, including palmitic acid (PA), tunicamycin (TM), DEN, the ferroptosis inducer RAS-selective lethal 3 (RSL3), and the proteasome inhibitor MG132. Although *Atf4*^Δ^ hepatocytes grew as well as *Atf4*^F/F^ hepatocytes under basal conditions, they exhibited higher cell death rates in response to drug treatment and had lower intracellular GSH (Fig. 3A and B, and Fig. S3A). NRF2-deficient (*Nfe2l2*^−/−^) hepatocytes were also more susceptible to RSL3-induced cell death than WT hepatocytes (Fig. S3B). Although most *Atf4*^Δ^ hepatocytes died after incubation with low RSL3 concentrations, most *Atf4*^F/F^ hepatocytes remained viable, but when RSL3 amounts were high, both *Atf4*^F/F^ and *Atf4*^Δ^ hepatocytes died (Fig. 3C–E and Fig. S3C). The ferroptosis inhibitor ferrostatin-1 (FER-1) completely inhibited RSL3-induced *Atf4*^F/F^ hepatocyte death, but the caspase inhibitor Z-VAD or the necroptosis inhibitor necrostatin-1 (NEC-1) were ineffective (Fig. 3D). Full protection of *Atf4*^Δ^ hepatocytes was seen in cells incubated with a low RSL3 concentration (Fig. S3D). Transmission electron microscopy (TEM) showed a typical feature of ferroptosis, smaller mitochondria, present in RSL3-treated hepatocytes, especially in the absence of ATF4 (Fig. S3E), consistent with previous reports.^12,16^ RSL3-induced ferroptosis of *Atf4*-deficient hepatocytes was also inhibited by the iron chelator deferoxamine (DFO) (Fig. S3F). β-Mercaptoethanol (β-Me), however, had little effect on *Atf4*^F/F^ hepatocytes, consistent with previous reports,^12,44^ although it slightly inhibited ferroptosis in *Atf4*^Δ^ hepatocytes (Fig. S3F). Furthermore, FER-1 partly inhibited the DEN-induced cell death of *Atf4*^Δ^ hepatocytes (Fig. S3G), suggesting that DEN triggers different forms of cell death, including ferroptosis.

To determine whether elevated ferroptosis susceptibility of *Atf4*^Δ^ hepatocytes is caused by their SLC7A11 deficiency, we used adenovirus (Adv)-mediated transduction to express GFP, ATF4, or SLC7A11. Reconstitution of either ATF4 or SLC7A11 provided protection against RSL3-mediated ferroptosis, with SLC7A11 being more efficient owing to its higher expression from the Adv promoter than from the ATF4-activated endogenous promoter (Fig. 3F and Fig. S3H). RSL3-mediated oxidative stress led to NRF2 activation and induction of the NRF2 target prostaglandin G/H synthase 2 (PTGS2), a ferroptosis marker.^16,45^ Accordingly, ATF4 ablation increased PTGS2 and decreased SLC7A11 mRNA expression in RSL3-treated cells (Fig. 3G). These results suggest that ATF4 provides protection from ferroptosis by inducing SLC7A11 expression. PA or TM treatment of *Atf4*^F/F^ and *Atf4*^Δ^ hepatocytes resulted in strong induction of PHGDH, PSAT1, PSPH, CHAC1, MTHFD2, PCK2, ASNS, SLC7A11, FGF21, SLC7A5, and SLC1A4 mRNAs in *Atf4*^F/F^ hepatocytes, although *Atf4*^Δ^ hepatocytes were barely or modestly responsive (Fig. 3H and Fig. S3I). These results are consistent with those observed in *MUP-uPA/Atf4*^F/F^ and *MUP-uPA/Atf4*^Δhep^ mice.

### ATF4 and NRF2 coordinately control SLC7A11 expression

**Fig. 4.**
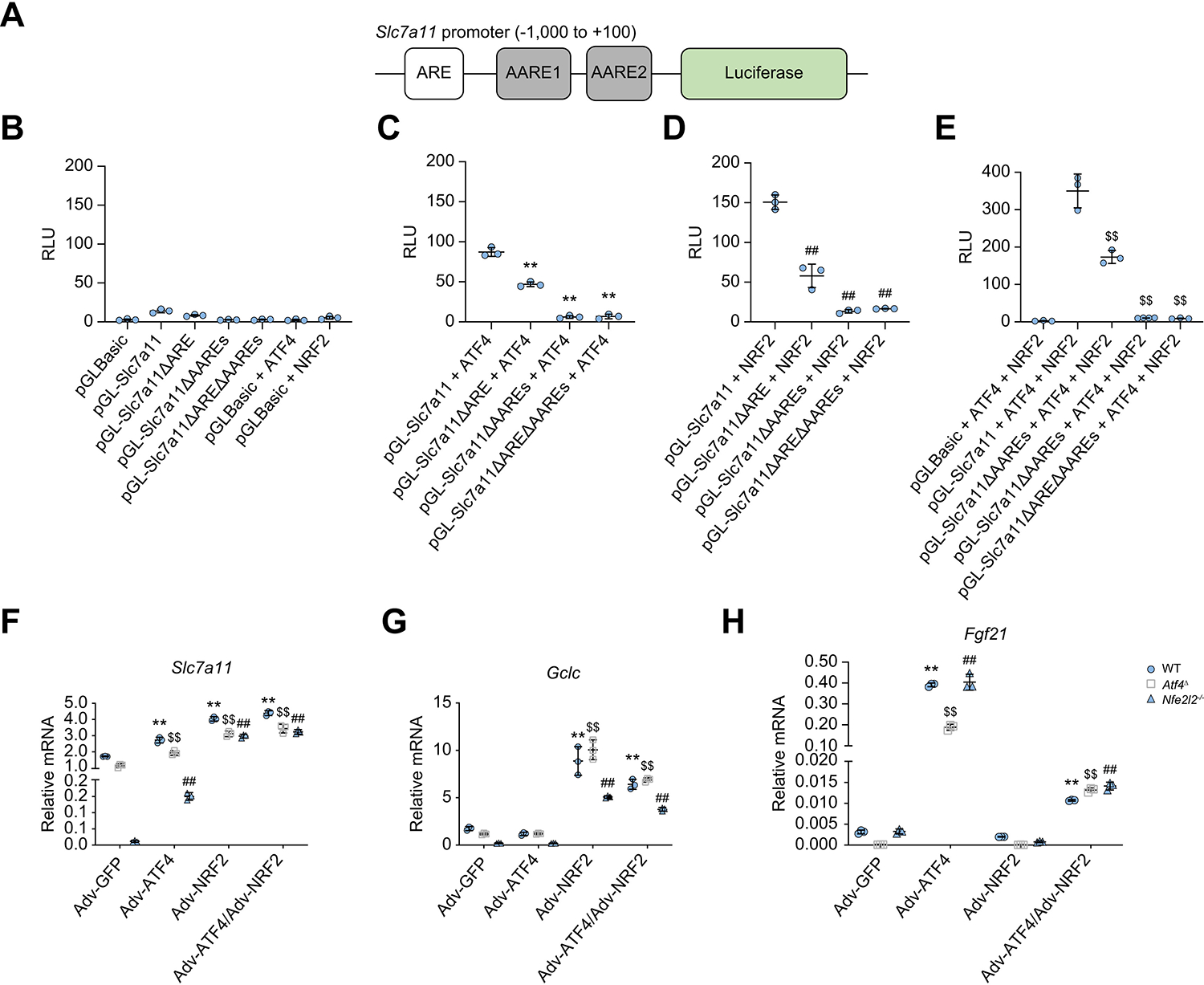
ATF4 and NRF2 coordinately regulate Slc7a11 expression. (A) Schematic representation of the mouse *Slc7a11* gene promoter with the upstream ARE and the two AAREs (AARE-1 and AARE-2) indicated by open and solid rectangles, respectively, fused to a luciferase reporter. (B–E) Luciferase assay measuring *Slc7a11* promoter activity before and after ARE and AARE deletion (ΔARE, ΔAAREs, and ΔAREΔAAREs) in the absence (B) or presence of ATF4 (C), NRF2 (D), and ATF4 + NRF2 (E). HEK293 cells were cotransfected with reporter plasmids (pGLBasic, pGL-Slc7a11, and its variants) in combination with pAd-track-NRF2 and/or ATF4 expression vectors, and a control Renilla luciferase vector PRL-TK. After 24 h, luciferase activities were measured and normalised to Renilla luciferase activity and presented as RLU (mean ± SD from three independent experiments). **p <0.01 (*vs*. pGL-Slc7a11 + ATF4); ^##^*p* <0.01 (*vs*. pGL-Slc7a11 + NRF2); ^$$^*p* <0.01 (*vs*. pGL-Slc7a11 + ATF4 + NRF2), Student’s *t* test. (F–H) qRT-PCR analysis of *Slc7a11* (F), *Gclc* (G), and *Fgf21* (H) mRNAs in WT, *Atf4*^Δ^, and *Nfe2l2*^−/−^ hepatocytes transduced with Adv-GFP, Adv-ATF4, Adv-NRF2, or Adv-ATF4 + NRF2. Mean ± SD (n = 3/group). **p <0.01 (*vs*. Adv-GFP of WT hepatocytes); ^$$^*p* <0.01 (*vs*. Adv-GFP of *Atf4*^Δ^ hepatocytes); ^##^*p* <0.01 (*vs*. Adv-GFP of *Nfe2l2*^−/−^ hepatocytes), Student’s *t* test. AARE, amino acid response element; Adv, adenovirus; ARE, antioxidant response element; ATF4, activating transcription factor 4; NRF2, nuclear factor erythroid 2-related factor 2; qRT-PCR, quantitative reverse-transcription PCR; RLU, relative luminescence unit; SLC7A11, solute carrier family 7a member 11; WT, wild-type.

NRF2 activates the antioxidant response by binding to antioxidant response elements (AREs) in its target promoters,^46^ whereas ATF4 controls AA metabolism by binding to AA response elements (AAREs).^36^ Previous studies in cancer cells showed some overlap between NRF2 and ATF4 target genes, including CHOP/*Ddit3*^47,48^ and HO1.^49^ The *Slc7a11* promoter contains one ARE and two AAREs.^50,51^ To understand how ATF4 and NRF2 regulate SLC7A11 expression in non-transformed hepatocytes, we generated a series of ARE and AARE deletion mutations in the *Slc7a11* promoter fused to a luciferase reporter and examined their response to cotransfected ATF4 and NRF2 expression vectors (Fig. 4A). Either ATF4 or NRF2 stimulated luciferase expression, and the two combined gave the highest luciferase expression (Fig. 4B–E). Deletion of either the ARE or AAREs decreased luciferase expression to various degrees, and deletion of both elements completely blocked luciferase expression (Fig. 4B–E).

We reconstituted WT, *Atf4*^Δ^, and *Nfe2l2*^−/−^ hepatocytes with Adv-GFP, Adv-ATF4, Adv-NRF2, or Adv-ATF4 + NRF2 and analysed target gene expression (Fig. S4A). Relative to Adv-GFP, either Adv-ATF4 or Adv-NRF2 induced *Slc7a1*1 mRNA in WT and *Atf4*^Δ^ hepatocytes, but the response to Adv-ATF4 was suppressed in *Nfe2l2*^−/−^ hepatocytes, a defect that was overcome by Adv-NRF2 infection (Fig. 4F). This suggests that although NRF2 is a potent *Slc7a11* inducer, maximal gene induction depends on ATF4, but without NRF2, ATF4 does not induce *Slc7a1*1 mRNA. By contrast, ATF4 was not needed for induction of the typical NRF2 target genes *Gclc* and *Ptgs2* (Fig. 4G and Fig. S4B). The typical ATF4 target gene *Fgf21* was responsive to Adv-ATF4 but not to Adv-NRF2, which attenuated the response to ATF4 (Fig. 4H). Congruently, NRF2 ablation had no effect on *Fgf21* induction by Adv-ATF4.

### ATF4 ablation increases NASH severity and HCC burden in *MUP-uPA* mice

**Fig. 5.**
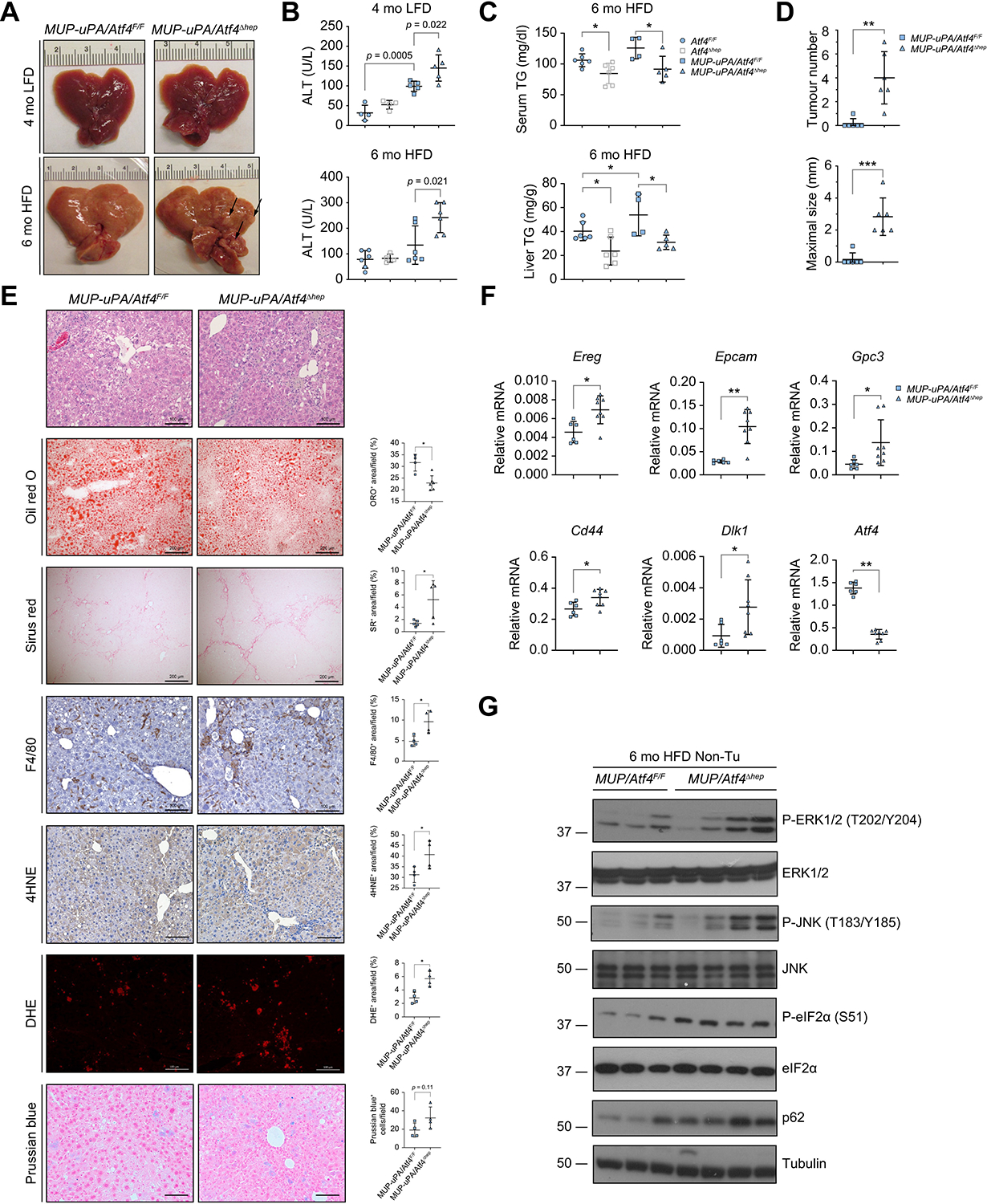
Hepatocyte-specific ATF4 ablation increases lipid peroxidation, liver damage, inflammation, and HCC in HFD-fed MUP-uPA mice. (A) Gross liver morphology of 4-mo LFD-fed and 6-mo HFD-fed *MUP-uPA/Atf4*^F/F^ and *MUP-uPA/Atf4*^Δhep^ mice. Arrows indicate tumours. (B) Serum ALT in 4-mo LFD-fed and 6-mo HFD-fed *Atf4*^F/F^, *Atf4*^Δhep^, *MUP-uPA/Atf4*^F/F^, and *MUP-uPA/Atf4*^Δhep^ mice. Mean ± SD (n = 4–6/group). Value of *p* indicates significance level, Student’s *t* test. (C) Serum and liver TG in 6-mo HFD-fed *Atf4*^F/F^, *Atf4*^Δhep^, *MUP-uPA/Atf4*^F/F^, and *MUP-uPA/Atf4*^Δhep^ mice. Mean ± SD (n = 4–6/group). *p <0.05 (Student’s *t* test). (D) Tumour numbers and maximal tumour sizes in livers of 6-mo HFD-fed *MUP-uPA/Atf4*^F/F^ and *MUP-uPA/Atf4*^Δhep^ mice. Mean ± SD (n = 6/group). **p <0.01; ***p <0.001 (Student’s *t* test). (E) H&E, Oil red O, Sirius Red, F4/80, 4HNE, DHE, and Prussian blue staining of liver sections from above mice. Staining was quantitated as the number of positive cells per HMF or positively stained area per field, as indicated. Scale bars, 100 μm for H&E, IHC, DHE, and Prussian blue staining; 200 μm for Oil Red O and Sirius Red staining. Mean ± SD (n = 6/group). *p <0.05 (Student’s *t* test). (F) qRT-PCR analysis of HCC markers in above mice. Mean ± SD (n = 6–8/group). *p <0.05; **p <0.01 (Student’s *t* test). (G) Representative IB analysis of Non-Tu liver tissues of above mice (n = 3–4/group). 4HNE, 4-hydroxynonenal; ALT, alanine aminotransferase; ATF4, activating transcription factor 4; DHE, dihydroethidium; eIF2α, eukaryotic translation initiation factor 2 subunit α; ERK1/2, extracellular signal-regulated kinase 1/2; HCC, hepatocellular carcinoma; HFD, high fat diet; HMF, high magnification field; IHC, immunohistochemistry; JNK, Jun N-terminal kinase; LFD, low-fat diet; mo, month old; MUP-uPA, major urinary protein promoter–urokinase plasminogen activator; Non-Tu, non-tumours; PB^+^, Prussian blue positive; P-ERK1/2, phosphorylated ERK1/2; P-eIF2α, phosphorylated eIF2α; P-JNK, phosphorylated JNK; qRT-PCR, quantitative reverse-transcription PCR; TG, triglycerides.

*MUP-uPA/Atf4*^F/F^ and *MUP-uPA/Atf4*^Δhep^ mice were kept on either LFD or HFD to examine NASH development and HCC progression. Livers of LFD-fed 4-mo *MUP-uPA/Atf4*^F/F^ mice had a normal appearance, but those of *MUP-uPA/Atf4*^Δhep^ mice retained a rough appearance, exhibited regenerative nodules, and had elevated serum ALT (Fig. 5A and B), indicating more liver damage. Histological analysis revealed more liver fibrosis, proliferating hepatocytes, lipid peroxidation, and cell death in *MUP-uPA/Atf4*^Δhep^ relative to *MUP-uPA/Atf4*^F/F^ mice (Fig. S5A). *MUP-uPA/Atf4*^Δhep^ livers showed higher expression of *Il6*, *Ly6g*, and *Dr5* mRNAs, suggesting increased monocyte, granulocyte, and neutrophil content (Fig. S5B). *MUP-uPA/Atf4*^Δhep^ livers showed large reduction in *Atf4* and *Fgf2*1 mRNAs, but no effect on *Tnf* mRNA (Fig. S5B).

Similar to *MUP-uPA* mice,^7^ HFD-fed 6-mo *MUP-uPA/Atf4*^F/F^ mice displayed typical NASH signs, including immune infiltration and fibrosis, which were more pronounced in *MUP-uPA/Atf4*^Δhep^ mice, that also presented with liver tumour nodules and high circulating ALT (Fig. 5A–E). However, ATF4 ablation decreased serum triglyceride (TG) and total cholesterol (T-Chol), and liver TG, but not liver T-Chol (Fig. 5C and Fig. S5C). Notably, lipid droplet accumulation in *MUP-uPA/Atf4*^Δhep^ livers was reduced relative to *MUP-uPA/Atf4*^F/F^ livers (Fig. 5E), a situation typical of advanced human NASH.^3,52^ ATF4 ablation also accelerated and augmented HCC development, which was not apparent in *MUP-uPA/Atf4*^F/F^ mice of a similar age (Fig. 5D), and increased lipid peroxidation (Fig. 5E). Prussian blue staining showed the presence of liver ferric iron, which contributes to ferroptosis induction, in both livers, which was not affected by ATF4 ablation. qRT-PCR analysis revealed increased expression of mRNAs coding for the fibrogenic cytokine transforming growth factor beta (TGFβ) and several fibrosis markers as well as inflammatory cytokines and chemokines, tumour necrosis factor (TNF), IL-1β, C–C motif chemokine ligand 2 (CCL2), CCL19, and IL-23A in the *MUP-uPA/Atf4*^Δhep^ livers (Fig. S5D and E). Consistent with HCC development, *MUP-uPA/Atf4*^Δhep^ livers exhibited elevated expression of mRNAs for HCC markers, epiregulin (EREG), epithelial cell adhesion molecule (EPCAM), glypican-3 (GPC3), CD44, and protein delta homologue 1 (DLK1) (Fig. 5F). ATF4 deficiency increased phosphorylation of extracellular signal-regulated kinase 1/2 (ERK1/2) at threonine 202 and tyrosine 204, Jun N-terminal kinase (JNK) at threonine 183 and tyrosine 185, and eIF2α at serine 51, as well as p62 expression (Fig. 5G), which supports HCC development in *MUP-uPA* mice.^5^ Expression and phosphorylation of signal transducer and activator of transcription 3 (STAT3), AKT, and JNK, but not of ERK, were also increased in *MUP-uPA/Atf4*^Δhep^ tumours isolated from 10-mo mice (Fig. S5F). *MUP-uPA/Atf4*^Δhep^ HCCs were all ATF4 deficient (Fig. S5G), indicating they originated from ATF4-deficient hepatocytes. These data indicate that despite the reduction in hepatic steatosis, ATF4 deletion accelerates NASH pathogenesis and NASH to HCC progression.

### ATF4 ablation enhances DEN-induced liver injury and tumorigenesis

DEN is a hepatotoxin and carcinogen that activates liver UPR signalling and increases ATF4 expression during HCC development.^53^ To examine the effect of ATF4 ablation on chemical liver injury and carcinogenesis, we challenged 3-mo *Atf4*^F/F^ and *Atf4*^Δhep^ mice with high-dose DEN (100 mg/kg). DEN is metabolically activated by cytochrome P450 2E1 (CYP2E1) in pericentral zone 3 hepatocytes.^5,54^ Accordingly, 24 h after DEN administration, *Atf4*^F/F^ livers exhibited pericentral damage, whereas *Atf4*^Δhep^ livers showed broader damage extending from zone 3 to zone 2; increased proliferating cell nuclear antigen (PCNA), F4/80, TUNEL, phosphorylated (γ) histone H2AX, and 4HNE staining; and elevated serum ALT (Fig. S6A–C). ATF4 ablation, however, did not affect CYP2E1 expression (Fig. S6A, D and E), suggesting that enhanced liver damage in *Atf4*^Δhep^ mice is not caused by altered DEN metabolism.

**Fig. 6.**
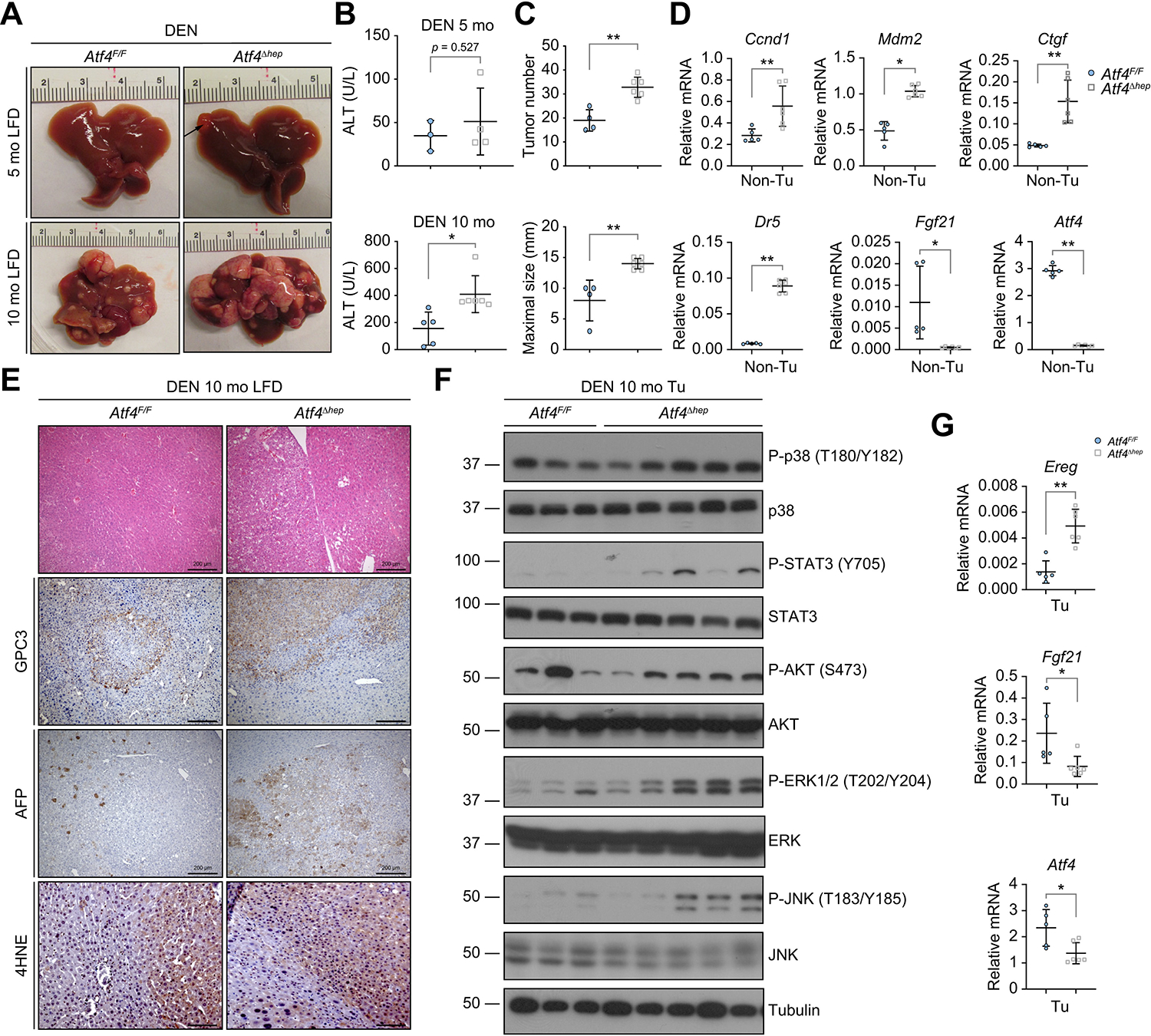
ATF4 ablation accelerates DEN-induced HCC onset and burden. (A) Gross liver morphology of DEN-treated 5- and 10-mo LFD-fed *Atf4*^F/F^ and *Atf4*^Δhep^ mice. (B) Serum ALT in DEN-treated 5-mo (top) and 10-mo (bottom) *Atf4*^F/F^ and *Atf4*^Δhep^ mice. Mean ± SD (n = 3–6/group). *p <0.05; **p <0.01 (Student’s *t* test). (C) Liver tumour number (top) and maximal tumour size (bottom) in above 10-mo mice. Mean ± SD (n = 4–6/group). **p <0.01 (Student’s *t* test). (D) qRT-PCR analysis of mRNAs in the Non-Tu region of above 5-mo mice. Mean ± SD (n = 5–6/group). *p <0.05; **p <0.01 (Student’s *t* test). (E) H&E, GPC3, AFP, and 4HNE staining of liver sections from above 10-mo mice (n = 6/group). Scale bars, 200 μm for H&E, GPC3, and AFP staining; 100 μm for 4HNE staining. (F) IB analysis of tumours from above 10-mo mice (n = 3–5/group). (G) qRT-PCR analysis of tumour mRNAs from livers of above mice. Mean ± SD (n = 5–6/group). *p <0.05; **p <0.01 (Student’s *t* test). 4HNE, 4-hydroxynonenal; ALT, alanine aminotransferase; ATF4, activating transcription factor 4; AFP, alpha-foetoprotein; DEN, diethylnitrosamine; ERK1/2, extracellular signal-regulated kinase 1/2; GPC3, glypican 3; IB, immunoblotting; JNK, Jun N-terminal kinase; LFD, low-fat diet; mo, month old; Non-Tu, non-tumour; P-AKT, phosphorylated AKT; P-ERK1/2, phosphorylated ERK1/2; P-JNK, phosphorylated JNK; P-STAT3, phosphorylated STAT3; qRT-PCR, quantitative reverse-transcription PCR; STAT3, signal transducer and activator of transcription 3; Tu, tumour.

To induce HCC development, 2-wo mice were given a lower DEN dose (25 mg/kg).^5^ *Atf4*^F/F^ mice exhibited macroscopic tumours only after 10 months, whereas 5-mo *Atf4*^Δhep^ mice showed small tumours, and when 10 mo, they showed higher tumour burden than *Atf4*^F/F^ mice (Fig. 6A). Liver damage, tumour number, and size were higher in *Atf4*^Δhep^ mice (Fig. 6B and C) and so was their liver to body weight ratio (Fig. S6F). The non-tumour portion of *Atf4*^Δhep^ livers showed elevated cyclin-D1 (CCND1), mouse double minute 2 homologue (MDM2), connective tissue growth factor (CTGF), and DR5 mRNAs, but reduced FGF21 mRNA (Fig. 6D). Histological analysis showed that ATF4 ablation enhanced formation of nodular cancer foci positive for the HCC markers GPC3 and alpha-foetoprotein (AFP) (Fig. S7A). ATF4-deficient HCCs were stained more intensely for GPC3, AFP, and 4HNE (Fig. 6E). p38, STAT3, ERK1/2, and JNK phosphorylation were generally higher in ATF4-deficient tumours, which expressed more EREG but less FGF21 mRNA (Fig. 6F and G).

### Restoration of Slc7a11/xCT expression inhibits liver tumorigenesis

**Fig. 7.**
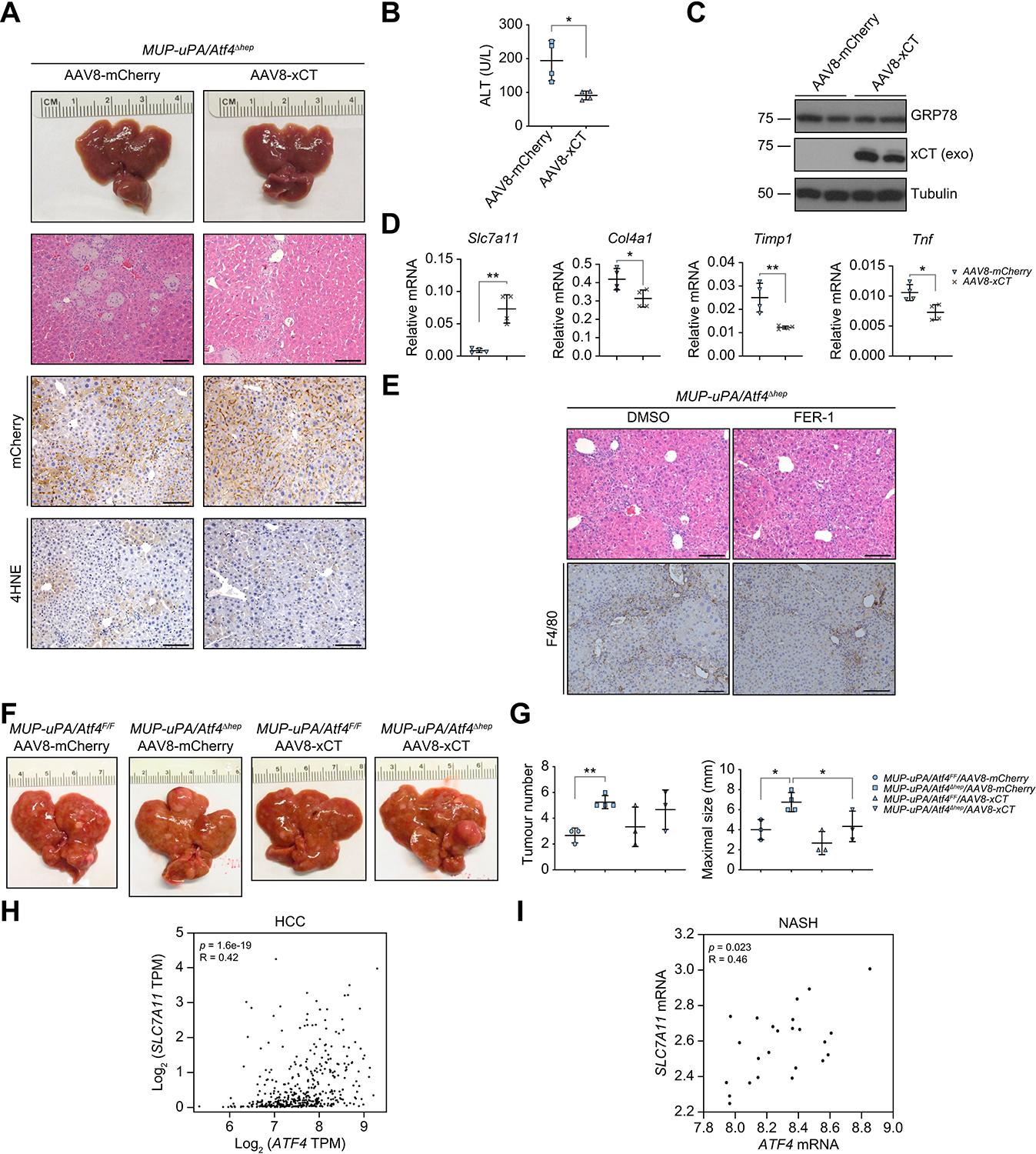
xCT reconstitution attenuates liver injury and reduces HCC burden. (A) Gross liver morphology, H&E staining, and IHC analysis of 3.5-mo LFD-fed *MUP-uPA/Atf4*^Δhep^ mice injected with AAV8-mCherry or AAV8-xCT at 8 wo (n = 4/group). Scale bars, 100 μm. (B) Serum ALT in above mice. Mean ± SD (n = 4/group). *p <0.05 (Student’s *t* test). (C) IB analysis of liver lysates from above mice. Exo indicates exogenous mCherry-xCT fusion protein. (D) qRT-PCR analysis of liver mRNAs in above mice. Mean ± SD (n = 4/group). *p <0.05; **p <0.01 (Student’s *t* test). (E) H&E and F4/80 staining of 2-mo LFD-fed *MUP-uPA/Atf4*^Δhep^ mice i.p. injected with vehicle (DMSO) or 1 mg/kg FER-1 every other day for 2 weeks (n = 3/group). Scale bars, 100 μm. (F and G) Gross liver morphology (F) and tumour burden (G) in 10-mo HFD-fed *MUP-uPA/Atf4*^F/F^ and *MUP-uPA/Atf4*^Δhep^ mice injected with AAV8-mCherry or AAV8-xCT when 8 wo. Mean ± SD (n = 3–4/group). *p <0.05; **p <0.01 (Student’s *t* test). (H and I) Correlation analysis of *ATF4* and *SLC7A11* expression. Spearman’s analysis of *ATF4* and *SLC7A1*1 mRNA amounts in human TCGA HCC datasets (H) and in patients with NASH (I) (data extracted from GSE61260). 4HNE, 4-hydroxynonenal; AAV8, adeno-associated virus 8; ALT, alanine aminotransferase; ATF4, activating transcription factor 4; FER-1, ferrostatin-1; GRP78, 78-kDa glucose-regulated protein; HCC, hepatocellular carcinoma; HFD, high-fat diet; IB, immunoblotting; IHC, immunohistochemistry; LFD, low-fat diet; MUP-uPA, major urinary protein promoter–urokinase plasminogen activator; qRT-PCR, quantitative reverse-transcription PCR; SLC7A11, solute carrier family 7a member 11; TCGA, The Cancer Genome Atlas; TPM, transcripts per million; wo, week old.

To investigate whether SLC7A11 reconstitution can prevent the enhancement of liver damage and tumour formation in ATF4-deficient mice, we used adeno-associated virus 8 (AAV8) to transduce SLC7A11, whose N-terminus was fused to mCherry (AAV8-xCT), or mCherry (AAV8-mCherry), into livers of 8-wo *MUP-uPA/Atf4*^F/F^ and *MUP-uPA/Atf4*^Δhep^ mice (Fig. S7B). We first checked liver damage in 3.5-mo LFD-fed *MUP-uPA/Atf4*^Δhep^ mice and found that AAV8-xCT-transduced *MUP-uPA/Atf4*^Δhep^ livers had smooth appearances and fewer macroscopic regenerative nodules than AAV8-mCherry-transduced livers (Fig. 7A). AAV8-mCherry and AAV8-xCT were both well expressed in the *MUP-uPA/Atf4*^Δhep^ livers. Histological analysis revealed less inflammation and lipid peroxidation in AAV-xCT-transduced livers compared with AAV8-mCherry-transduced *MUP-uPA/Atf4*^Δhep^ livers (Fig. 7A), as well as lower serum ALT (Fig. 7B). IB and qRT-PCR confirmed exogenous xCT expression in the AAV8-xCT-transduced livers (Fig. 7C and D) and lower expression of mRNAs for the fibrosis markers collagen IV α1 (COL4A1) and tissue inhibitor of metalloproteinase 1 (TIMP1), and TNF (Fig. 7D). Administration of a low dose of FER-1 every other day for 2 weeks to 6-wo *MUP-uPA/Atf4*^Δhep^ mice reduced liver inflammation (F4/80 staining) (Fig. 7E). AAV8-xCT transduction of *MUP-uPA/Atf4*^Δhep^ mice reduced liver tumour size relative to the effect of AAV8-mCherry transduction, although its effect on tumour number was not significant (Fig. 7F and G). The negligible effect on tumour initiation might be as a result of either a small sample size or delayed xCT expression or action. To overcome this limitation, we used a model of delayed HCC induction, combining DEN administration to adult mice with HFD feeding and PB administration in the drinking water.^55^ *Atf4*^F/F^ and *Atf4*^Δhep^ mice subjected to this mode of HCC induction were first injected AAV8-mCherry or AAV8-xCT when 8 wo, followed by HFD feeding when 3 mo, DEN (80 mg/kg) administration when 4 mo, and water with 0.05% PB when 5 mo, and analysed for tumour burden when 10 mo (Fig. S7B). As in young DEN-challenged mice (Fig. 6A), ATF4 ablation enhanced HCC development, and compared with AAV8-mCherry transduction, AAV8-xCT transduction significantly reduced both tumour number and tumour size (Fig. S7C and D). These results indicate that the most critical ATF4 target for suppression of liver damage, inflammation, and tumorigenesis is the small xCT subunit.

## Discussion

Normal hepatocytes barely turnover, with virtually no cell death or proliferation.^56^ However, the liver retains the ability to quickly regenerate in response to extensive hepatocellular death caused by viral, toxic, metabolic, or autoimmune insults. This so-called compensatory proliferation contributes to oncogenic transformation, and elevated circulating aminotransferases, markers for hepatocyte death, are highly predictive of HCC development in patients.^57,58^ Different forms of hepatocyte death were suggested to contribute to the cancer-promoting necroinflammatory response, including apoptosis, necrosis, and necroptosis.^11,59,60^ As shown above, a predominant driver of HCC-promoting necroinflammation in NASH-afflicted *MUP-uPA* mice is ATF4–SLC7A11-suppressible ferroptosis. Although DEN-induced liver injury is complex and involves several distinct modes of cell death, ferroptosis also appears to be a predominant cause of liver injury in mice challenged with DEN in combination with HFD and PB. SLC7A11/xCT, which determines the intracellular concentration of GSH by importing cystine and exporting glutamate at a 1:1 ratio in an ATP-dependent manner, is a key regulator of ferroptosis.^12,42^ We demonstrate that ATF4-deficient hepatocytes and livers are sensitised to ER stress-induced cell death and exhibit a substantially lower GSH:GSSG ratio and elevated lipid peroxidation, classical hallmarks of ferroptosis that can be attributed to SLC7A11 deficiency. Of note, despite being tumour suppressive in other cell types and tissues, hepatocyte cell death, which is inhibited by NF-κB, is tumour-promoting in the liver.^8^ Even in this early study, published before the discovery of ferroptosis,^12^ it was clear that the liver-damaging and HCC-promoting effect of IKKβ ablation was fully reversed by an NAC-containing diet, which restored GSH levels and prevented oxidative stress and lipid peroxidation.^8^ Likewise, in the present study, liver damage and HCC promotion were suppressed by forced SLC7A11 expression, although the hepatocytes remained ATF4 deficient. Hepatocyte-specific ablation of the antiapoptotic proteins myeloid cell leukemia-1 and B-cell lymphoma-extra large also increased the rate of apoptosis and led to spontaneous HCC development.^61,62^ It will be interesting to determine whether ferroptosis inhibitors also suppress liver damage and hepatocarcinogenesis in these mice.

Previous studies had shown that ferroptosis is mainly regulated by NRF2, which controls expression of SLC7A11 and GPX4, as well as GSH-synthesising enzymes. Ferroptosis was implicated in drug-induced liver injury (DILI), ischaemia–reperfusion injury, and chronic liver disease with one or several features of GSH depletion, iron overload, and reduced SLC7A11 and GPX4 expression.^16^ Although the role of ferroptosis in NAFLD progression and carcinogen-induced HCC has not been established, 4-HNE staining was used as an oxidative stress marker in patients with NASH.^63^ Ferroptosis, however, was shown to trigger inflammation in a mouse steatohepatitis model induced by a choline-deficient, ethionine-supplemented diet.^63^ F-box and leucine-rich repeat protein 5 (FBXL5) regulates iron homoeostasis, and FBXL5-deficient mice exhibit features of ferroptosis, including liver iron overload, oxidative stress, liver injury, and steatohepatitis, and, similar to *Atf4*^Δhep^ mice, are more susceptible to DEN-induced HCC.^63^ However, in all of these cases, it is not clear whether all hepatocytes die by ferroptosis or whether other forms of cell death, including necrosis, apoptosis, cuproptosis, and necroptosis, also take place.^11,42,60,64^ Despite these uncertainties, it is important to test whether FER-1 and other newly developed ferroptosis inhibitors can reduce liver injury and HCC susceptibility in NASH, ASH, DILI, α1 anti-trypsin deficiency, and iron overload diseases. Given that SLC7A11 is often highly expressed in different cancers, its overexpression can inhibit reactive oxygen species-induced ferroptosis, promote tumour growth, and confer drug resistance.^14,15,65,66^ ATF4 expression is also upregulated in different cancers to promote cell survival and tumour growth.^36,37^ Of note, *ATF4* and *SLC7A11* levels are positively correlated in human HCC (Fig. 7H), and ATF4-deficient HCC cells are more sensitive to ferroptosis (Fig. S7E). In human NASH specimens, *ATF4* and *SLC7A11* are also correlated (Fig. 7I), supporting our finding that inhibition of ferroptosis by the ATF4–SLC7A1 axis suppresses hepatocarcinogenesis in NASH-afflicted *MUP-uPA* mice (Fig. S7F). It is therefore plausible that ferroptosis inhibitors may be effective in preventing the progression of chronic liver diseases to HCC, although their effect on established HCC remains to be determined. The timing and duration of ferroptosis inhibition are important and should be optimised for achieving a maximal cancer preventive effect.

ATF4 is a key downstream effector of the ER and integrated stress responses, which are triggered by nutrient deprivation, viral infection, and oxidative stress.^67^ However, under persistent stress or when adaptation is insufficient, ATF4 promotes cell death.^68^ Of note, liver-specific ATF4 ablation inhibits hepatic steatosis,^30,31,33–35^ which could be a protective response that prevents further progression to steatohepatitis. Indeed, loss of ATF4 enhances liver damage.^29^ Unlike HFD-fed BL6 mice, which develop simple or benign steatosis, HFD-fed *MUP-uPA* mice progress to steatohepatitis, liver damage, and fibrosis owing to ER stress-driven hepatocyte death.^7^ The results described above suggest that ATF4 attenuates the progression from simple steatosis to NASH and HCC by suppressing ferroptosis, thereby blocking liver damage and necroinflammation. Consistent with previous publications,^30,31,34^ hepatocyte ATF4 ablation inhibited hepatic steatosis, reinforcing the notion that hepatic steatosis *per se* is not damaging and does not increase cancer risk unless it is accompanied by liver damage.

### Conclusions

Our study suggests that ferroptosis, which is inhibited by the ATF4–SLC7A11 axis, may be the most relevant form of hepatocyte death that leads to HCC-accelerating inflammation and compensatory proliferation. Ferroptosis inhibitors or ATF4 activators may be useful in preventing NASH development and its progression to HCC.

## Supplementary Material

Data 2

Data 1

Data 3

## Data Availability

The data associated with this paper and further information and requests for resources and reagents should be directed to and will be fulfilled by the lead contact, MK (karinoffice@ucsd.edu).

## Abbreviations

4HNE: 4-hydroxynonenal
β-Me: β-mercaptoethanol
AA: amino acid
AARE: AA response element
AAV8: adeno-associated virus 8
Adv: adenovirus
AFLD: alcoholic fatty liver disease
AFP: alpha-foetoprotein
ALDH7A1: alpha-aminoadipic semialdehyde dehydrogenase
ALT: alanine aminotransferase
AMT: aminomethyltransferase
ARE: antioxidant response element
ASCT2: alanine, serine, cysteine, threonine transporter 2
ASH: alcoholic steatohepatitis
ASNS: asparagine synthetase
ATF4: activating transcription factor 4
ATF6: activating transcription factor 6
BAX: Bcl-2-associated X protein
BID: BH3-interacting domain death agonist
CARS: cysteinyl-tRNA synthetase
CCL2: C–C motif chemokine ligand 2
CCND1: cyclin-D1
CHAC1: glutathione-specific gamma-glutamylcyclotransferase 1
CHOP: C/EBP-homologous protein
COL4A1: collagen IV α1
CTGF: connective tissue growth factor
CTH: cystathionine gamma-lyase
CXCL2: C-X-C motif chemokine ligand 2
CYP2E1: cytochrome P450 2E1
DEN: diethylnitrosamine
DFO: deferoxamine
DILI: drug-induced liver injury
DLK1: protein delta homologue 1
DR5: death receptor 5
EDEM2: ER degradation-enhancing alpha-mannosidase-like protein 2
eIF2α: eukaryotic translation initiation factor 2 subunit α
EPCAM: epithelial cell adhesion molecule
ER: endoplasmic reticulum
EREG: epiregulin
ERK1/2: extracellular signal-regulated kinase ½
ERP5: endoplasmic reticulum protein 5
FBXL5: F-box and leucine-rich repeat protein 5
FER-1: ferrostatin-1
FGF21: fibroblast growth factor 21
GCL: glutamate–cysteine ligase
GLUD1: glutamate dehydrogenase 1
GO: gene ontology
GPC3: glypican-3
GPX4: glutathione peroxidase 4
GRP78: 78-kDa glucose-regulated protein
GSH: glutathione
GSL2: glutaminase liver isoform
GSS: glutathione synthase
GSSG: oxidised glutathione
HCC: hepatocellular carcinoma
HFD: high-fat diet
HO1: haem oxygenase 1
IB: immunoblotting
JNK: Jun N-terminal kinase
KEGG: Kyoto Encyclopedia of Genes and Genomes
LFD: low-fat diet
MDM2: mouse double minute 2 homologue
mo: month old
MTHFD2: methenyltetrahydrofolate dehydrogenase 2
MUP: major urinary protein
NAC: N-acetyl cysteine
NAFLD: non-alcoholic fatty liver disease
NASH: non-alcoholic steatohepatitis
NEC-1: necrostatin-1
NNMT: N-nicotinamide methyltransferase
NQO1: NAD(P)H quinone dehydrogenase 1
NRF1: nuclear respiratory factor 1
NRF2: nuclear factor erythroid 2-related factor 2
P-eIF2α: phosphorylated eIF2α
PA: palmitic acid
PB: phenobarbital
PCK2: mitochondrial phosphoenolpyruvate carboxykinase
PCNA: proliferating cell nuclear antigen
PERK: pancreatic ER-regulating kinase
PHGDH: phosphoglycerate dehydrogenase
PSAT1: phosphoserine aminotransferase 1
PSPH: phosphoserine phosphatase
PTGS2: prostaglandin G/H synthase 2
PUMA: p53 upregulated modulator of apoptosis
qRT-PCR: quantitative reverse-transcription PCR
RNA-seq: RNA sequencing
RSL3: RAS-selective lethal 3
SARS: seryl-tRNA synthetase
SHMT2: serine hydroxymethyltransferase 2
SLC7A11: solute carrier family 7a member 11
SOD2: superoxide dismutase 2
STAT3: signal transducer and activator of transcription 3
T-Chol: total cholesterol
TAFLD: toxicant-associated fatty liver disease
TASH: toxicant-associated steatohepatitis
TEM: transmission electron microscopy
TFAM: mitochondrial transcription factor A
TG: triglycerides
TGFβ: transforming growth factor beta
TIMP1: tissue inhibitor of metalloproteinase 1
TM: tunicamycin
TNF: tumour necrosis factor
TNFR1: tumour necrosis factor receptor 1
TNFRSF6: tumour necrosis factor receptor superfamily member 6
TRB3: tribbles homologue 3
uPA: urokinase-type plasminogen activator
UPR: unfolded protein response
wo: week old
WT: wild-type
XBP1: X-box-binding protein 1.

## References

[1] Sung H, Ferlay J, Siegel RL, Laversanne M, Soerjomataram I, Jemal A, Global cancer statistics 2020: GLOBOCAN estimates of incidence and mortality worldwide for 36 cancers in 185 countries. CA Cancer J Clin 2021;71:209–249. 33538338 10.3322/caac.21660

[2] Anstee QM, Reeves HL, Kotsiliti E, Govaere O, Heikenwalder M. From NASH to HCC: current concepts and future challenges. Nat Rev Gastroenterol Hepatol 2019;16:411–428. 31028350 10.1038/s41575-019-0145-7

[3] Kim JY, He F, Karin M. From liver fat to cancer: perils of the Western diet. Cancers 2021;13:1095. 33806428 10.3390/cancers13051095 PMC7961422

[4] Llovet JM, Montal R, Sia D, Finn RS. Molecular therapies and precision medicine for hepatocellular carcinoma. Nat Rev Clin Oncol 2018;15:599–616. 30061739 10.1038/s41571-018-0073-4 PMC12452113

[5] Umemura A, He F, Taniguchi K, Nakagawa H, Yamachika S, Font-Burgada J, p62, upregulated during preneoplasia, induces hepatocellular carcinogenesis by maintaining survival of stressed HCC-initiating cells. Cancer Cell 2016;29:935–948. 27211490 10.1016/j.ccell.2016.04.006 PMC4907799

[6] Loomba R, Friedman SL, Shulman GI. Mechanisms and disease consequences of nonalcoholic fatty liver disease. Cell 2021;184:2537–2564. 33989548 10.1016/j.cell.2021.04.015 PMC12168897

[7] Nakagawa H, Umemura A, Taniguchi K, Font-Burgada J, Dhar D, Ogata H, ER stress cooperates with hypernutrition to trigger TNF-dependent spontaneous HCC development. Cancer Cell 2014;26:331–343. 25132496 10.1016/j.ccr.2014.07.001 PMC4165611

[8] Maeda S, Kamata H, Luo JL, Leffert H, Karin M. IKKb couples hepatocyte death to cytokine-driven compensatory proliferation that promotes chemical hepatocarcinogenesis. Cell 2005;121:977–990. 15989949 10.1016/j.cell.2005.04.014

[9] Schneider AT, Gautheron J, Feoktistova M, Roderburg C, Loosen SH, Roy S, RIPK1 suppresses a TRAF2-dependent pathway to liver cancer. Cancer Cell 2017;31:94–109. 28017612 10.1016/j.ccell.2016.11.009

[10] Schwabe RF, Luedde T. Apoptosis and necroptosis in the liver: a matter of life and death. Nat Rev Gastroenterol Hepatol 2018;15:738–752. 30250076 10.1038/s41575-018-0065-y PMC6490680

[11] Luedde T, Kaplowitz N, Schwabe RF. Cell death and cell death responses in liver disease: mechanisms and clinical relevance. Gastroenterology 2014;147:765–783.e4. 25046161 10.1053/j.gastro.2014.07.018 PMC4531834

[12] Dixon SJ, Lemberg KM, Lamprecht MR, Skouta R, Zaitsev EM, Gleason CE, Ferroptosis: an iron-dependent form of nonapoptotic cell death. Cell 2012;149:1060–1072. 22632970 10.1016/j.cell.2012.03.042 PMC3367386

[13] Zhong Z, Umemura A, Sanchez-Lopez E, Liang S, Shalapour S, Wong J, NF-jB restricts inflammasome activation via elimination of damaged mitochondria. Cell 2016;164:896–910. 26919428 10.1016/j.cell.2015.12.057 PMC4769378

[14] Jiang L, Kon N, Li T, Wang SJ, Su T, Hibshoosh H, Ferroptosis as a p53-mediated activity during tumour suppression. Nature 2015;520:57–62. 25799988 10.1038/nature14344 PMC4455927

[15] Hu K, Li K, Lv J, Feng J, Chen J, Wu H, Suppression of the SLC7A11/glutathione axis causes synthetic lethality in KRAS-mutant lung adenocarcinoma. J Clin Invest 2020;130:1752–1766. 31874110 10.1172/JCI124049 PMC7108883

[16] Wang H, An P, Xie E, Wu Q, Fang X, Gao H, Characterization of ferroptosis in murine models of hemochromatosis. Hepatology 2017;66:449–465. 28195347 10.1002/hep.29117 PMC5573904

[17] Hetz C, Zhang K, Kaufman RJ. Mechanisms, regulation and functions of the unfolded protein response. Nat Rev Mol Cel Biol 2020;21:421–438. 10.1038/s41580-020-0250-z PMC8867924 32457508

[18] Wang M, Kaufman RJ. The impact of the endoplasmic reticulum protein-folding environment on cancer development. Nat Rev Cancer 2014;14:581–597. 25145482 10.1038/nrc3800

[19] Wu Y, Shan B, Dai J, Xia Z, Cai J, Chen T, Dual role for inositol-requiring enzyme 1ɑ in promoting the development of hepatocellular carcinoma during diet-induced obesity in mice. Hepatology 2018;68:533–546. 29506314 10.1002/hep.29871

[20] Zhang K, Wang S, Malhotra J, Hassler JR, Back SH, Wang G, The unfolded protein response transducer IRE1ɑ prevents ER stress-induced hepatic steatosis. EMBO J 2011;30:1357–1375. 21407177 10.1038/emboj.2011.52 PMC3094110

[21] Wang JM, Qiu Y, Yang Z, Kim H, Qian Q, Sun Q, IRE1ɑ prevents hepatic steatosis by processing and promoting the degradation of select microRNAs. Sci Signal 2018;11:eaao4617. 10.1126/scisignal.aao4617 PMC6075656 29764990

[22] Niederreiter L, Fritz TM, Adolph TE, Krismer AM, Offner FA, Tschurtschenthaler M, ER stress transcription factor Xbp1 suppresses intestinal tumorigenesis and directs intestinal stem cells. J Exp Med 2013;210:2041–2056. 24043762 10.1084/jem.20122341 PMC3782039

[23] Liu X, Henkel AS, LeCuyer BE, Schipma MJ, Anderson KA, Green RM. Hepatocyte X-box binding protein 1 deficiency increases liver injury in mice fed a high-fat/sugar diet. Am J Physiol Gastrointest Liver Physiol 2015;309:G965–G974. 26472223 10.1152/ajpgi.00132.2015 PMC4683298

[24] Arai M, Kondoh N, Imazeki N, Hada A, Hatsuse K, Kimura F, Transformation-associated gene regulation by ATF6ɑ during hepatocarcinogenesis. FEBS Lett 2006;580:184–190. 16364319 10.1016/j.febslet.2005.11.072

[25] Hart LS, Cunningham JT, Datta T, Dey S, Tameire F, Lehman SL, ER stress-mediated autophagy promotes Myc-dependent transformation and tumor growth. J Clin Invest 2012;122:4621–4634. 23143306 10.1172/JCI62973 PMC3533536

[26] Harding HP, Zhang Y, Zeng H, Novoa I, Lu PD, Calfon M, An integrated stress response regulates amino acid metabolism and resistance to oxidative stress. Mol Cel 2003;11:619–633. 10.1016/s1097-2765(03)00105-9 12667446

[27] Seo J, Fortuno ES 3rd, Suh JM, Stenesen D, Tang W, Parks EJ, Atf4 regulates obesity, glucose homeostasis, and energy expenditure. Diabetes 2009;58:2565–2573. 19690063 10.2337/db09-0335 PMC2768187

[28] Wang C, Huang Z, Du Y, Cheng Y, Chen S, Guo F. ATF4 regulates lipid metabolism and thermogenesis. Cell Res 2010;20:174–184. 20066008 10.1038/cr.2010.4

[29] Fusakio ME, Willy JA, Wang Y, Mirek ET, Al Baghdadi RJ, Adams CM, Transcription factor ATF4 directs basal and stress-induced gene expression in the unfolded protein response and cholesterol metabolism in the liver. Mol Biol Cel 2016;27:1536–1551. 10.1091/mbc.E16-01-0039 PMC4850040 26960794

[30] Xiao G, Zhang T, Yu S, Lee S, Calabuig-Navarro V, Yamauchi J, ATF4 protein deficiency protects against high fructose-induced hyper-triglyceridemia in mice. J Biol Chem 2013;288:25350–25361. 23888053 10.1074/jbc.M113.470526 PMC3757199

[31] Li H, Meng Q, Xiao F, Chen S, Du Y, Yu J, ATF4 deficiency protects mice from high-carbohydrate-diet-induced liver steatosis. Biochem J 2011;438:283–289. 21644928 10.1042/BJ20110263

[32] Yueh MF, He F, Chen C, Vu C, Tripathi A, Knight R, Triclosan leads to dysregulation of the metabolic regulator FGF21 exacerbating high fat diet-induced nonalcoholic fatty liver disease. Proc Natl Acad Sci U S A 2020;117:31259–31266. 33229553 10.1073/pnas.2017129117 PMC7733785

[33] Song Q, Chen Y, Wang J, Hao L, Huang C, Griffiths A, ER stress-induced upregulation of NNMT contributes to alcohol-related fatty liver development. J Hepatol 2020;73:783–793. 32389809 10.1016/j.jhep.2020.04.038 PMC8301603

[34] Li K, Xiao Y, Yu J, Xia T, Liu B, Guo Y, Liver-specific gene inactivation of the transcription factor ATF4 alleviates alcoholic liver steatosis in mice. J Biol Chem 2016;291:18536–18546. 27405764 10.1074/jbc.M116.726836 PMC5000098

[35] Hao L, Zhong W, Dong H, Guo W, Sun X, Zhang W, ATF4 activation promotes hepatic mitochondrial dysfunction by repressing NRF1-TFAM signalling in alcoholic steatohepatitis. Gut 2021;70:1933–1945. 33177163 10.1136/gutjnl-2020-321548 PMC8110597

[36] Ye J, Kumanova M, Hart LS, Sloane K, Zhang H, De Panis DN, The GCN2-ATF4 pathway is critical for tumour cell survival and proliferation in response to nutrient deprivation. EMBO J 2010;29:2082–2096. 20473272 10.1038/emboj.2010.81 PMC2892366

[37] Tameire F, Verginadis II, Leli NM, Polte C, Conn CS, Ojha R, ATF4 couples MYC-dependent translational activity to bioenergetic demands during tumour progression. Nat Cel Biol 2019;21:889–899. 10.1038/s41556-019-0347-9 PMC6608727 31263264

[38] Ebert SM, Dyle MC, Kunkel SD, Bullard SA, Bongers KS, Fox DK, Stress-induced skeletal muscle Gadd45a expression reprograms myonuclei and causes muscle atrophy. J Biol Chem 2012;287:27290–27301. 22692209 10.1074/jbc.M112.374777 PMC3431665

[39] Chan K, Lu R, Chang JC, Kan YW. NRF2, a member of the NFE2 family of transcription factors, is not essential for murine erythropoiesis, growth, and development. Proc Natl Acad Sci U S A 1996;93:13943–13948. 8943040 10.1073/pnas.93.24.13943 PMC19474

[40] Sandgren EP, Palmiter RD, Heckel JL, Daugherty CC, Brinster RL, Degen JL. Complete hepatic regeneration after somatic deletion of an albumin-plasminogen activator transgene. Cell 1991;66:245–256. 1713128 10.1016/0092-8674(91)90615-6

[41] He F, Antonucci L, Yamachika S, Zhang Z, Taniguchi K, Umemura A, NRF2 activates growth factor genes and downstream AKT signaling to induce mouse and human hepatomegaly. J Hepatol 2020;72:1182–1195. 32105670 10.1016/j.jhep.2020.01.023 PMC8054878

[42] Stockwell BR. Ferroptosis turns 10: emerging mechanisms, physiological functions, and therapeutic applications. Cell 2022;185:2401–2421. 35803244 10.1016/j.cell.2022.06.003 PMC9273022

[43] Cao SS, Kaufman RJ. Endoplasmic reticulum stress and oxidative stress in cell fate decision and human disease. Antioxid Redox Signal 2014;21:396–413. 24702237 10.1089/ars.2014.5851 PMC4076992

[44] Dixon SJ, Patel DN, Welsch M, Skouta R, Lee ED, Hayano M, Pharmacological inhibition of cystine–glutamate exchange induces endoplasmic reticulum stress and ferroptosis. Elife 2014;3:e02523. 24844246 10.7554/eLife.02523 PMC4054777

[45] Shin D, Kim EH, Lee J, Roh JL. Nrf2 inhibition reverses resistance to GPX4 inhibitor-induced ferroptosis in head and neck cancer. Free Radic Biol Med 2018;129:454–462. 30339884 10.1016/j.freeradbiomed.2018.10.426

[46] Yamamoto M, Kensler TW, Motohashi H. The KEAP1-NRF2 system: a thiol-based sensor-effector apparatus for maintaining redox homeostasis. Physiol Rev 2018;98:1169–1203. 29717933 10.1152/physrev.00023.2017 PMC9762786

[47] Ma Y, Brewer JW, Diehl JA, Hendershot LM. Two distinct stress signaling pathways converge upon the CHOP promoter during the mammalian unfolded protein response. J Mol Biol 2002;318:1351–1365. 12083523 10.1016/s0022-2836(02)00234-6

[48] Zong ZH, Du ZX, Li N, Li C, Zhang Q, Liu BQ, Implication of Nrf2 and ATF4 in differential induction of CHOP by proteasome inhibition in thyroid cancer cells. Biochim Biophys Acta 2012;1823:1395–1404. 22691366 10.1016/j.bbamcr.2012.06.001

[49] He CH, Gong P, Hu B, Stewart D, Choi ME, Choi AM, Identification of activating transcription factor 4 (ATF4) as an Nrf2-interacting protein. Implication for heme oxygenase-1 gene regulation. J Biol Chem 2001;276:20858–20865. 11274184 10.1074/jbc.M101198200

[50] Sasaki H, Sato H, Kuriyama-Matsumura K, Sato K, Maebara K, Wang H, Electrophile response element-mediated induction of the cystine/glutamate exchange transporter gene expression. J Biol Chem 2002;277:44765–44771. 12235164 10.1074/jbc.M208704200

[51] Ye P, Mimura J, Okada T, Sato H, Liu T, Maruyama A, Nrf2- and ATF4-dependent upregulation of xCT modulates the sensitivity of T24 bladder carcinoma cells to proteasome inhibition. Mol Cel Biol 2014;34:3421–3434. 10.1128/MCB.00221-14 PMC4135628 25002527

[52] Asgharpour A, Cazanave SC, Pacana T, Seneshaw M, Vincent R, Banini BA, A diet-induced animal model of non-alcoholic fatty liver disease and hepatocellular cancer. J Hepatol 2016;65:579–588. 27261415 10.1016/j.jhep.2016.05.005 PMC5012902

[53] Vandewynckel YP, Laukens D, Bogaerts E, Paridaens A, Van den Bussche A, Verhelst X, Modulation of the unfolded protein response impedes tumor cell adaptation to proteotoxic stress: a PERK for hepatocellular carcinoma therapy. Hepatol Int 2015;9:93–104. 25598862 10.1007/s12072-014-9582-0 PMC4289530

[54] Halpern KB, Shenhav R, Matcovitch-Natan O, Toth B, Lemze D, Golan M, Single-cell spatial reconstruction reveals global division of labour in the mammalian liver. Nature 2017;542:352–356. 28166538 10.1038/nature21065 PMC5321580

[55] Park EJ, Lee JH, Yu GY, He G, Ali SR, Holzer RG, Dietary and genetic obesity promote liver inflammation and tumorigenesis by enhancing IL-6 and TNF expression. Cell 2010;140:197–208. 20141834 10.1016/j.cell.2009.12.052 PMC2836922

[56] Benedetti A, Jézéquel AM, Orlandi F. Preferential distribution of apoptotic bodies in acinar zone 3 of normal human and rat liver. J Hepatol 1988;7:319–324. 3235800 10.1016/s0168-8278(88)80004-7

[57] Yang HI, Yuen MF, Chan HL, Han KH, Chen PJ, Kim DY, Risk estimation for hepatocellular carcinoma in chronic hepatitis B (REACH-B): development and validation of a predictive score. Lancet Oncol 2011;12:568–574. 21497551 10.1016/S1470-2045(11)70077-8

[58] Wen CP, Lin J, Yang YC, Tsai MK, Tsao CK, Etzel C, Hepatocellular carcinoma risk prediction model for the general population: the predictive power of transaminases. J Natl Cancer Inst 2012;104:1599–1611. 23073549 10.1093/jnci/djs372 PMC3692381

[59] Seehawer M, Heinzmann F, D’Artista L, Harbig J, Roux PF, Hoenicke L, Necroptosis microenvironment directs lineage commitment in liver cancer. Nature 2018;562:69–75. 30209397 10.1038/s41586-018-0519-y PMC8111790

[60] Nanji AA, Hiller-Sturmhöfel S. Apoptosis and necrosis: two types of cell death in alcoholic liver disease. Alcohol Health Res World 1997;21:325–330. 15706744 PMC6827678

[61] Weber A, Boger R, Vick B, Urbanik T, Haybaeck J, Zoller S, Hepatocyte-specific deletion of the antiapoptotic protein myeloid cell leukemia-1 triggers proliferation and hepatocarcinogenesis in mice. Hepatology 2010;51:1226–1236. 20099303 10.1002/hep.23479 PMC2936921

[62] Hikita H, Kodama T, Shimizu S, Li W, Shigekawa M, Tanaka S, Bak deficiency inhibits liver carcinogenesis: a causal link between apoptosis and carcinogenesis. J Hepatol 2012;57:92–100. 22414765 10.1016/j.jhep.2012.01.027

[63] Tsurusaki S, Tsuchiya Y, Koumura T, Nakasone M, Sakamoto T, Matsuoka M, Hepatic ferroptosis plays an important role as the trigger for initiating inflammation in nonalcoholic steatohepatitis. Cell Death Dis 2019;10:449. 31209199 10.1038/s41419-019-1678-y PMC6579767

[64] Tsvetkov P, Coy S, Petrova B, Dreishpoon M, Verma A, Abdusamad M, Copper induces cell death by targeting lipoylated TCA cycle proteins. Science 2022;375:1254–1261. 35298263 10.1126/science.abf0529 PMC9273333

[65] Gao R, Kalathur RKR, Coto-Llerena M, Ercan C, Buechel D, Shuang S, YAP/TAZ and ATF4 drive resistance to Sorafenib in hepatocellular carcinoma by preventing ferroptosis. EMBO Mol Med 2021;13:e14351. 34664408 10.15252/emmm.202114351 PMC8649869

[66] Sun X, Ou Z, Chen R, Niu X, Chen D, Kang R, Activation of the p62-Keap1-NRF2 pathway protects against ferroptosis in hepatocellular carcinoma cells. Hepatology 2016;63:173–184. 26403645 10.1002/hep.28251 PMC4688087

[67] Costa-Mattioli M, Walter P. The integrated stress response: from mechanism to disease. Science 2020;368:eaat5314. 32327570 10.1126/science.aat5314 PMC8997189

[68] Han J, Back SH, Hur J, Lin YH, Gildersleeve R, Shan J, ER-stress-induced transcriptional regulation increases protein synthesis leading to cell death. Nat Cel Biol 2013;15:481–490. 10.1038/ncb2738 PMC3692270 23624402

