# Supplementary material for: ATF4 suppresses hepatocarcinogenesis by inducing SLC7A11 (xCT) to block stress-related ferroptosis
Source: J Hepatol. Author manuscript; Available in PMC 2024 Aug 19. (PMC11332364; doi:10.1016/j.jhep.2023.03.016)
Supplement: Data 2 [file NIHMS2013638-supplement-Data_2.docx]

**Journal of Hepatology**

**CTAT methods**

Tables for a “Complete, Transparent, Accurate and Timely account” (CTAT) are now mandatory for all revised submissions. The aim is to enhance the reproducibility of methods.

- Only include the parts relevant to your study
- Refer to the CTAT in the main text as ‘Supplementary CTAT Table’
- Do not add subheadings
- Add as many rows as needed to include all information
- Only include one item per row

**If the CTAT form is not relevant to your study, please outline the reasons why:**

|  |
| --- |

- 1. **Antibodies**

| **Name** | **Citation** | **Supplier** | **Cat no.** | **Clone no.** |
| --- | --- | --- | --- | --- |
| Guinea pig anti-mouse/human p62/SQSTM1 (C-terminus) | RRID:AB_2687531 | Progen | GP62-C | NA |
| Mouse monoclonal anti-human p62/SQSTM1 | RRID:AB_2800125 | Cell Signaling Technologies | 88588 | D5L7G |
| Mouse monoclonal anti-mCherry | RRID:AB_11133266 | Abcam | ab125096 | 1C51 |
| Rabbit monoclonal anti-NQO1 | RRID:AB_2799623 | Cell Signaling Technologies | 62262 | D6H3A |
| Rabbit monoclonal anti-NQO1 | RRID:AB_2154354 | Cell Signaling Technologies | 3187 | A180 |
| Rabbit polyclonal anti-mouse/human p44/42 MAPK (Erk1/2) | RRID: AB_330744 | Cell Signaling Technologies | 9102 | NA |
| Rabbit monoclonal anti-ATF4 (D4B8) | RRID:AB_2616025 | Cell Signaling Technologies | 11815 | D4B8 |
| Rabbit polyclonal anti-ATF4 | RRID:AB_2058600 | Proteintech | 10835-1-AP | NA |
| Rabbit polyclonal anti-Phospho-p44/42MAPK (Erk1/2) (Thr202/ Tyr204) | RRID: AB_331646 | Cell Signaling Technologies | 9101 | NA |
| Rabbit monoclonal anti-mouse/human Ki67 | RRID: AB_422351 | GeneTex | GTX16667 | SP6 |
| Rabbit monoclonal anti-NRF2 (D1Z9C) XP | RRID:AB_2715528 | Cell Signaling Technologies | 12721 | D1Z9C |
| Rabbit polyclonal anti-Phospho-Stat3 (Tyr705) Antibody #9131 | RRID:AB_331586 | Cell Signaling Technologies | 9131 | NA |
| Rabbit monoclonal anti- Stat3 (D3Z2G) antibody | RRID: AB_2629499 | Cell Signaling Technologies | 12640 | D3Z2G |
| Rabbit monoclonal anti- Phospho-eIF2α (Ser51) (119A11) | RRID: AB_390740 | Cell Signaling Technologies | 3597 | 119A11 |
| Rabbit monoclonal anti- eIF2alpha (D7D3) XP | RRID:AB_10692650 | Cell Signaling Technologies | 5324 | D7D3 |
| Rabbit Polyclonal HDAC1 (H-51) antibody | RRID: AB_2279709 | Santa Cruz Biotechnology | 7872 | NA |
| Rabbit Polyclonal anti- Phospho-SAPK/JNK (Thr183/Tyr185) Antibody | RRID:AB_331659 | Cell Signaling Technologies | 9251 | NA |
| Mouse monoclonal anti-PCNA | RRID:AB_397992 | BD Biosciences | 610665 | 24 |
| Mouse monoclonal anti- JNK1/JNK2 antibody | RRID:AB_395344 | BD Biosciences | 554285 | G151-666 |
| Rabbit Polyclonal anti-GRP78 (H-129) antibody | RRID: AB_2119991 | Santa Cruz Biotechnology | 13968 | NA |
| Mouse monoclonal anti-ERp5 | RRID:AB_10843798 | Santa Cruz Biotechnology | 365260 | G5 |
| Mouse monoclonal anti-CHOP (L63F7) | RRID: AB_2089254 | Cell Signaling Technologies | 2895 | L63F6 |
| Rabbit polyclonal anti-Phospho-Akt (Ser473) | RRID:AB_329825 | Cell Signaling Technologies | 9271 | NA |
| Rabbit monoclonal anti-Phospho-Akt (Ser473) (D9E) | RRID:AB_2315049 | Cell Signaling Technologies | 4060 | D9E |
| Mouse monoclonal anti-alpha-Tubulin | RRID:AB_477579 | Sigma-Aldrich | T-5168 | B-5-1-2 |
| Mouse monoclonal anti-Akt1/2/3 | RRID:AB_1118808 | Santa Cruz Biotechnology | 81434 | 5C10 |
| Rabbit Polyclonal anti- Phospho-p38 MAPK (Thr180/Tyr182) Antibody | RRID:AB_331641 | Cell Signaling Technologies | 9211 | NA |
| Rabbit Polyclonal anti-xCT | RRID:AB_2800296 | Cell Signaling Technologies | 98051 | NA |
| Rabbit monoclonal anti- Phospho-Histone H2A.X (Ser139) | RRID:AB_2118009 | Cell Signaling Technologies | 9718 | 20E3 |
| Rabbit Polyclonal anti-p53 | RRID:AB_563933 | Leica Biosystems | NCL-p53-CM5p | NA |
| Rabbit Polyclonal anti-P38 alpha | RRID:AB_632138 | Santa Cruz Biotechnology | 435 | NA |
| Rabbit monoclonal anti-Akt (Pan) (C67E7) | RRID:AB_915783 | Cell Signaling Technologies | 4691 | C67E7 |
| Mouse monoclonal anti- Actin | RRID:AB_476730 | Sigma-Aldrich | A4700 | AC-40 |
| Horse anti-mouse IgG, HRP-linked Antibody | RRID:AB_330924 | Cell Signaling Technologies | 7076 | NA |
| Goat anti-rabbit IgG, HRP-linked Antibody | RRID:AB_2099233 | Cell Signaling Technologies | 7074 | NA |
| Rabbit Polyclonal anti-Glypican 3 | RRID:AB_1141042 | Abcam | ab66596 | NA |
| Mouse Monoclonal Anti-4-Hydroxy-2-Nonenal Antibody | RRID:AB_867452 | Abcam | ab48506 | HNEJ-2 |
| Goat Polyclonal anti-Mouse alpha-Fetoprotein | RRID: AB_2258018 | R&D Systems | AF5369 | NA |
| Rat Monoclonal anti-F4/80 | RRID:AB_2277854 | Thermo Fisher | MA1-91124 | A3-1 |
| Rabbit Polyclonal anti- Cytochrome P450 Enzyme CYP2E1 | RRID:AB_11212002 | Millipore | AB1252 | NA |
| Rabbit Polyclonal anti-TRB3 | RRID:AB_2200966 | Millipore | ST1032 | NA |
| Goat anti-Guinea Pig IgG, HRP-linked Antibody | RRID:AB_2535546 | Thermo Fisher Scientific | A18769 | NA |
| Biotin Goat anti-Mouse Ig (IHC) | RRID: AB_395196 | BD Pharmingen | 553999 | NA |
| Biotin Goat anti-Rabbit Ig (IHC) | RRID: AB_393618 | BD Pharmingen | 550338 | NA |

- 1. **Cell lines**

| **Name** | **Citation** | **Supplier** | **Cat no.** | **Passage no.** | **Authentication test method** |
| --- | --- | --- | --- | --- | --- |
| HEK293 cells |  | ATCC | Cat# CRL-1573 | 7 | QPCR |
| 293A |  | Thermo Fisher Scientific | Cat#R70507 | 6 | QPCR |

- 1. **Organisms**

| **Name** | **Citation** | **Supplier** | **Strain** | **Sex** | **Age** | **Overall n number** |
| --- | --- | --- | --- | --- | --- | --- |
| Mouse: C57BL/6 | NA | Charles river | B6, code:  027 | Male | 1.5-4 months | 10 |
| Mouse:  *Atf4^F/F^ and*  *Atf4^Δhep^* | J Biol Chem 2012;287:27290-27301 and in this paper | Christopher Adams | B6 | Male/Female | 0.5 -10 months | 39 and 44 |
| Mouse: B6.129X1-Nfe2l2tm1Ywk/J | J Hepatol 2020;72(6):1182-95 | The Jackson Laboratory | B6, code: 017009 | male | 2-4 months | 9 |
| Mouse:  *MUP-uPA* | Am. J. Pathol. 2000;157:1963-1974 | Eric P. Sandgren | B6 | male | 1-10 months | 10 |
| Mouse: Albumin-Cre | J Hepatol 2020;72(6):1182-95 | The Jackson Laboratory | Stock# 003574 | Male/Female | 2-4 months | Crossed with floxed mice |
| Mous*e: MUP-uPA/Atf4^F/F^* and *MUP-uPA/Atf4^Δhep^* | Established in this paper | Homemade | B6 | Male/Female | 1.5-10months | 22 and 39 |

- 1. **Sequence based reagents**

| **Name** | **Sequence** | **Supplier** |
| --- | --- | --- |
| Mouse *Hprt1_*Forward | ACCTGGTTCATCATCGCTAA | INTEGRATED DNA TECHNOLOGIES |
| Mouse *Hprt1_*Reverse | CTCCTCAGACCGCTTTTTG | INTEGRATED DNA TECHNOLOGIES |
| Mouse *Nqo1*_Forward | AGCGTTCGGTATTACGAT CC | INTEGRATED DNA TECHNOLOGIES |
| Mouse *Nqo1*_Reverse | AGTACAATCAGGGCTCTTCTCG | INTEGRATED DNA TECHNOLOGIES |
| Mouse *Atf4_*Forward | TGACCGAGATGAGCTTCCTGA | INTEGRATED DNA TECHNOLOGIES |
| Mouse *Atf4_* Reverse: | GAGAACCCATGAGGTTTCAAGTG | INTEGRATED DNA TECHNOLOGIES |
| Mouse *Erp5*_Forward | TCACTAGAAGAATACAGACCGCTG | INTEGRATED DNA TECHNOLOGIES |
| Mouse *Erp5*_Reverse | GGACAGAATGCGGGTGAT | INTEGRATED DNA TECHNOLOGIES |
| Mouse *Grp78/Bip*_Forward | GGTGCAGCAGGACATCAAGTT | INTEGRATED DNA TECHNOLOGIES |
| Mouse *Grp78/bIP*_Reverse | CCCACCTCC AATATCAACTTG A | INTEGRATED DNA TECHNOLOGIES |
| Mouse *Mdm2*_Forward | GCGTGGAATTTGAAGTTGAGTC | INTEGRATED DNA TECHNOLOGIES |
| Mouse *Mdm2*_Reverse | CTGTATCGCTTTCTCCTGTCTG | INTEGRATED DNA TECHNOLOGIES |
| Mouse *Chop_*Forward | CTGCCTTTCACCTTGGAGAC | INTEGRATED DNA TECHNOLOGIES |
| Mouse *Chop_*Reverse | CGTTTCCTGGGGATGAGATA | INTEGRATED DNA TECHNOLOGIES |
| Mouse *Edem2_*Forward | CATTGTCCTGAAGAACCTCCA | INTEGRATED DNA TECHNOLOGIES |
| Mouse *Edem2 _*Reverse | ACCTGGGGCAGTTTTTCTCT | INTEGRATED DNA TECHNOLOGIES |
| Mouse *Tnf_*Forward | AGGGTCTGGGCCATAGAACT | INTEGRATED DNA TECHNOLOGIES |
| Mouse *Tnf_*Reverse | CCACCACGCTCTTCTGTCTAC | INTEGRATED DNA TECHNOLOGIES |
| Mouse *F4/80_*Forward | GGATGTACAGATGGGGGATG | INTEGRATED DNA TECHNOLOGIES |
| Mouse *F4/80 _*Reverse | GTCTGTGGTGTCAGTGCAGG | INTEGRATED DNA TECHNOLOGIES |
| Mouse *Il1a_*Forward | ATGTATGCCTACTCGTCGGG | INTEGRATED DNA TECHNOLOGIES |
| Mouse *Il1a_*Reverse | TGAGTTTTGGTGTTTCTGGC | INTEGRATED DNA TECHNOLOGIES |
| Mouse *Mthfd2_*Forward | ACAGATGGAGCTCACGAACG | INTEGRATED DNA TECHNOLOGIES |
| Mouse *Mthfd2_*Reverse | TGCCAGCGGCAGATATTACA | INTEGRATED DNA TECHNOLOGIES |
| Mouse *Gls2_*Forward | AGCGTATCCCTATCCACAAGTTCA | INTEGRATED DNA TECHNOLOGIES |
| Mouse *Gls2_*Reverse | GCAGTCCAGTGGCCTTCAGAG | INTEGRATED DNA TECHNOLOGIES |
| Mouse *Glud1_*Forward | AGCCAGTGCTTTTACTTCATCC | INTEGRATED DNA TECHNOLOGIES |
| Mouse *Glud1_*Reverse | GGGAGGTCATCGAAGGCTAC | INTEGRATED DNA TECHNOLOGIES |
| Mouse *Phgdh_*Forward | GCACACCTTTCTTGCACTGA | INTEGRATED DNA TECHNOLOGIES |
| Mouse *Phgdh_*Reverse | GGAGGAGATCTGGCCTCTCT | INTEGRATED DNA TECHNOLOGIES |
| Mouse *Psat1_*Forward | TTGATCCATTCCAGGACCAT | INTEGRATED DNA TECHNOLOGIES |
| Mouse *Psat1 _*Reverse | ACTACAAAGTGCAGGCTGGG | INTEGRATED DNA TECHNOLOGIES |
| Mouse *Chac1_*Forward | AGGTACTTCAGGGCCTCGTT | INTEGRATED DNA TECHNOLOGIES |
| Mouse *Chac1 _*Reverse | GTGGTGACCCTCCTTGAAGA | INTEGRATED DNA TECHNOLOGIES |
| Mouse *Pck2_*Forward | AGTTTGGATGTGCACAGGGT | INTEGRATED DNA TECHNOLOGIES |
| Mouse *Pck2 _*Reverse | GTACTGGGAAGGCATTGACC | INTEGRATED DNA TECHNOLOGIES |
| Mouse *Psph_*Forward | TCTGAGTGGGAGACCATCCT | INTEGRATED DNA TECHNOLOGIES |
| Mouse *Psph _*Reverse | CCCACTGCTACCGGAAAGT | INTEGRATED DNA TECHNOLOGIES |
| Mouse *Fgf21_*Forward | CTCCAGCAGCAGTTCTCTGA | INTEGRATED DNA TECHNOLOGIES |
| Mouse *Fgf21 _*Reverse | CCTGGGTGTCAAAGCCTCTA | INTEGRATED DNA TECHNOLOGIES |
| Mouse *Cars_*Forward | GCCAGTCAGAGAGCAAGTCC | INTEGRATED DNA TECHNOLOGIES |
| Mouse *Cars _*Reverse | CAGAACCACTGGAACAGGCT | INTEGRATED DNA TECHNOLOGIES |
| Mouse *Asns_*Forward | TCACCATCCACATTGGTCTG | INTEGRATED DNA TECHNOLOGIES |
| Mouse *Asns _*Reverse | TTTGTGGCTCTGTTACAATGG | INTEGRATED DNA TECHNOLOGIES |
| Mouse *Slc1a5_*Forward | CTCATGTAAAATACCGCAATCCTGT | INTEGRATED DNA TECHNOLOGIES |
| Mouse *Slc1a5 _*Reverse | TCATTCCCTCCACCTCACAGA | INTEGRATED DNA TECHNOLOGIES |
| Mouse *Slc7a5_*Forward | CTGGTCTTCGCCACCTACTT | INTEGRATED DNA TECHNOLOGIES |
| Mouse *Slc7a5 _*Reverse | GCCTTTACGCTGTAGCAGTTC | INTEGRATED DNA TECHNOLOGIES |
| Mouse *Ptgs2_*Forward | CTGCGCCTTTTCAAGGATGG | INTEGRATED DNA TECHNOLOGIES |
| Mouse *Ptgs2 _*Reverse | GGGGATACACCTCTCCACCA | INTEGRATED DNA TECHNOLOGIES |
| Mouse *Slc1a4_*Forward | CAGAAACCTGTTCCCTTCCA | INTEGRATED DNA TECHNOLOGIES |
| Mouse *Slc1a4 _*Reverse | ACGGGGATCTTCTCTTTGGT | INTEGRATED DNA TECHNOLOGIES |
| Mouse *Gss_*Forward | GTGAATGGGGCATACGTCA | INTEGRATED DNA TECHNOLOGIES |
| Mouse *Gss_*Reverse | CAAAGCAGGCCATAGACAGG | INTEGRATED DNA TECHNOLOGIES |
| Mouse *Ho1_*Forward | CCTTCAAGGCCTCAGACAAA | INTEGRATED DNA TECHNOLOGIES |
| Mouse *Ho1_*Reverse | GAGCCTGAATCGAGCAGAAC | INTEGRATED DNA TECHNOLOGIES |
| Mouse *Gclm_*Forward | TTGGGAACTCCATTCATTCA | INTEGRATED DNA TECHNOLOGIES |
| Mouse *Gclm _*Reverse | CGGGAACCTGCTCAACTG | INTEGRATED DNA TECHNOLOGIES |
| Mouse *Slc7a11*_ Forward | TCTGGTCTGCCTGTGGAGTA | INTEGRATED DNA TECHNOLOGIES |
| Mouse *Slc7a11*_ Reverse | CAAAGGACCAAAGACCTCCA | INTEGRATED DNA TECHNOLOGIES |
| Mouse *Shmt2*_Forward | ATGCAGACCAGCTGACCAC | INTEGRATED DNA TECHNOLOGIES |
| Mouse *Shmt2*_Reverse | AGTGGCTAGTCCTCCTGTGC | INTEGRATED DNA TECHNOLOGIES |
| Mouse *Shmt1*_Forward | GCTGCGACAACATCTTCTCAT | INTEGRATED DNA TECHNOLOGIES |
| Mouse *Shmt1*_Reverse | CCTTTTCACAAAATCCACGC | INTEGRATED DNA TECHNOLOGIES |
| Mouse *Cth*_Forward | CAGAGGCATGGGTCATGATT | INTEGRATED DNA TECHNOLOGIES |
| Mouse *Cth*_Reverse | TGCTAAGGCCTTCCTCAAAA | INTEGRATED DNA TECHNOLOGIES |
| Mouse *Bax*_Forward | GATCAGCTCGGGCACTTTAG | INTEGRATED DNA TECHNOLOGIES |
| Mouse *Bax*_Reverse | TTGCTGATGGCAACTTCAAC | INTEGRATED DNA TECHNOLOGIES |
| Mouse *Dr5*_Forward | GGTCCTCTTGATGGGCTCTC | INTEGRATED DNA TECHNOLOGIES |
| Mouse *Dr5*_Reverse | GTTGCTGCTTGCTGTGCTAC | INTEGRATED DNA TECHNOLOGIES |
| Mouse *Bid*_Forward | GTGTAGCTCCAAGCACTGCC | INTEGRATED DNA TECHNOLOGIES |
| Mouse *Bid*_Reverse | GCAAACCTTTGCCTTAGCC | INTEGRATED DNA TECHNOLOGIES |
| Mouse *Fas*_Forward | CCTCAGCTTTAAACTCTCGGA | INTEGRATED DNA TECHNOLOGIES |
| Mouse *Fas*_Reverse | CAGACATGCTGTGGATCTGG | INTEGRATED DNA TECHNOLOGIES |
| Mouse *Puma*_Forward | TGTCGATGCTGCTCTTCTTG | INTEGRATED DNA TECHNOLOGIES |
| Mouse *Puma*_Reverse | GTGTGGAGGAGGAGGAGTGG | INTEGRATED DNA TECHNOLOGIES |
| Mouse *Ly6g*_Forward | TTGCAAAGTCCTGTGTGCTC | INTEGRATED DNA TECHNOLOGIES |
| Mouse *Ly6g*_Reverse | AGGGGCAGGTAGTTGTGTTG | INTEGRATED DNA TECHNOLOGIES |
| Mouse *Ccl2*_Forward | ATGAGATCAGAACCTACAACT | INTEGRATED DNA TECHNOLOGIES |
| Mouse *Ccl2*_Reverse | TCCTACAGAAGTGCTTGAG | INTEGRATED DNA TECHNOLOGIES |
| Mouse *Ccl19*_Forward | CTTCAGCCT GCTGGTTCTCT | INTEGRATED DNA TECHNOLOGIES |
| Mouse *Ccl19*_Reverse | GGAAGGCTTTCACGATGTTC | INTEGRATED DNA TECHNOLOGIES |
| Mouse *Il23a*_Forward | GCTCCCCTTTGAAGATGTCA | INTEGRATED DNA TECHNOLOGIES |
| Mouse *Il23a*_Reverse | GACCCACAAGGACTCAAGGA | INTEGRATED DNA TECHNOLOGIES |
| Mouse *Slc3a2*_Forward | TGCTCAGGCTGACATTGTAGC | INTEGRATED DNA TECHNOLOGIES |
| Mouse *Slc3a2*_Reverse | TCAGCCAAGTACAAGGGTGC | INTEGRATED DNA TECHNOLOGIES |
| Mouse *Sars*_Forward | AGTTGTCTGCCCGAAATCTG | INTEGRATED DNA TECHNOLOGIES |
| Mouse *Sars*_Reverse | ATTCGAGAGACGCAGGAGAA | INTEGRATED DNA TECHNOLOGIES |
| Mouse *Cd44*_Forward | CAGAGGCGACTAGATCCCTC | INTEGRATED DNA TECHNOLOGIES |
| Mouse *Cd44*_Reverse | GAGTCACAGTGCGGGAACTC | INTEGRATED DNA TECHNOLOGIES |
| Mouse *Gpc3*_Forward | CCCTGAATCTCGGAATTGAA | INTEGRATED DNA TECHNOLOGIES |
| Mouse *Gpc3*_Reverse | AGTCCCTGGCAGTAAGAGCA | INTEGRATED DNA TECHNOLOGIES |
| Mouse *Epcam*_Forward | CTGGCGTCTAAATGCTTGGC | INTEGRATED DNA TECHNOLOGIES |
| Mouse *Epcam*_Reverse | TCGTACAGCCCATCGTTGTTC | INTEGRATED DNA TECHNOLOGIES |
| Mouse *Timp1*_Forward | GCAACTCGGACCTGGTCATAA | INTEGRATED DNA TECHNOLOGIES |
| Mouse *Timp1*_Reverse | CGGCCCGTGATGAGAAACT | INTEGRATED DNA TECHNOLOGIES |
| Mouse *Col1a1*_Forward | GCTCCTCTTAGGGGCCACT | INTEGRATED DNA TECHNOLOGIES |
| Mouse *Col1a1*_Reverse | CCACGTCTCACCATTGGGG | INTEGRATED DNA TECHNOLOGIES |
| Mouse *Acta2*_Forward | GTCCCAGACATCAGGGAGTAA | INTEGRATED DNA TECHNOLOGIES |
| Mouse *Acta2*_Reverse | TCGGATACTTCAGCGTCAGGA | INTEGRATED DNA TECHNOLOGIES |
| Mouse *Il1b*_Forward | GGTCAAAGGTTTGGAAGCAG | INTEGRATED DNA TECHNOLOGIES |
| Mouse *Il1b*_Reverse | TGTGAAATGCCACCTTTTGA | INTEGRATED DNA TECHNOLOGIES |
| Mouse *Ccnd1*_Forward | TTGTGCATCTACACTGACAACTC | INTEGRATED DNA TECHNOLOGIES |
| Mouse *Ccnd1*_Reverse | AGGGTGGGTTGGAAATGAACT | INTEGRATED DNA TECHNOLOGIES |
| Mouse *Il6*_Forward | CGCTATGAAGTTCCTCTCTGC | INTEGRATED DNA TECHNOLOGIES |
| Mouse *Il6*_Reverse | TCTGCAAGTGCATCATCGTT | INTEGRATED DNA TECHNOLOGIES |
| Mouse *Tgfb*_Forward | GGAGAGCCCTGGATACCAAC | INTEGRATED DNA TECHNOLOGIES |
| Mouse *Tgfb*_Reverse | AAGTTGGCATGGTAGCCCTT | INTEGRATED DNA TECHNOLOGIES |
| Mouse *Dlk1*_Forward | GGC CAT CGT CTT TCT CAA CA | INTEGRATED DNA TECHNOLOGIES |
| Mouse *Dlk1*_Reverse | CTC CTC ATC ACC AGC CTC CT | INTEGRATED DNA TECHNOLOGIES |
| Mouse *Ctgf*_Forward | TGACCTGGAGGAAAACATTAAGA | INTEGRATED DNA TECHNOLOGIES |
| Mouse *Ctgf*_Reverse | AGCCCTGTATGTCTTCACACTG | INTEGRATED DNA TECHNOLOGIES |
| Mouse *Col4a1*_Forward | CTGGCACAAAAGGGACGAG | INTEGRATED DNA TECHNOLOGIES |
| Mouse *Col4a1*_Reverse | ACGTGGCCGAGAATTTCACC | INTEGRATED DNA TECHNOLOGIES |

- 1. **Biological samples**

| **Description** | **Source** | **Identifier** |
| --- | --- | --- |
|  |  |  |

- 1. **Deposited data**

| **Name of repository** | **Identifier** | **Link** |
| --- | --- | --- |
| Gene Expression Omnibus | GSE191115 | https://www.ncbi.nlm.nih.gov/geo/query/acc.cgi?acc=GSE19115 |

- 1. **Software**

| **Software name** | **Manufacturer** | **Version** |
| --- | --- | --- |
| ImageJ | Open Source/National  Institutes of Health | 1.52 |
| GraphPad Prism 7 | GraphPad Software, Inc. | 7.0a |
| Adobe Illustrator CS6 | Adobe | 16.0.0 |

- 1. **Other (e.g. drugs, proteins, vectors etc.)**

| Collagen I, Rat Tail | Corning | Cat# 354236 |
| --- | --- | --- |
| Liberase-TM | Sigma-Aldrich | Cat# 5401127001 |
| Complete^™^, Mini Protease Inhibitor Cocktail | Roche | Cat#11836153001 |
| Phosphatase Inhibitor Cocktail 2 | Sigma-Aldrich | Cat#P5726 |
| Clarity Western ECL Substrate | Biorad | Cat#[1705061](https://www.bio-rad.com/en-us/sku/1705061-clarity-western-ecl-substrate-500-ml) |
| Protein Assay Dye reagent concentrate | Biorad | Cat#5000006 |
| Pluronic F68 | Thermo Fisher Scientific | Cat# 24040032 |
| Polybrene | Santa Cruz | Cat# sc-134220 |
| Lipofectamine 3000 Transfection Reagent | Thermo Fisher Scientific | Cat# L3000015 |
| polyethylenimine (PEI) | Millipore Sigma | Cat# 764965 |
| 100-kDa filters | Millipore Sigma | Cat# ufc810024 |
| Diethylnitrosamine (DEN) | Millipore Sigma | Cat# N0258 |
| Tunicamycin (TM) | Millipore Sigma | Cat# T7765 |
| Nuclear-fast red | Aladdin | Cat# N276754 |
| Dihydroethidium (DHE) | Apexbio | Cat# C3807 |
| potassium ferrocyanide | Aladdin | Cat# P112421-100g |
| RSL3 | MedChemExpress | Cat# HY-100218A |
| Deferoxamine mesylate | MedChemExpress | Cat# HY-B0988 |
| 2-Mercaptoethanol | Aladdin | Cat# M301574 |
| Trypan blue dye (0.4%) | Solarbio | Cat# C0040 |
| Z-VAD-FMK | Selleckchem | Cat# S7023 |
| MG132 | Apexbio | Cat# A2585 |
| Palmitic acid (PA) | Millipore Sigma | Cat# P5585 |
| necrostatin-1 (NEC-1) | Selleckchem | Cat# S8037 |
| ferrostatin-1 (FER-1) | Selleckchem | Cat# S7243 |
| 10x RBC Lysis Buffer | eBioscience | Cat# 00-4300-54 |
| Critical commercial assays |  |  |
| Triglyceride colorimetric assay kit | Cayman Chemical | Cat#10010303 |
| Cholesterol fluorometric assay kit | Cayman Chemical | Cat#10007640 |
| Cholesterol/ Cholesteryl Ester Assay Kit | Abcam | Cat# ab65359 |
| RNeasy Plus Mini Kit | Qiagen | Cat#74134 |
| SuperScript™ VILO™ cDNA Synthesis Kit | Invitrogen | Cat#11754050 |
| SsoAdvanced^TM^ Universal SYBR Green Supermix | Biorad | Cat#1725275 |
| Cell Counting Kit-8 (CCK-8) | Glpbio | Cat # GK10001 |
| Infinity ALT (GPT) reagent | Thermo Fisher Scientific | Cat# TR71121 |
| In Situ Cell Death Detection Kit, TMR red (TUNEL assay) | Roche | Cat# 12156792910 |
| NE-PER™ Nuclear and Cytoplasmic Extraction Reagents | Thermo Fisher Scientific | Cat# 78833 |
| DAB Peroxidase (HRP) Substrate Kit (with Nickel),  3,3’-diaminobenzidine | Vector Laboratories | Cat# SK-4100 |
| ImmPRESS™ Excel Amplified HRP Polymer Staining Kit (Anti-Rabbit IgG) | Vector Laboratories | Cat# MP-7601 |
| ImmPRESS™ HRP Anti-Rabbit IgG (Peroxidase) Polymer Detection Kit | Vector Laboratories | Cat# MP-7401 |
| RNAscope 2.5 HD Assay kit- BROWN | ACDbio/Biotechne | Cat# 405101 |
| GSH/GSSG-Glo™ Assays | Promega | Cat# V6611 |
|  |  |  |

- 1. **Please provide the details of the corresponding methods author for the manuscript:**

| Michael Karin,  Laboratory of Gene Regulation and Signal Transduction, Departments of Pharmacology and Pathology, University of California San Diego, 9500 Gilman Drive, La Jolla, San Diego, CA 92093, USA;  Tel.: +1 858 534 1361; fax: +1 858 534 0848; email: [karinoffice@ucsd.edu](mailto:karinoffice@ucsd.edu);  Feng He,  Academy of Integrative Medicine, Shanghai University of Traditional Chinese Medicine, 1200 Cailun Road, Shanghai 201203, China.  Tel.: +86 21 52322501, email: fhe@shutcm.edu.cn; |
| --- |

**2.0 Please confirm for randomised controlled trials all versions of the clinical protocol are included in the submission. These will be published online as supplementary information.**

| **NA** |
| --- |
