# Supplementary material for: ATF4 suppresses hepatocarcinogenesis by inducing SLC7A11 (xCT) to block stress-related ferroptosis
Source: J Hepatol. Author manuscript; Available in PMC 2024 Aug 19. (PMC11332364; doi:10.1016/j.jhep.2023.03.016)
Supplement: Data 1 [file NIHMS2013638-supplement-Data_1.pdf]

ATF4 suppresses hepatocarcinogenesis by inducing SLC7A11 (xCT) to block stress-related ferroptosis

Feng He, Peng Zhang, Junlai Liu, Ruolei Wang, Randal J Kaufman, Benjamin C Yaden, Michael Karin

Table of contents

Supplementary Methods..2

Supplementary Figures………..7

Supplementary Tables…..21

Supplementary References...26

Supplementary Methods

In vivo treatments

2 mo male *Atf4^{F/F}*, *Atf4^{Δhep}*, *MUP-uPA/Atf4^{F/F}*, and *MUP-uPA/Atf4^{Δhep}* mice were injected with AAV8-mCherry or AAV8-xCT (8×10^{10} genome copies/mice) via the tail vein. *MUP-uPA/Atf4^{F/F}* and *MUP-uPA/Atf4^{Δhep}* mice were fed with HFD from 3 mo, until 10 mo when sacrificed for the tumor analysis. LFD-fed AAV8-injected *MUP-uPA/Atf4^{Δhep}* mice were sacrificed at 3.5 mo to analyze liver injury. AAV8-injected *Atf4^{F/F}* and *Atf4^{Δhep}* mice were used for the HFD-fed DEN-induced HCC model described above until sacrificed at 10 mo for sample collection. For acute DEN-induced liver injury, 2-3 mo male *Atf4^{F/F}* and *Atf4^{Δhep}* mice were *i.p.* injected with 100 mg/kg DEN. After 24 and 48 hrs, livers and serum were collected. For FER-1 administered *MUP-uPA/Atf4^{Δhep}* mice, 6-wo mice were *i.p.* injected with vehicle control DMSO or 1 mg/kg FER-1 every other day for 2 weeks before livers and serum were analyzed. Only male mice were used and the number of mice per experiment and their age are indicated in the figure legends.

Sample collection and Histology

Mouse liver was removed, weighed, and photographed before separation into individual lobes. Large lobes were fixed in 10% formalin for 24-48 hrs to prepare paraffin blocks or embedded in Tissue-Tek OCT compound (Sakura Finetek) for frozen block preparation. Frozen tissue sections were stained with Oil Red O (ORO) to detect lipids or dihydroethidium (DHE) for ROS. Formalin-fixed paraffin-embedded (FFPE) tissues were used for hematoxylin and eosin (H&E), Sirius Red staining, and IHC staining. Positively stained areas were quantified in 3-6 random fields (× 100, × 200, or × 400) on each slide using Image J software. The remaining lobes were dissected and stored at −80° C until analyzed.

5 µm thick sections were stained with H&E and processed for IHC as described before [1]. Briefly, deparaffinized and dehydrated sections were blocked with 5% goat serum for 1 hr at room temperature, primary antibodies for 1 hr at room temperature, followed by incubation with biotinylated secondary antibodies (1:200) for 30 min and streptavidin-HRP (1:500) for 30 min. Bound peroxidase was visualized by 1-10 min of incubation in a 3, 3’-diaminobenzidine (DAB)

solution (Vector Laboratories, SK-4100). Sections were washed, counterstained with hematoxylin, dehydrated, and mounted. Antibodies are listed in Supplementary table S1.
Serum ALT levels were measured using Infinity ALT (GPT) reagent (Thermo scientific TR71121) according to the supplied protocol. Liver triglycerides (TG), serum TG, and serum cholesterol were measured with a Triglyceride Colorimetric Assay Kit (Cayman Chemical 10010303) and a Cholesterol Fluorometric Assay Kit (Cayman Chemical #10007640), respectively, according to the manufacturer's protocol. Liver cholesterol was measured with a Cholesterol/Cholesteryl Ester Assay Kit (Abcam ab65359) according to the manufacturer's protocol. Liver and hepatocytes GSH and GSSG measurements were performed using GSH/GSSG-Glo™ Assays (Promega V6611) according to the manufacturer's protocol. TUNEL staining was performed using an in-situ cell death detection kit (Roche 12156792910). Images were captured on an upright light/fluorescent microscope (Zeiss) equipped with an AxioCam camera.

Prussian Blue staining of ferric iron

4 µm thick FFPE sections were deparaffinized, hydrated, and stained for 30 min at room temperature in the fresh 5% potassium ferrocyanide (Aladdin P112421) and 5% hydrochloric acid, before being counted-stained with Nuclear-fast red for 5 min (Aladdin N276754) [2].

In Situ Hybridization (ISH)

For ATF4 ISH, ATF4 probes and a detection kit from RNAscope Probe-Mm-*Atf4* (ACD Bio 405101) were used and mouse liver sections were stained according to the manufacturer's protocol RNAscope® 2.5 HD Assay- BROWN.

Cell Culture and In Vitro Treatments

Primary hepatocytes were isolated from age and sex-matched mice by portal vein liberase perfusion (Roche 05401127001) as described [1]. Hepatocytes were purified by low speed (50 g) differential centrifugation and repeated washes (3x) with Phosphate Buffered Saline (PBS). Cells were cultured in DMEM medium (Gibco 11995-065), supplemented with 10% FBS, penicillin (100 mg/ml), and streptomycin (100 mg/ml) in type-1 collagen (Rat tail, Corning 354236) coated plates.

Infection with adenovirus was performed 6 hrs after seeding on the collagen-coated plates at a moi of 10. After 24 hrs infection, cells were further treated as indicated before being collected for protein and RNA analysis.

For isolation of tumor cells from mice, tumors were dissected from livers and cut into pieces of about 1 mm^3, followed by digestion with liberase and removal of red blood cells (RBC) with 1XRBC lysis buffer (eBioscience 00-4300-54) before centrifugation and culturing.

All cells were incubated at 37 °C in a humidified chamber with 5% CO_2.

Cell Counting Kit-8 (CCK-8) cell viability assay

Primary hepatocytes seeded in 96-well plates overnight were treated with 300 μM palmitic acid (PA, Millipore Sigma P5585), 15 ug/ml tunicamycin (TM, Millipore Sigma T7765), 10 mM diethylnitrosamine (DEN, Millipore Sigma N0258), 5 uM RSL3 (MedChemExpress HY-100218A), or MG132 (Apexbio A2585) for the indicated time periods. For the treatment with inhibitors, 50 μM apoptosis inhibitor Z-VAD-FMK (Selleckchem S7023), 10 μM necroptosis inhibitor necrostatin-1 (NEC-1, Selleckchem S8037), 50 μM iron chelator deferoxamine (DFO), 100 μM β-mercaptoethanol (β-Me), or 10 μM ferroptosis inhibitor FER-1 (Selleckchem S7243) were added with cell death inducers at the same time. At the indicated time points, 10 μl CCK-8 (GLPBIO GK10001) was added to each well to measure viability according to the manufacturer's protocol. After 1-2 hrs, cell viability was determined by measuring optical density (OD) at 450 nm. In some experiments, trypan blue dye (Solarbio C0040) exclusion counting was performed. Cell viability under basal conditions is the viability of vehicle control.

Protein extraction and immunoblots

Livers were homogenized in a Dounce homogenizer (Thomas Scientific, NJ) with 30 strokes in RIPA buffer containing protease and phosphatase inhibitor cocktails. Primary hepatocytes were

lysed directly in RIPA buffer containing protease and phosphatase inhibitor cocktails. After centrifugation, supernatants were separated by SDS-PAGE. Nuclear extraction was performed by using NE-PER™ Nuclear and Cytoplasmic Extraction kit (Thermo Fisher Scientific, 78833) and cytosolic and nuclear factions were separated according to the manufacturer's protocol. Protein concentrations were quantified by Bradford assay (Biorad 5000006) and analyzed by SDS-PAGE and IB. Details of primary and secondary antibodies are provided in Supplementary Table S1.

RNA isolation and Quantitative real-time PCR (Q-RT-PCR)

Total RNA from liver tissue or primary hepatocytes was extracted using RNeasy Plus Mini kit (Qiagen Cat 74134) and cDNA was synthesized using SuperScript™ VILO™ cDNA Synthesis Kit (Thermo Fisher Scientific, 11754050). Q-RT-PCR was performed using SYBR green (Biorad 1725275) based QPCR on a Biorad CFX96 machine. Relative mRNA expression was calculated from the comparative cycle threshold (CT) values relative to Hypoxanthine Phosphoribosyltransferase 1 (HPRT1) mRNA. Data are presented as arbitrary units and were calculated by the 2ˆ(–delta CT) method. Primer sequences were provided by Integrated DNA technologies and are listed in Supplementary Table S2.

Transmission Electron Microscopy (TEM)

Primary hepatocytes were seeded at a density of $4X10^6$ cells/dish in 10-cm tissue culture dishes pre-coated with type-1 collagen (Rat tail, Corning 354236). Overnight cultured cells were treated with vehicle (DMSO) or RSL3 (2 μM) for 12 hrs. Cells were fixed with 2.5% glutaraldehyde in 0.1 M Phosphate buffer (PB, 0.1 M H_2PO_4, 0.1 M HPO_4 [pH 7.2]) for at least 2 hrs, and then treated with 1% OsO_4 in 0.1 M PB at 4 °C for 1 hr. Enblock staining used 2% aqueous uranyl acetate for 2 hrs at 4°C in dark. After dehydration through ethanol and acetone series, cells were embedded in pure epoxy resin. 70 nm of sections were cut on a Leica ultramicrotome (Leica 705902), stained with 0.4% lead citrate, and examined under a FEI Tecnai G2 Spirit BioTWIN

electron microscope (FEI, Hillsboro, OR, USA). Pictures were taken on a Gatan Digital image capturing system digital camera at 8,200–11,500-fold magnification at the Electron Microscopy Core Facility, Shanghai University of Traditional Chinese Medicine.

Adenovirus preparation

Adenoviruses expressing GFP, ATF4, or xCT were generated using pAdTrack-CMV and the AdEasy system as described [1]. Adenoviruses were harvested and purified by CsCl ultracentrifugation and their titter was determined as described [1].

Adeno-associated virus type 8 (AAV8) preparation

PCR-amplified mouse xCT sequences were cloned into a pAAV-mCherry vector, a generous gift from Kang Zhang (University of California, San Diego). AAV8 was prepared as described [3]. Briefly, each virus was produced in 5 × 15 cm plates, where each plate was transfected with 7.5 μg of pXR-capsid (pXR-8), 7.5 of μg recombinant transfer vector, and 22.5 μg of pHelper vector using polyethylenimine (PEI 1 μg/μL, Millipore Sigma, 764965) at a PEI: DNA mass ratio of 4:1. The mixture was incubated for 10 min at room temperature and then applied dropwise onto the media. The virus was harvested after 72 hrs and purified using HiTrap SP HP (GE Healthcare Life Sciences, 17-1151-01) column method. The virus was then dialyzed with 1× PBS (pH 7.2) supplemented with 50 mM NaCl and 0.0001% of Pluronic F68 (Thermo Fisher Scientific, 24040032) using 100-kDa filters (Millipore Sigma, UFC810024) to a final volume of ~1 mL and quantified by QPCR using primers specific to the ITR region, against a standard (ATCC VR-1616): AAV8-ITR-F: 5′-CGGCCTCAGTGAGCGA-3′ and AAV8-ITR-R: 5′-GGAACCCCTAGTGATGGAGTT-3′.

Supplementary Figures

Figure. S1

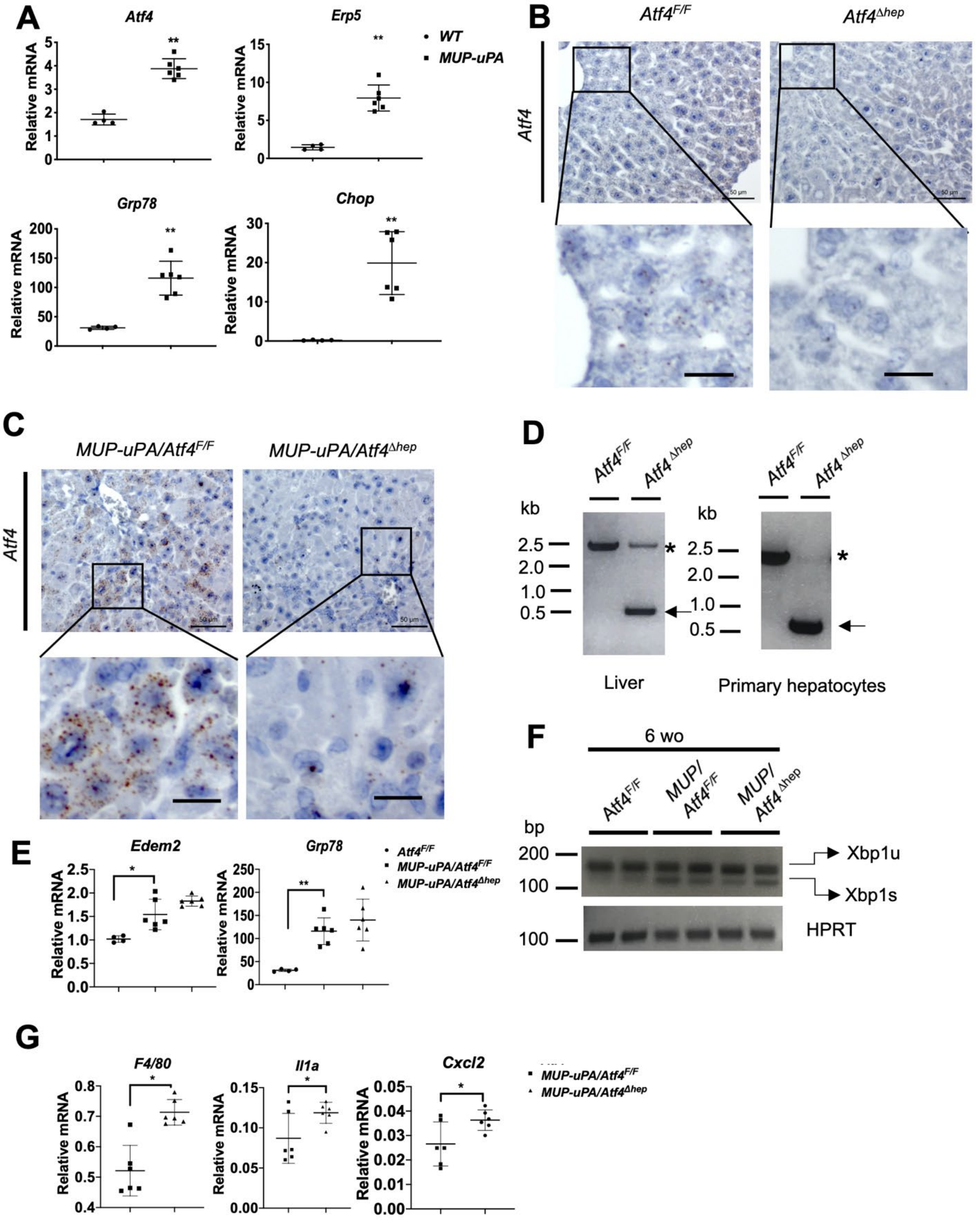

A
Atf4
Erp5
Grp78
Chop
Relative mRNA
WT
MUP-uPA
B
Atf4F/F
Atf4Δhep
Atf4
C
MUP-uPA/Atf4F/F
MUP-uPA/Atf4Δhep
Atf4
D
Atf4F/F
Atf4Δhep
kb
Liver
Primary hepatocytes
E
Edem2
Grp78
Atf4F/F
MUP-uPA/Atf4F/F
MUP-uPA/Atf4Δhep
F
6 wo
Atf4F/F
MUP/ Atf4F/F
MUP/ Atf4Δhep
bp
Xbp1u
Xbp1s
HPRT
G
F4/80
Il1a
Cxcl2
MUP-uPA/Atf4F/F
MUP-uPA/Atf4Δhep

Fig. S1. Hepatocyte-specific ATF4 ablation does not affect XBP1 and ATF6 UPR pathways. (A) Q-RT-PCR analysis of liver mRNAs from 6-wo WT and *MUP-uPA* mice. Mean ± SD (n=4-6/group). $^{**}p < 0.01$ (Student's t-test). (B) and (C) In situ hybridization (RNAScope/ISH) analysis of *Atf4* mRNA in 6-wo $Atf4^{F/F}$, $Atf4^{\Delta hep}$ (B), $MUP\text{-}uPA/Atf4^{F/F}$, and $MUP\text{-}uPA/Atf4^{\Delta hep}$ (C) mice. Bottom panels: enlarged images of ROI. Scale bars, top 50 μm; bottom, 20 μm. (D) ATF4 deletion efficiency in livers and primary hepatocytes of 6-wo $Atf4^{F/F}$ *and* $Atf4^{\Delta hep}$ mice. Asterix marks the floxed allele and the arrow indicates the deleted allele. (E) Q-RT-PCR analysis of liver mRNAs from 6-wo LFD-fed $Atf4^{F/F}$, $MUP\text{-}uPA/Atf4^{F/F}$, and $MUP\text{-}uPA/Atf4^{\Delta hep}$ mice. Mean ± SD (n=4-6/group). $^{*}p < 0.05$; $^{**}p < 0.01$ (Student's t-test). (F) RT-PCR analysis of liver XBP1 splicing in 6-wo $Atf4^{F/F}$, $MUP\text{-}uPA/Atf4^{F/F}$, and $MUP\text{-}uPA/Atf4^{\Delta hep}$ mice. G, Q-RT-PCR analysis of macrophage marker and inflammatory cytokines in livers of 6-wo $MUP\text{-}uPA/Atf4^{F/F}$ and $MUP\text{-}uPA/Atf4^{\Delta hep}$ mice. Mean ± SD (n=4-6/group). $^{*}p < 0.05$ (Student's t-test). ROI, region of interest. ISH, in situ hybridization.

Figure. S2

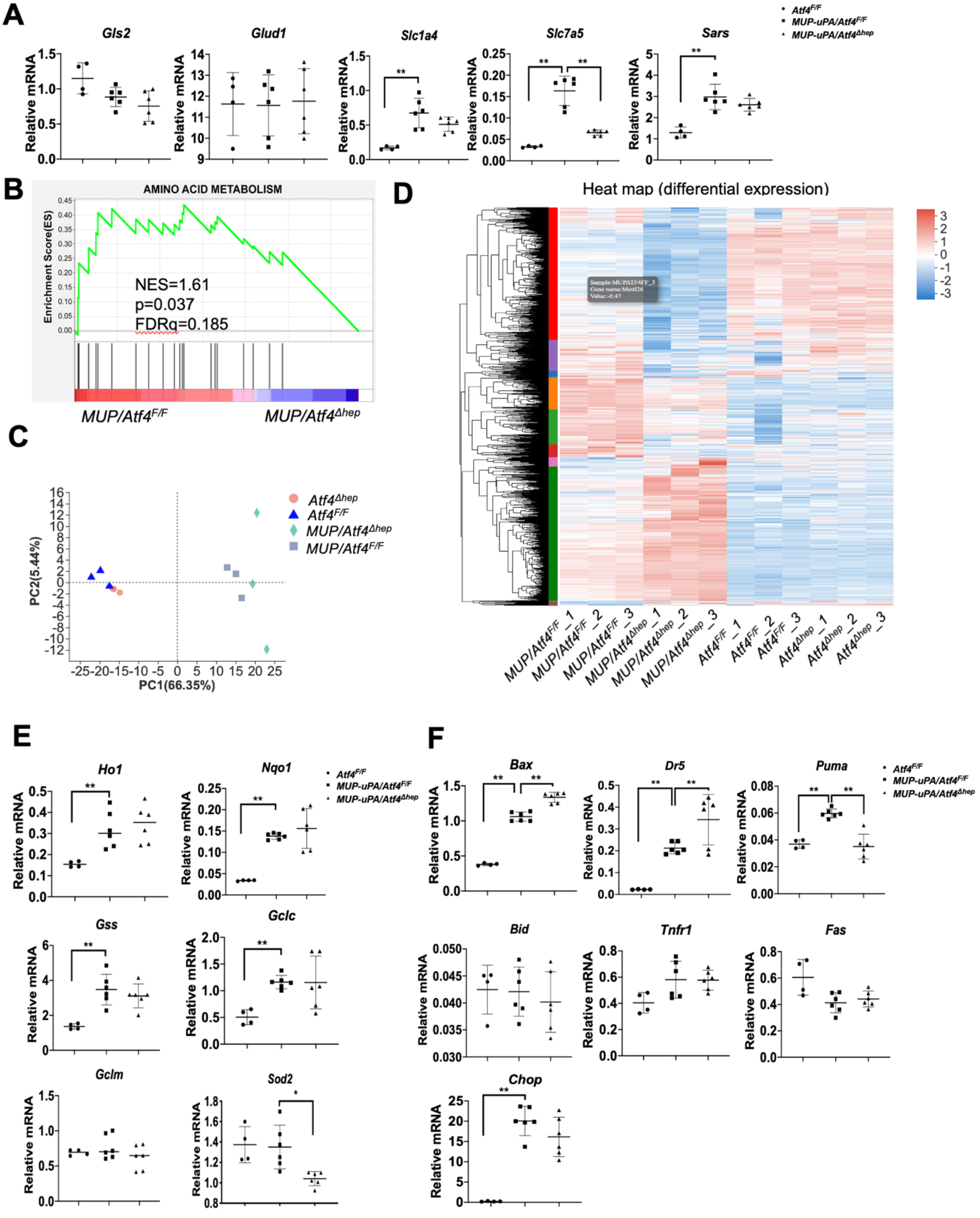
A
Gls2
Glud1
Slc1a4
Slc7a5
Sars
Relative mRNA
Atf4F/F
MUP-uPA/Atf4F/F
MUP-uPA/Atf4Δhep
B
AMINO ACID METABOLISM
Enrichment Score(ES)
NES=1.61
p=0.037
FDRq=0.185
MUP/Atf4F/F
MUP/Atf4Δhep
C
PC2(5.44%)
PC1(66.35%)
Atf4Δhep
Atf4F/F
MUP/Atf4Δhep
MUP/Atf4F/F
D
Heat map (differential expression)
MUP/Atf4F/F _1
MUP/Atf4F/F _2
MUP/Atf4F/F _3
MUP/Atf4Δhep _1
MUP/Atf4Δhep _2
MUP/Atf4Δhep _3
Atf4F/F _1
Atf4F/F _2
Atf4F/F _3
Atf4Δhep _1
Atf4Δhep _2
Atf4Δhep _3
E
Ho1
Nqo1
Gss
Gclc
Gclm
Sod2
F
Bax
Dr5
Puma
Bid
Tnfr1
Fas
Chop

Fig. S2. ATF4 ablation differentially affects expression of genes involved in glutaminolysis, antioxidant response, amino acid metabolism, and cell death. (A) Q-RT-PCR analysis of liver mRNAs involved in amino acid metabolism. Mean ± SD (n=4-6/group). ***$p < 0.01$ (Student's t-test). (B) GSEA of RNA-Seq data from livers of overnight fasted 6-wo *MUP-uPA/Atf4*$^{\Delta hep}$ and *MUP-uPA/Atf4*$^{F/F}$ mice (n=3) showing enrichment of amino acid metabolism in the *MUP-uPA/Atf4*$^{F/F}$ mice. (C) PCA of normalized RNA-Seq data TPM of livers from overnight fasted 6-wo *MUP-uPA/Atf4*$^{F/F}$, *MUP-uPA/Atf4*$^{\Delta hep}$, *Atf4*$^{F/F}$, and *Atf4*$^{\Delta hep}$ mice (n=3). (D) Heatmap representation of above RNA-Seq data. (E-F) Q-RT-PCR analysis of liver mRNAs involved in the NRF2-stimulated antioxidant response (E) and cell death (F) in 6-wo *Atf4*$^{F/F}$, *MUP-uPA/Atf4*$^{F/F}$, and *MUP-uPA/Atf4*$^{\Delta hep}$ mice. Mean ± SD (n=4-6/group). *$p < 0.05$; **$p < 0.01$ (Student's t-test). GESA, Gene set enrichment analysis; PCA, Principal component analysis; TPM, transcripts per million.

Figure. S3

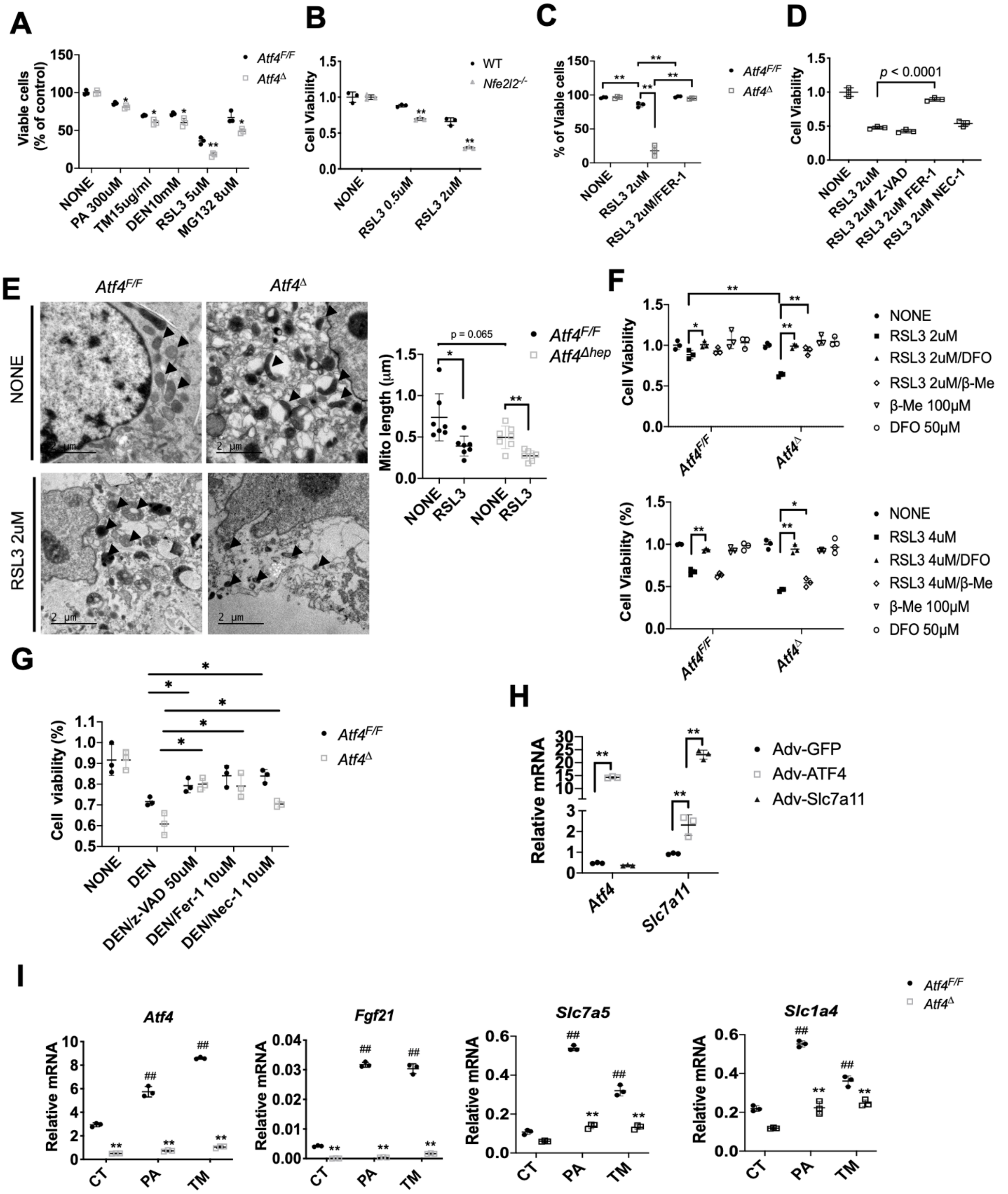
A
Viable cells (% of control)
Atf4F/F
Atf4Δ
NONE
PA 300uM
TM15ug/ml
DEN10mM
RSL3 5uM
MG132 8uM
B
Cell Viability
WT
Nfe2l2-/-
NONE
RSL3 0.5uM
RSL3 2uM
C
% of Viable cells
Atf4F/F
Atf4Δ
NONE
RSL3 2uM
RSL3 2uM/FER-1
D
Cell Viability
p < 0.0001
NONE
RSL3 2uM
RSL3 2uM Z-VAD
RSL3 2uM FER-1
RSL3 2uM NEC-1
E
Atf4F/F
Atf4Δ
NONE
RSL3 2uM
2 μm
p = 0.065
Mito length (μm)
Atf4F/F
Atf4Δhep
NONE
RSL3
F
Cell Viability
NONE
RSL3 2uM
RSL3 2uM/DFO
RSL3 2uM/β-Me
β-Me 100μM
DFO 50μM
Atf4F/F
Atf4Δ
Cell Viability (%)
NONE
RSL3 4uM
RSL3 4uM/DFO
RSL3 4uM/β-Me
β-Me 100μM
DFO 50μM
G
Cell viability (%)
Atf4F/F
Atf4Δ
NONE
DEN
DEN/z-VAD 50uM
DEN/Fer-1 10uM
DEN/Nec-1 10uM
H
Relative mRNA
Adv-GFP
Adv-ATF4
Adv-Slc7a11
Atf4
Slc7a11
I
Atf4F/F
Atf4Δ
Atf4
Fgf21
Slc7a5
Slc1a4
Relative mRNA
CT
PA
TM

Fig. S3. ATF4 ablation increases hepatocyte susceptibility to cell death inducers. (A) Viability of *Atf4*$^{F/F}$ and *Atf4*$^{\Delta}$ hepatocytes treated with different cell death inducers. Cell viability was assessed by Trypan Blue exclusion in triplicate and the effects of each treatment was expressed as a percentage of the vehicle control (NONE). *$p < 0.05$; **$p < 0.01$ (*vs Atf4*$^{F/F}$, Student's t-test). (B) Viability of WT and *Nfe2l2*$^{-/-}$ hepatocytes treated without or with 0.5 μM or 2 μM RSL3 for 10 hrs. **$p < 0.01$ (*vs* WT, Student's t-test). (C) Percentage (%) of viable cells in Figure 3C. **$p < 0.01$ (Student's t-test). (D) Viability of *Atf4*$^{\Delta}$ hepatocytes treated without or with 2 μM RSL3 in the absence or presence of 50 μM Z-VAD, 10 μM FER-1, or 10 μM NEC-1, respectively, for 10 hrs. *p* value indicates significance level, Student's t-test. (E) Left, transmission electron microscopy of WT and *Atf4*$^{\Delta}$ hepatocytes treated with DMSO or RSL3 (2 μM) for 12 hrs. Black arrowheads indicate mitochondria. Shrunken mitochondria were observed in ferroptotic hepatocytes. Scale bars, 2 μm. Right, mitochondrial length (along the long axis) was measured and summarized. (F) Viability of WT and *Atf4*$^{\Delta}$ hepatocytes treated without or with 2 μM (top) and 4 μM (bottom) RSL3 in the absence or presence of iron chelator deferoxamine (DFO, 50 μM) or β-mercaptoethanol (β-Me, 100 μM), respectively, for 12 hrs. *$p < 0.05$; **$p < 0.01$ (Student's t-test). (G) Viability of WT and *Atf4*$^{\Delta}$ hepatocytes treated without or with 10 mM DEN in the absence or presence of 50 μM Z-VAD, 10 μM FER-1, or 10 μM NEC-1, respectively, for 36 hrs. *$p < 0.05$; **$p < 0.01$ (Student's t-test). (H) Q-RT-PCR analysis of *Atf4* and *Slc7a11* mRNA expression in *Atf4*$^{\Delta}$ hepatocytes transduced with Adv-GFP, -ATF4, or -Slc7a11/xCT 24 hrs before culturing in the presence of 2 μM RSL3, as indicated. **$p < 0.01$ (Student's t-test). (I) Q-RT-PCR analysis of mRNA expression in *Atf4*$^{F/F}$ and *Atf4*$^{\Delta}$ hepatocytes cultured in NONE, 300 μM palmitic acid (PA) or 4 μg/ml tunicamycin (TM) for 10 hrs. Mean ± SD (n=3/group). **$p < 0.01$ (*vs Atf4*$^{F/F}$, Student's t-test). $^{\#\#}p < 0.01$ (*vs* CT of *Atf4*$^{F/F}$, Student's t-test). Q-RT-PCR, quantitative reverse-transcription

PCR; WT, wild-type; PA, palmitic acid; TM, tunicamycin; DEN, diethynitrosamine; RSL3, RAS-selective lethal 3; FER-1, ferrostatin-1; GSH, reduced glutathione; NEC-1, necrostatin-1; Adv, adenovirus; CT, Control; Mito, mitochondria.

Figure. S4

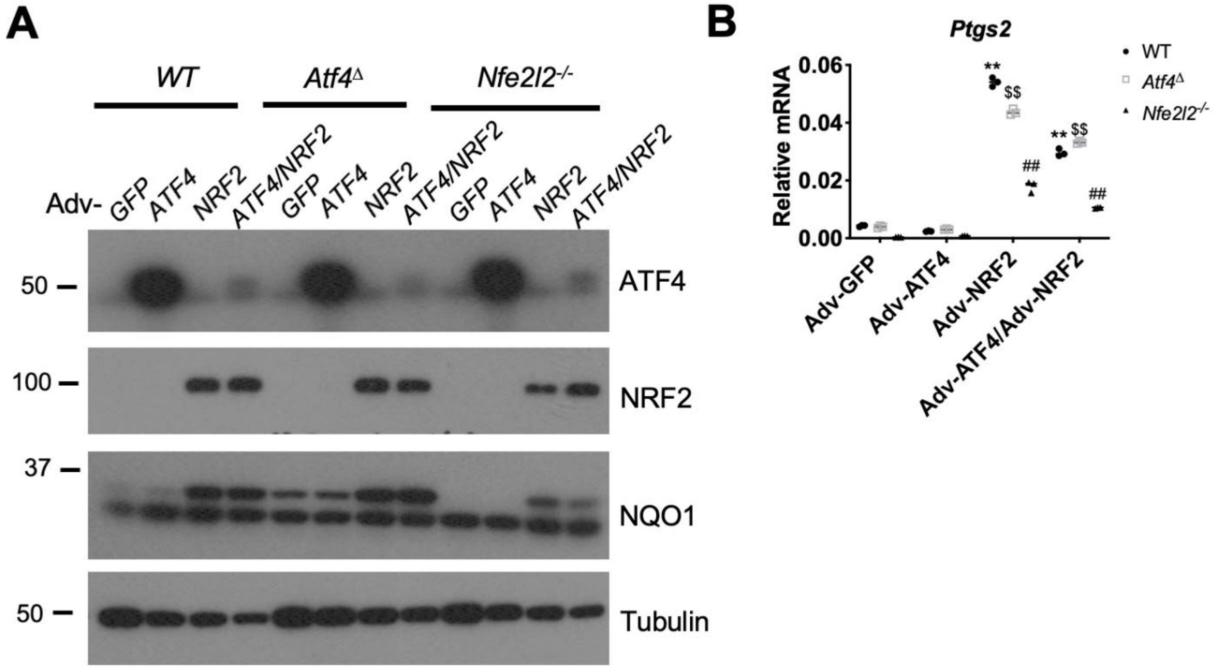

Fig. S4. NRF2 and ATF4 distinctly regulate genes involved in redox homeostasis. (A), Representative IB analysis of ATF4, NRF2, and NQO1 in WT, *Atf4*$^{\Delta}$, and *Nfe2l2*$^{-/-}$ hepatocytes transduced with Adv-GFP, -ATF4, -NRF2, or ATF4+NRF2. (B) Q-RT-PCR analysis of *Ptgs2* expression in above cells. Mean ± SD (n=3/group). $^{**}p < 0.01$ (*vs* Adv-GFP of WT hepatocytes, Student's t-test); $^{\$\$}p < 0.01$ (*vs* Adv-GFP of *Atf4*$^{\Delta}$ hepatocytes, Student's t-test); $^{\#\#}p < 0.01$ (*vs* Adv-GFP of *Nfe2l2*$^{-/-}$ hepatocytes, Student's t-test). Adv, adenovirus.

Figure. S5

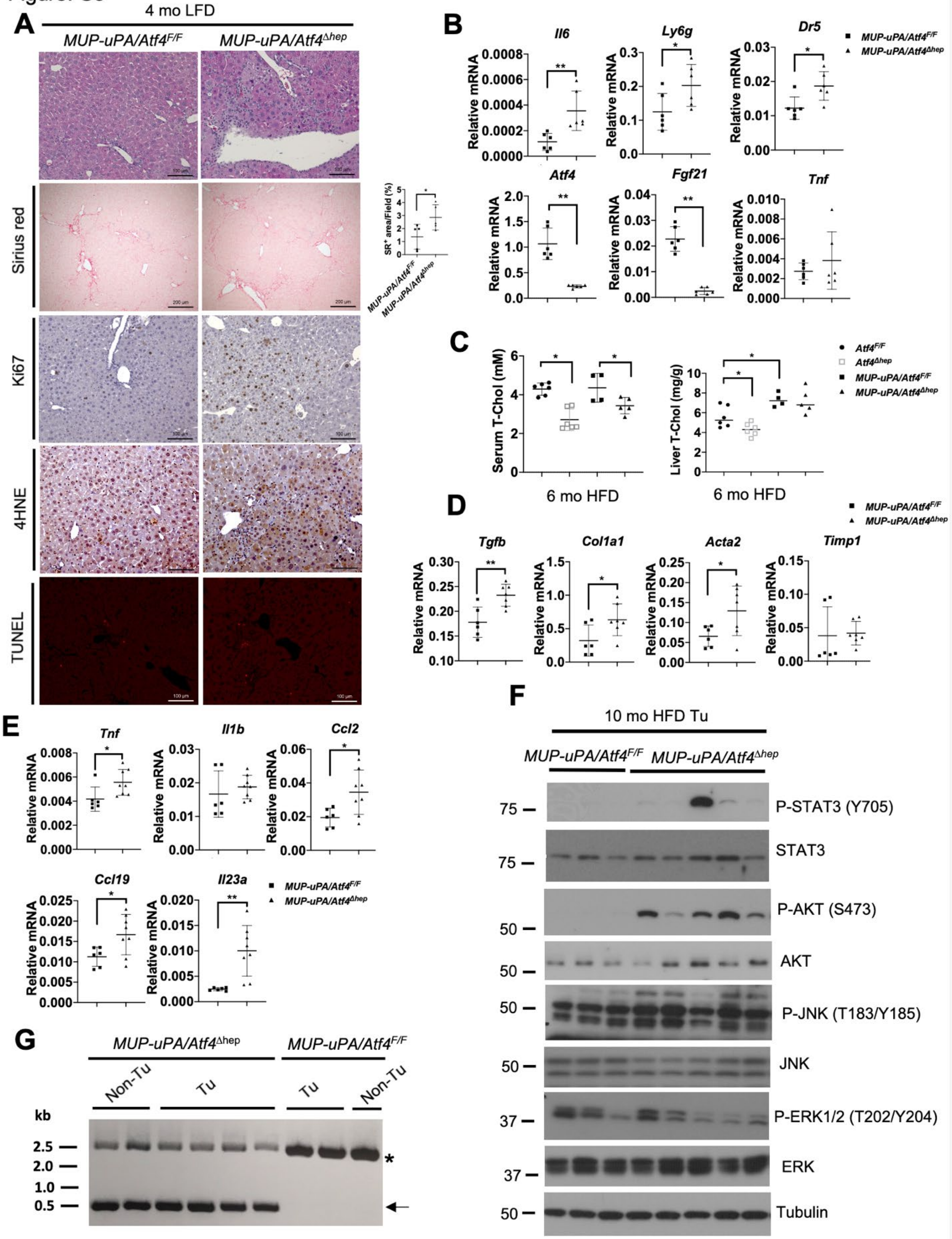
A
4 mo LFD
MUP-uPA/Atf4F/F
MUP-uPA/Atf4Δhep
Sirius red
Ki67
4HNE
TUNEL
SR+ area/Field (%)
B
Il6
Ly6g
Dr5
Atf4
Fgf21
Tnf
Relative mRNA
C
Atf4F/F
Atf4Δhep
Serum T-Chol (mM)
Liver T-Chol (mg/g)
6 mo HFD
D
Tgfb
Col1a1
Acta2
Timp1
E
Tnf
Il1b
Ccl2
Ccl19
Il23a
F
10 mo HFD Tu
P-STAT3 (Y705)
STAT3
P-AKT (S473)
AKT
P-JNK (T183/Y185)
JNK
P-ERK1/2 (T202/Y204)
ERK
Tubulin
G
Non-Tu
Tu
kb
2.5
2.0
1.0
0.5

Fig. S5. ATF4 ablation increases lipid peroxidation, liver damage, and HCC development in *MUP-uPA* mice. (A) H&E, Sirius red, Ki67, 4HNE, and TUNEL staining of 4-mo LFD-fed *MUP-uPA/Atf4*$^{F/F}$ and *MUP-uPA/Atf4*$^{\Delta hep}$ mice (n=3-5/group. Scale bars, 100 μm for H&E, IHC, and TUNEL staining; 200 μm for Sirius red staining). (B) Q-RT-PCR analysis of 4-mo LFD-fed *MUP-uPA/Atf4*$^{F/F}$ and *MUP-uPA/Atf4*$^{\Delta hep}$ mice. Mean ± SD (n=6/group). *$p < 0.05$; **$p < 0.01$ (Student's t-test). (C) Serum and liver total cholesterol (T-Chol) in 6-mo HFD-fed *Atf4*$^{F/F}$, *Atf4*$^{\Delta hep}$, *MUP-uPA/Atf4*$^{F/F}$, and *MUP-uPA/Atf4*$^{\Delta hep}$ mice. Mean ± SD (n=4-6/group). *$p < 0.05$ (Student's t-test). (D) Q-RT-PCR analysis of fibrogenic cytokine and fibrosis markers in 6-mo HFD-fed *MUP-uPA/Atf4*$^{F/F}$ and *MUP-uPA/Atf4*$^{\Delta hep}$ mice. Mean ± SD (n=6-7/group). *$p < 0.05$; **$p < 0.01$ (Student's t-test). (E) Q-RT-PCR analysis of cytokine mRNAs in livers of above mice. Mean ± SD (n = 6-8/group). *$p < 0.05$; **$p < 0.01$ (Student's t-test). (F) IB analysis of liver tumors (Tu) of 10-mo HFD-fed *MUP-uPA/Atf4*$^{F/F}$ and *MUP-uPA/Atf4*$^{\Delta hep}$ mice. (G) *Atf4* genotyping in liver Non-Tu and Tu regions of above mice. Asterix and arrow mark the floxed and the deleted allele, respectively. LFD, low fat diet; HFD, high fat diet; mo, month old; T-Chol, total cholesterol; 4HNE, 4-hydroxynonenal; Tu, tumors; Non-Tu, Non-tumors.

Figure. S6

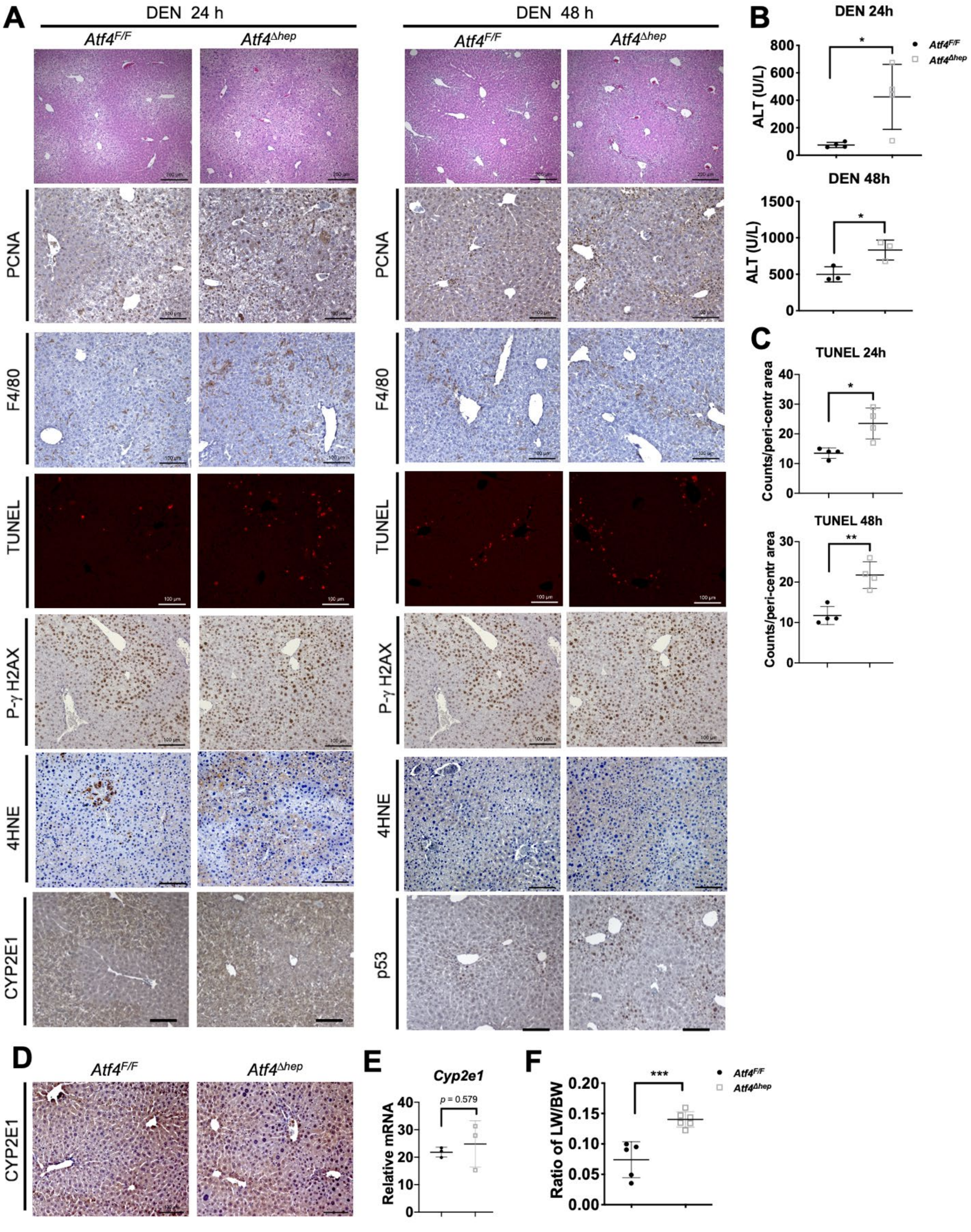
A
DEN 24 h
DEN 48 h
Atf4F/F
Atf4Δhep
PCNA
F4/80
TUNEL
P-γ H2AX
4HNE
CYP2E1
p53
B
DEN 24h
DEN 48h
ALT (U/L)
C
TUNEL 24h
TUNEL 48h
Counts/peri-centr area
D
E
Cyp2e1
p = 0.579
Relative mRNA
F
Ratio of LW/BW

Fig. S6. ATF4 ablation enhances DEN-induced acute liver injury. (A) H&E, PCNA, F4/80, TUNEL, P-H2AX, 4NHE, CYP2E1, and p53 staining of livers at 24 (left) and 48 (right) hrs after DEN-administration to 3-mo *Atf4*$^{F/F}$ and *Atf4*$^{\Delta hep}$ mice (n=4/group. Scale bars, 100 μm). (B) Serum ALT in above mice. Mean ± SD (n=4/group). **p* < 0.05 (Student's t-test). (C) Quantification of pericentral TUNEL staining of 3-mo *Atf4*$^{F/F}$ and *Atf4*$^{\Delta hep}$ mice 24 (top) or 48 (bottom) hrs after DEN administration. Mean ± SD (n=4/group). **p* < 0.05; ***p* < 0.01 (Student's t-test). (D) CYP2E1 staining of liver sections from 3-mo *Atf4*$^{F/F}$ and *Atf4*$^{\Delta hep}$ mice (n=3/group. Scale bars, 100 μm). (E) Q-RT-PCR analysis of *Cyp2e1* mRNA in livers of 3-mo *Atf4*$^{F/F}$ and *Atf4*$^{\Delta hep}$ mice. Mean ± SD (n=3/group). (F) Liver weight (LW) to body weight (BW) ratio in 10-mo DEN-treated *Atf4*$^{F/F}$ and *Atf4*$^{\Delta hep}$ mice. Mean ± SD (n=5-6/group). ****p* < 0.001 (Student's t-test). TUNEL, Terminal deoxynucleotidyl transferase dUTP nick end labeling; DEN, diethylnitrosamine; mo, month old; PCNA, proliferating cell nuclear antigen; H2AX, histone 2A member X; CYP2E1, cytochrome P450 2E1.

Figure. S7

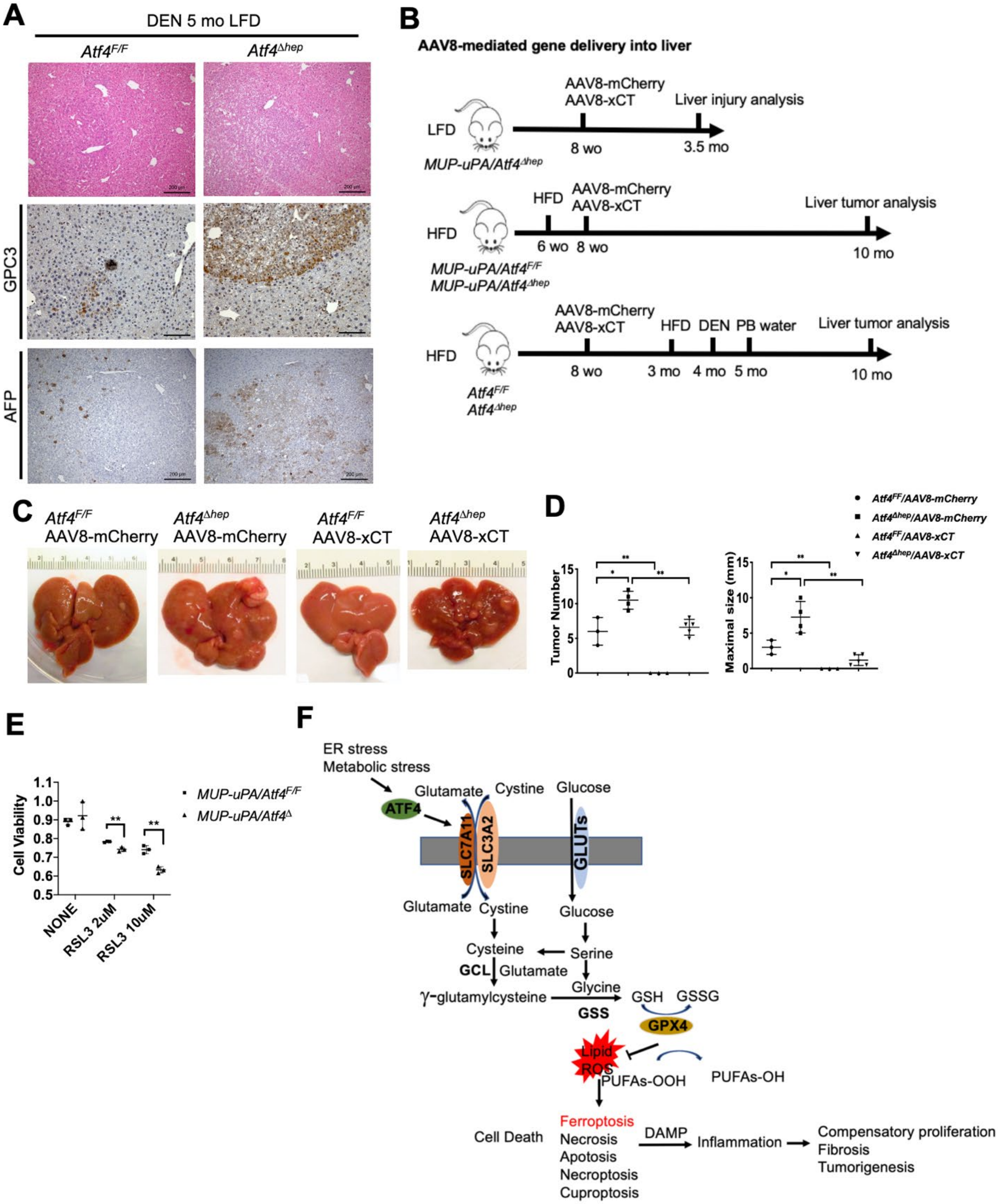
A
DEN 5 mo LFD
Atf4F/F
Atf4Δhep
GPC3
AFP
B
AAV8-mediated gene delivery into liver
AAV8-mCherry
AAV8-xCT
Liver injury analysis
LFD
MUP-uPA/Atf4Δhep
8 wo
3.5 mo
HFD
Liver tumor analysis
6 wo
10 mo
MUP-uPA/Atf4F/F
MUP-uPA/Atf4Δhep
DEN
PB water
3 mo
4 mo
5 mo
Atf4F/F
Atf4Δhep
C
Atf4F/F AAV8-mCherry
Atf4Δhep AAV8-mCherry
Atf4F/F AAV8-xCT
Atf4Δhep AAV8-xCT
D
Atf4FF/AAV8-mCherry
Atf4Δhep/AAV8-mCherry
Atf4FF/AAV8-xCT
Atf4Δhep/AAV8-xCT
Tumor Number
Maximal size (mm)
E
Cell Viability
NONE
RSL3 2uM
RSL3 10uM
MUP-uPA/Atf4F/F
MUP-uPA/Atf4Δ
F
ER stress
Metabolic stress
ATF4
Glutamate
Cystine
Glucose
SLC7A11
SLC3A2
GLUTs
Cysteine
Serine
GCL
γ-glutamylcysteine
Glycine
GSS
GSH
GSSG
GPX4
Lipid ROS
PUFAs-OOH
PUFAs-OH
Cell Death
Ferroptosis
Necrosis
Apotosis
Necroptosis
Cuproptosis
DAMP
Inflammation
Compensatory proliferation
Fibrosis
Tumorigenesis

Fig. S7. ATF4 ablation enhances DEN-induced hepatocarcinogenesis and summary. (A) H&E and IHC staining of livers from DEN-challenged 5-mo $Atf4^{FF}$ and $Atf4^{\Delta hep}$ mice (n=3-5/group. Scale bars, 100 μm). (B) Experimental treatment scheme of AAV8-mediated gene delivery. (C, D) Gross liver morphology (C) and tumor burden (D) in 10-mo HFD-fed $Atf4^{F/F}$ and $Atf4^{\Delta hep}$ mice injected with AAV8-mCherry or AAV8-xCT when 2 mo before DEN (100 mg/kg) administration when 4 mo. Mean ± SD (n=3-5/group). *$p < 0.05$; **$p < 0.01$ (Student's t-test). (E) Viability of tumor cells isolated from 6-mo HFD-fed *MUP-uPA/*$Atf4^{F/F}$ and *MUP-uPA/*$Atf4^{\Delta}$ mice treated with different doses of ferroptosis inducer RSL3. Cell viability was assessed by CCK8 assay in triplicate and the effects of each treatment were normalized to the viability of vehicle control (NONE). **$p < 0.01$ (Student's t-test). (F) ER and metabolic stress-induced ATF4-SLC7A11 signaling maintains intracellular GSH and redox homeostasis. Ablation of ATF4 reduces expression of SLC7A11 and decreases GSH, thereby enhancing ferroptosis in stressed livers. Together with necrosis, apoptosis, necroptosis, and cuproptosis, ferroptosis results in the release of damage-associated molecular patterns (DAMP), which enhance liver inflammation that contributes to compensatory proliferation, fibrosis, and tumorigenesis. Key proteins involved in glutathione metabolism and ferroptosis include SLC7A11, glutamate–cysteine ligase (GCL), GSS, and GPX4.

Supplementary Tables

Table S1: Antibodies

Name	Citation	Supplier	Cat no.	Clone no.
Guinea pig anti-mouse/human p62/SQSTM1 (C-terminus)	RRID:AB_2687531	Progen	GP62-C	NA
Mouse monoclonal anti-human p62/SQSTM1	RRID:AB_2800125	Cell Signaling Technologies	88588	D5L7G
Mouse monoclonal anti-mCherry	RRID:AB_11133266	Abcam	ab125096	1C51
Rabbit monoclonal anti-NQO1	RRID:AB_2799623	Cell Signaling Technologies	62262	D6H3A
Rabbit monoclonal anti-NQO1	RRID:AB_2154354	Cell Signaling Technologies	3187	A180
Rabbit polyclonal anti-mouse/human p44/42 MAPK (Erk1/2)	RRID: AB_330744	Cell Signaling Technologies	9102	NA
Rabbit monoclonal anti-ATF4 (D4B8)	RRID:AB_2616025	Cell Signaling Technologies	11815	D4B8
Rabbit polyclonal anti-ATF4	RRID:AB_2058600	Proteintech	10835-1-AP	NA
Rabbit polyclonal anti-Phospho-p44/42MAPK (Erk1/2) (Thr202/ Tyr204)	RRID: AB_331646	Cell Signaling Technologies	9101	NA
Rabbit monoclonal anti-mouse/human Ki67	RRID: AB_422351	GeneTex	GTX16667	SP6
Rabbit monoclonal anti-NRF2 (D1Z9C) XP	RRID:AB_2715528	Cell Signaling Technologies	12721	D1Z9C
Rabbit polyclonal anti-Phospho-Stat3 (Tyr705) Antibody #9131	RRID:AB_331586	Cell Signaling Technologies	9131	NA
Rabbit monoclonal anti- Stat3 (D3Z2G) antibody	RRID: AB_2629499	Cell Signaling Technologies	12640	D3Z2G
Rabbit monoclonal anti-Phospho-eIF2α (Ser51) (119A11)	RRID: AB_390740	Cell Signaling Technologies	3597	119A11
Rabbit monoclonal anti-eIF2alpha (D7D3) XP	RRID:AB_10692650	Cell Signaling Technologies	5324	D7D3
Rabbit Polyclonal HDAC1 (H-51) antibody	RRID: AB_2279709	Santa Cruz Biotechnology	7872	NA
Rabbit Polyclonal anti-Phospho-SAPK/JNK (Thr183/Tyr185) Antibody	RRID:AB_331659	Cell Signaling Technologies	9251	NA
Mouse monoclonal anti-PCNA	RRID:AB_397992	BD Biosciences	610665	24
Mouse monoclonal anti-JNK1/JNK2 antibody	RRID:AB_395344	BD Biosciences	554285	G151-666
Rabbit Polyclonal anti-GRP78 (H-129) antibody	RRID: AB_2119991	Santa Cruz Biotechnology	13968	NA
Mouse monoclonal anti-ERp5	RRID:AB_10843798	Santa Cruz Biotechnology	365260	G5
Mouse monoclonal anti-CHOP (L63F7)	RRID: AB_2089254	Cell Signaling Technologies	2895	L63F6
Rabbit polyclonal anti-Phospho-Akt (Ser473)	RRID:AB_329825	Cell Signaling Technologies	9271	NA

Rabbit monoclonal anti-Phospho-Akt (Ser473) (D9E)	RRID:AB_2315049	Cell Signaling Technologies	4060	D9E
Mouse monoclonal anti-alpha-Tubulin	RRID:AB_477579	Sigma-Aldrich	T-5168	B-5-1-2
Mouse monoclonal anti-Akt1/2/3	RRID:AB_1118808	Santa Cruz Biotechnology	81434	5C10
Rabbit Polyclonal anti-Phospho-p38 MAPK (Thr180/Tyr182) Antibody	RRID:AB_331641	Cell Signaling Technologies	9211	NA
Rabbit Polyclonal anti-xCT	RRID:AB_2800296	Cell Signaling Technologies	98051	NA
Rabbit monoclonal anti-Phospho-Histone H2A.X (Ser139)	RRID:AB_2118009	Cell Signaling Technologies	9718	20E3
Rabbit Polyclonal anti-p53	RRID:AB_563933	Leica Biosystems	NCL-p53-CM5p	NA
Rabbit Polyclonal anti-P38 alpha	RRID:AB_632138	Santa Cruz Biotechnology	435	NA
Rabbit monoclonal anti-Akt (Pan) (C67E7)	RRID:AB_915783	Cell Signaling Technologies	4691	C67E7
Mouse monoclonal anti- Actin	RRID:AB_476730	Sigma-Aldrich	A4700	AC-40
Horse anti-mouse IgG, HRP-linked Antibody	RRID:AB_330924	Cell Signaling Technologies	7076	NA
Goat anti-rabbit IgG, HRP-linked Antibody	RRID:AB_2099233	Cell Signaling Technologies	7074	NA
Rabbit Polyclonal anti-Glypican 3	RRID:AB_1141042	Abcam	ab66596	NA
Mouse Monoclonal Anti-4-Hydroxy-2-Nonenal Antibody	RRID:AB_867452	Abcam	ab48506	HNEJ-2
Goat Polyclonal anti-Mouse alpha-Fetoprotein	RRID: AB_2258018	R&D Systems	AF5369	NA
Rat Monoclonal anti-F4/80	RRID:AB_2277854	Thermo Fisher	MA1-91124	A3-1
Rabbit Polyclonal anti-Cytochrome P450 Enzyme CYP2E1	RRID:AB_11212002	Millipore	AB1252	NA
Rabbit Polyclonal anti-TRB3	RRID:AB_2200966	Millipore	ST1032	NA
Goat anti-Guinea Pig IgG, HRP-linked Antibody	RRID:AB_2535546	Thermo Fisher Scientific	A18769	NA
Biotin Goat anti-Mouse Ig (IHC)	RRID: AB_395196	BD Pharmingen	553999	NA
Biotin Goat anti-Rabbit Ig (IHC)	RRID: AB_393618	BD Pharmingen	550338	NA

Table S2: Primers used for Q-RT-PCR

Primer name	Primer sequence
Mouse *Hprt1*_Forward	ACCTGGTTCATCATCGCTAA
Mouse *Hprt1*_Reverse	CTCCTCAGACCGCTTTTTG
Mouse *Nqo1*_Forward	AGCGTTCGGTATTACGAT CC
Mouse *Nqo1*_Reverse	AGTACAATCAGGGCTCTTCTCG
Mouse *Atf4*_Forward	TGACCGAGATGAGCTTCCTGA
Mouse *Atf4*_ Reverse:	GAGAACCCATGAGGTTTCAAGTG
Mouse *Erp5*_Forward	TCACTAGAAGAATACAGACCGCTG
Mouse *Erp5*_Reverse	GGACAGAATGCGGGTGAT
Mouse *Grp78/Bip*_Forward	GGTGCAGCAGGACATCAAGTT
Mouse *Grp78/bIP*_Reverse	CCCACCTCC AATATCAACTTG A
Mouse *Mdm2*_Forward	GCGTGGAATTTGAAGTTGAGTC
Mouse *Mdm2*_Reverse	CTGTATCGCTTTCTCCTGTCTG
Mouse *Chop*_Forward	CTGCCTTTCACCTTGGAGAC
Mouse *Chop*_Reverse	CGTTTCCTGGGGATGAGATA
Mouse *Edem2*_Forward	CATTGTCCTGAAGAACCTCCA
Mouse *Edem2* _Reverse	ACCTGGGGCAGTTTTTCTCT
Mouse *Tnf*_Forward	AGGGTCTGGGCCATAGAACT
Mouse *Tnf*_Reverse	CCACCACGCTCTTCTGTCTAC
Mouse *F4/80*_Forward	GGATGTACAGATGGGGGATG
Mouse *F4/80* _Reverse	GTCTGTGGTGTCAGTGCAGG
Mouse *Il1a*_Forward	ATGTATGCCTACTCGTCGGG
Mouse *Il1a*_Reverse	TGAGTTTTGGTGTTTCTGGC
Mouse *Mthfd2*_Forward	ACAGATGGAGCTCACGAACG
Mouse *Mthfd2*_Reverse	TGCCAGCGGCAGATATTACA
Mouse *Gls2*_Forward	AGCGTATCCCTATCCACAAGTTCA
Mouse *Gls2*_Reverse	GCAGTCCAGTGGCCTTCAGAG
Mouse *Glud1*_Forward	AGCCAGTGCTTTTACTTCATCC
Mouse *Glud1*_Reverse	GGGAGGTCATCGAAGGCTAC
Mouse *Phgdh*_Forward	GCACACCTTTCTTGCACTGA
Mouse *Phgdh*_Reverse	GGAGGAGATCTGGCCTCTCT
Mouse *Psat1*_Forward	TTGATCCATTCCAGGACCAT
Mouse *Psat1* _Reverse	ACTACAAAGTGCAGGCTGGG
Mouse *Chac1*_Forward	AGGTACTTCAGGGCCTCGTT
Mouse *Chac1* _Reverse	GTGGTGACCCTCCTTGAAGA
Mouse *Pck2*_Forward	AGTTTGGATGTGCACAGGGT
Mouse *Pck2* _Reverse	GTACTGGGAAGGCATTGACC
Mouse *Psph*_Forward	TCTGAGTGGGAGACCATCCT
Mouse *Psph* _Reverse	CCCACTGCTACCGGAAAGT
Mouse *Fgf21*_Forward	CTCCAGCAGCAGTTCTCTGA
Mouse *Fgf21* _Reverse	CCTGGGTGTCAAAGCCTCTA
Mouse *Cars*_Forward	GCCAGTCAGAGAGCAAGTCC
Mouse *Cars* _Reverse	CAGAACCACTGGAACAGGCT
Mouse *Asns*_Forward	TCACCATCCACATTGGTCTG
Mouse *Asns* _Reverse	TTTGTGGCTCTGTTACAATGG
Mouse *Slc1a5*_Forward	CTCATGTAAAATACCGCAATCCTGT
Mouse *Slc1a5* _Reverse	TCATTCCCTCCACCTCACAGA
Mouse *Slc7a5*_Forward	CTGGTCTTCGCCACCTACTT
Mouse *Slc7a5* _Reverse	GCCTTTACGCTGTAGCAGTTC
Mouse *Ptgs2*_Forward	CTGCGCCTTTTCAAGGATGG
Mouse *Ptgs2* _Reverse	GGGGATACACCTCTCCACCA

Mouse *Slc1a4*_Forward	CAGAAACCTGTTCCCTTCCA
Mouse *Slc1a4* _Reverse	ACGGGGATCTTCTCTTTGGT
Mouse *Gss*_Forward	GTGAATGGGGCATACGTCA
Mouse *Gss*_Reverse	CAAAGCAGGCCATAGACAGG
Mouse *Ho1*_Forward	CCTTCAAGGCCTCAGACAAA
Mouse *Ho1*_Reverse	GAGCCTGAATCGAGCAGAAC
Mouse *Gclm*_Forward	TTGGGAACTCCATTCATTCA
Mouse *Gclm* _Reverse	CGGGAACCTGCTCAACTG
Mouse *Slc7a11*_ Forward	TCTGGTCTGCCTGTGGAGTA
Mouse *Slc7a11*_ Reverse	CAAAGGACCAAAGACCTCCA
Mouse *Shmt2*_Forward	ATGCAGACCAGCTGACCAC
Mouse *Shmt2*_Reverse	AGTGGCTAGTCCTCCTGTGC
Mouse *Shmt1*_Forward	GCTGCGACAACATCTTCTCAT
Mouse *Shmt1*_Reverse	CCTTTTCACAAAATCCACGC
Mouse *Cth*_Forward	CAGAGGCATGGGTCATGATT
Mouse *Cth*_Reverse	TGCTAAGGCCTTCCTCAAAA
Mouse *Bax*_Forward	GATCAGCTCGGGCACTTTAG
Mouse *Bax*_Reverse	TTGCTGATGGCAACTTCAAC
Mouse *Dr5*_Forward	GGTCCTCTTGATGGGCTCTC
Mouse *Dr5*_Reverse	GTTGCTGCTTGCTGTGCTAC
Mouse *Bid*_Forward	GTGTAGCTCCAAGCACTGCC
Mouse *Bid*_Reverse	GCAAACCTTTGCCTTAGCC
Mouse *Fas*_Forward	CCTCAGCTTTAAACTCTCGGA
Mouse *Fas*_Reverse	CAGACATGCTGTGGATCTGG
Mouse *Puma*_Forward	TGTCGATGCTGCTCTTCTTG
Mouse *Puma*_Reverse	GTGTGGAGGAGGAGGAGTGG
Mouse *Ly6g*_Forward	TTGCAAAGTCCTGTGTGCTC
Mouse *Ly6g*_Reverse	AGGGGCAGGTAGTTGTGTTG
Mouse *Ccl2*_Forward	ATGAGATCAGAACCTACAACT
Mouse *Ccl2*_Reverse	TCCTACAGAAGTGCTTGAG
Mouse *Ccl19*_Forward	CTTCAGCCT GCTGGTTCTCT
Mouse *Ccl19*_Reverse	GGAAGGCTTTCACGATGTTC
Mouse *Il23a*_Forward	GCTCCCCTTTGAAGATGTCA
Mouse *Il23a*_Reverse	GACCCACAAGGACTCAAGGA
Mouse *Slc3a2*_Forward	TGCTCAGGCTGACATTGTAGC
Mouse *Slc3a2*_Reverse	TCAGCCAAGTACAAGGGTGC
Mouse *Sars*_Forward	AGTTGTCTGCCCGAAATCTG
Mouse *Sars*_Reverse	ATTCGAGAGACGCAGGAGAA
Mouse *Cd44*_Forward	CAGAGGCGACTAGATCCCTC
Mouse *Cd44*_Reverse	GAGTCACAGTGCGGGAACTC
Mouse *Gpc3*_Forward	CCCTGAATCTCGGAATTGAA
Mouse *Gpc3*_Reverse	AGTCCCTGGCAGTAAGAGCA
Mouse *Epcam*_Forward	CTGGCGTCTAAATGCTTGGC
Mouse *Epcam*_Reverse	TCGTACAGCCCATCGTTGTTC
Mouse *Timp1*_Forward	GCAACTCGGACCTGGTCATAA
Mouse *Timp1*_Reverse	CGGCCCGTGATGAGAAACT
Mouse *Col1a1*_Forward	GCTCCTCTTAGGGGCCACT
Mouse *Col1a1*_Reverse	CCACGTCTCACCATTGGGG
Mouse *Acta2*_Forward	GTCCCAGACATCAGGGAGTAA
Mouse *Acta2*_Reverse	TCGGATACTTCAGCGTCAGGA
Mouse *Il1b*_Forward	GGTCAAAGGTTTGGAAGCAG
Mouse *Il1b*_Reverse	TGTGAAATGCCACCTTTTGA
Mouse *Ccnd1*_Forward	TTGTGCATCTACACTGACAACTC

Mouse *Ccnd1*_Reverse	AGGGTGGGTTGGAAATGAACT
Mouse *Il6*_Forward	CGCTATGAAGTTCCTCTCTGC
Mouse *Il6*_Reverse	TCTGCAAGTGCATCATCGTT
Mouse *Tgfb*_Forward	GGAGAGCCCTGGATACCAAC
Mouse *Tgfb*_Reverse	AAGTTGGCATGGTAGCCCTT
Mouse *Dlk1*_Forward	GGC CAT CGT CTT TCT CAA CA
Mouse *Dlk1*_Reverse	CTC CTC ATC ACC AGC CTC CT
Mouse *Ctgf*_Forward	TGACCTGGAGGAAAACATTAAGA
Mouse *Ctgf*_Reverse	AGCCCTGTATGTCTTCACACTG
Mouse *Col4a1*_Forward	CTGGCACAAAAGGGACGAG
Mouse *Col4a1*_Reverse	ACGTGGCCGAGAATTTCACC

Supplementary References

[1] He F, Antonucci L, Yamachika S, Zhang Z, Taniguchi K, Umemura A, et al. NRF2 activates growth factor genes and downstream AKT signaling to induce mouse and human hepatomegaly. J Hepatol 2020;72:1182-1195.

[2] Sheehan DC, Hrapchak BB. Theory and practice of histotechnology, 2d ed. St. Louis: Mosby; 1980.

[3] Moreno AM, Fu X, Zhu J, Katrekar D, Shih YV, Marlett J, et al. In Situ Gene Therapy via AAV-CRISPR-Cas9-Mediated Targeted Gene Regulation. Mol Ther 2018;26:1818-1827.
